# Alpha-linolenic acid modulates systemic and adipose tissue-specific insulin sensitivity, inflammation, and the endocannabinoid system in dairy cows

**DOI:** 10.1038/s41598-023-32433-7

**Published:** 2023-03-31

**Authors:** Gitit Kra, Jayasimha Rayalu Daddam, Uzi Moallem, Hadar Kamer, Batel Mualem, Yishai Levin, Radka Kočvarová, Alina Nemirovski, Andres G. Contreras, Joseph Tam, Maya Zachut

**Affiliations:** 1 Department of Ruminant Science, Institute of Animal Sciences, ARO Volcani Institute, Rishon LeZiyon, Israel; 2 Department of Animal Science, The Robert H. Smith Faculty of Agriculture, Food and Environment, The Hebrew University of Jerusalem, Rehovot, Israel; 3 The Nancy and Stephen Grand Israel National Center for Personalized Medicine, Weizmann Institute of Science, Rehovot, Israel; 4 Obesity and Metabolism Laboratory, Faculty of Medicine, School of Pharmacy, The Institute for Drug Research, The Hebrew University of Jerusalem, Jerusalem, Israel; 5 Department of Large Animal Clinical Sciences, College of Veterinary Medicine, Michigan State University, East Lansing, USA

**Keywords:** Molecular biology, Inflammation, Mass spectrometry, Proteomic analysis, Cell signalling, Physiology, Metabolism, Fat metabolism

## Abstract

Metabolic disorders are often linked to alterations in insulin signaling. Omega-3 (n-3) fatty acids modulate immunometabolic responses; thus, we examined the effects of peripartum n-3 on systemic and adipose tissue (AT)-specific insulin sensitivity, immune function, and the endocannabinoid system (ECS) in dairy cows. Cows were supplemented peripartum with saturated fat (CTL) or flaxseed supplement rich in alpha-linolenic acid (ALA). Blood immunometabolic biomarkers were examined, and at 5–8 d postpartum (PP), an intravenous glucose-tolerance-test (GTT) and AT biopsies were performed. Insulin sensitivity in AT was assessed by phosphoproteomics and proteomics. Peripartum n-3 reduced the plasma concentrations of Interleukin-6 (IL-6) and IL-17α, lowered the percentage of white blood cells PP, and reduced inflammatory proteins in AT. Systemic insulin sensitivity was higher in ALA than in CTL. In AT, the top canonical pathways, according to the differential phosphoproteome in ALA, were protein-kinase-A signaling and insulin-receptor signaling; network analysis and immunoblots validated the lower phosphorylation of protein kinase B (Akt), and lower abundance of insulin receptor, together suggesting reduced insulin sensitivity in ALA AT. The n-3 reduced the plasma concentrations of ECS-associated ligands, and lowered the abundances of cannabinoid-1-receptor and monoglycerol-lipase in peripheral blood mononuclear cells PP. Peripartum ALA supplementation in dairy cows improved systemic insulin sensitivity and immune function, reduced ECS components, and had tissue-specific effects on insulin-sensitivity in AT, possibly counter-balancing the systemic responses.

## Introduction

Metabolic disorders in humans as well as in companion and production animals are often linked to an alteration in insulin signaling. Many pharmacological interventions have been developed; however, their secondary effects have limited their use in large groups of patients. In contrast, nutritional interventions such as the targeted supplementation of bioactive fatty acids, for instance, omega-3 (n-3) fatty acids (FA), can exert immunometabolic effects^1^ and can modulate insulin sensitivity in animal and human models^2–4^.

Insulin plays an important role in maintaining glucose homeostasis in mammals, especially around parturition and the onset of lactogenesis^5^. In mammals, insulin sensitivity decreases during gestation^6,7^; in diabetic women, it was demonstrated that insulin sensitivity is increased immediately after birth, but that it is then reduced within the first 6 months postpartum (PP). Dairy cows modify their metabolism, immune system, and inflammatory responses after calving to adjust to parturition and the onset of lactation. When the nutrient demand exceeds the food intake PP, the homeorethic shift to enhance milk production leads to a condition of negative energy balance that is manifested by increased insulin resistance, systemic inflammation, and adipose tissue (AT) remodeling^1,8^. Following an increase in blood glucose, insulin is secreted from pancreatic beta cells into the bloodstream. Within peripheral tissues that are responsive to insulin, such as the AT, insulin binds to the insulin receptor, which initiates a cascade of intra-cellular phosphorylation events of proteins such as insulin receptor substrate (IRS), which interacts with phosphatidylinositol-3-kinase (PI3K) to increase the phosphorylation of protein kinase B (Akt)^9–12^. This cascade of events leads to translocation of glucose transporter 4 (GLUT4) and glucose uptake into the cells. Thus, when the PI3K/Akt signaling pathway is disrupted, insulin resistance may develop^13,14^. During inflammation, the AT synthesizes the pro-inflammatory cytokines tumor necrosis factor alpha (TNF-α) and interleukin-6 (IL-6), which may alter the expression or activity of insulin-signaling molecules, such as IRS-1 and GLUT4 in adipocytes. Thus, inflammation in AT may be associated with insulin resistance/sensitivity^15–17^.

Protein phosphorylation is central to insulin’s signal transduction in all mammalian cell types including adipocytes. Mass spectrometry-based phospho-proteomics of in-vitro adipocytes was previously performed^18,19^; however, the mechanism by which dietary components may affect insulin signaling in the AT, according to the phosphoproteome following glucose stimulation, remains to be explored. Phospho-proteomics is used to quantify the plethora of phosphopeptides in a tissue at a given state. We have previously demonstrated that peripartum supplementation of conjugated linoleic acid affects the phosphoproteome of unstimulated AT in dairy cows by increasing lipid metabolism^20^. However, currently there is no information on the phosphoproteome of glucose-stimulated AT in livestock.

Nutritional supplementation of n-3 FA reduces the ratio of omega-6 (n-6)/n-3 in blood and tissues^21^, exerts anti-inflammatory effects^22–24^, and can affect insulin sensitivity in animal and human models^25–27^. Interestingly, n-3 supplementation also reduces the activation of the endocannabinoid system (ECS) by limiting the availability of n-6 FA arachidonic acid (C20:4n-6), which is an endocannabinoid precursor^25^. We have recently demonstrated that peripartum supplementation of the n-3 FA alpha linolenic acid (ALA) affects immunometabolic responses and ECS activity in dairy cows^26^; however, the effects of ALA supplementation on insulin sensitivity have not yet been explored in livestock. In humans, activation of the ECS is associated with the induction of insulin resistance in AT, since ECS-related genes were found to be up-regulated in subcutaneous and abdominal AT of obese human patients, and activation of cannabinoid-1 receptor (CB1) stimulates glucose uptake in primary human adipocytes^27^. Furthermore, CB1 stimulation increases insulin levels in vitro and in obese mice^27–30^. Nevertheless, it is not known how modulating the ECS by ALA supplementation would affect insulin signaling in healthy PP cows.

We hypothesized that peripartum ALA supplementation would reduce the n-6/n-3 ratio in blood, induce anti-inflammatory effects, reduce ECS activation, and increase insulin sensitivity in PP dairy cows. We examined the effects of peripartum ALA supplementation on immunometabolic indices, insulin sensitivity, and ECS components both systemically and in the AT of dairy cows. In addition, we examined the molecular mechanism underlying altered insulin sensitivity in AT by utilizing novel phospho-proteomics and proteomics. Our data support the hypothesis and provide novel insights on the effects of peripartum n-3 supplementation on immunometabolism in dairy cows.

## Results

### ALA decreased the n-6/n-3 ratio in plasma, reduced feed intake, and had moderate effects on metabolic responses PP

Supplementation of dietary n-3 rich in ALA increased the plasma percentages of ALA (C18:3n-3; *P* < 0.001), C20:4n-3 (*P* = 0.01), and total n-3 FA (*P* < 0.01) in plasma, compared with controls; thus, the n-6/n-3 ratio in blood was lower in ALA than in CTL (*P* < 0.01; Supplementary Table 3). During the first 21 d PP, the average feed intake was lower in ALA cows than in CTL cows (23.0 and 25.4 kg dry matter/day, respectively; SEM = 0.46; *P* = 0.001), whereas milk yield (37.5 and 36.9 kg/day) in CTL and ALA cows, respectively; SEM = 7.82, *P* = 0.79) and milk components were similar; therefore, the calculated energy balance was lower in ALA vs. CTL cows (-3.3 and 0.6 Mcal/day, respectively; SEM = 1.05; *P* = 0.02). The average body weight loss between weeks 1 and 5 PP was not different between groups (30.6 and 26.5 kg, in CTL and ALA respectively, SEM = 4.94, *P* = 0.6); and body condition scores PP were similar between groups (Supplementary Fig. 1). Overall, no differences were observed PP in plasma non-esterified fatty acids (NEFAs), triglycerides, aspartate aminotransferase (AST) activity, insulin, and cortisol, whereas β-hydroxybutyric acid (BHBA) was lower in the ALA than in the CTL cows (*P* = 0.02; Supplementary Table 4).

### ALA attenuated immune function and ECS components in PBMC

**Figure 1:**
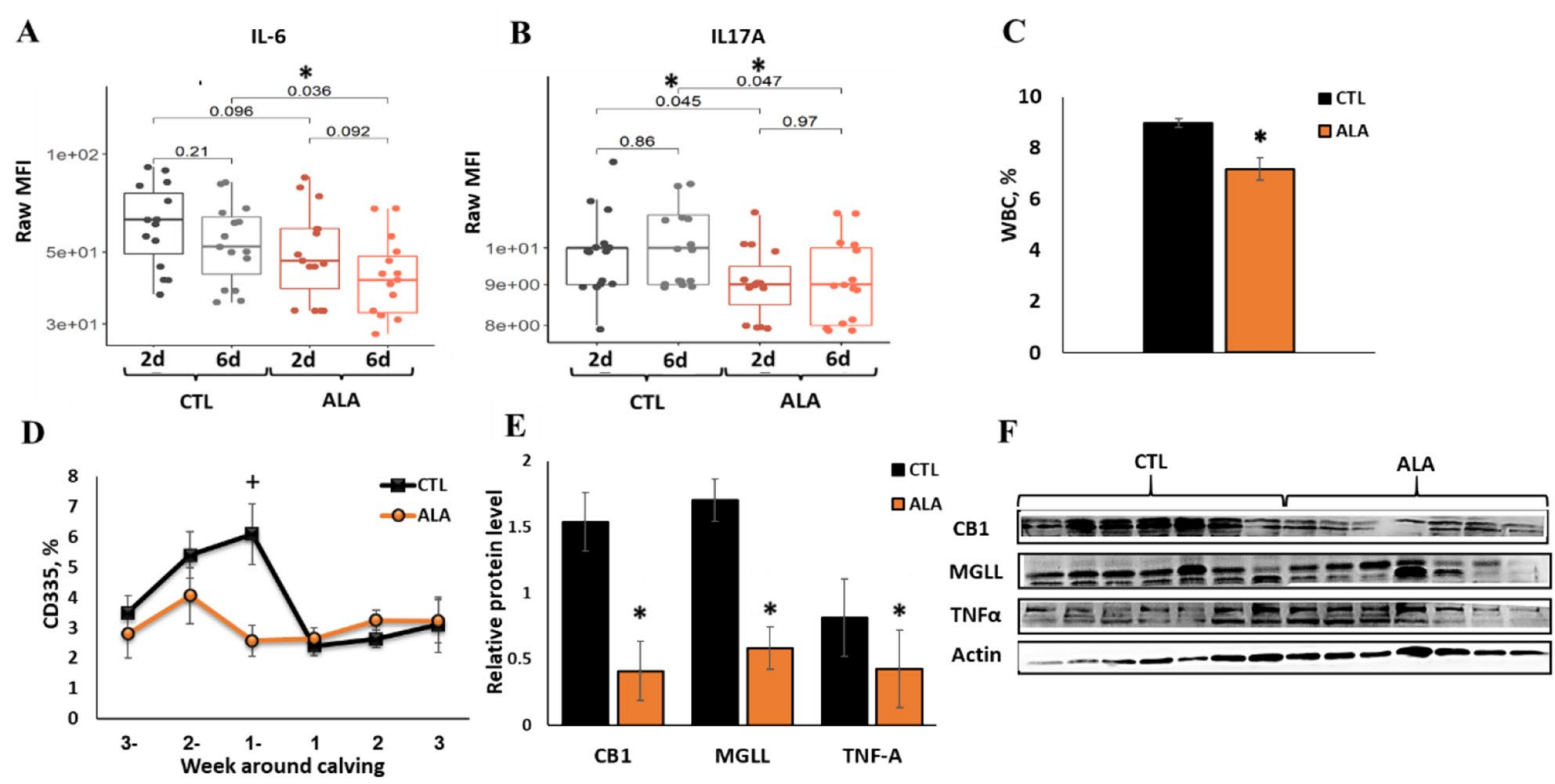
Immune factors in plasma, white blood cells (WBC), and the peripheral blood mononuclear cells (PBMCs) of postpartum dairy cows supplemented with ALA during the peripartum period. Dairy cows at 257 days of pregnancy were divided into two nutritional groups supplemented with (i) CTL (n = 16)—encapsulated saturated fat, (ii) ALA (n = 12)—flaxseed supplement providing α-linolenic acid (ALA). (**A**) interleukin–6 (IL6) at days 2 and 6 postpartum (PP); (**B**) Plasma concentrations of Interleukin-17A (IL17A) at days 2 and 6 PP; (**C**) WBC percentage in blood according to CBC; (**D**) Flow cytometry analysis of WBC subpopulation CD335 (Natural killer cells) in blood; (**E,F**) the relative protein abundance in PBMCs (at 10 d PP, n = 7); *CB1* Cannabinoid-1 receptor, *MGLL* monoglycerol lipase, *TNF-A* tumor necrotic factor α; β-Actin was used as a reference protein. Data are presented as the mean ± SEM.

During the first week PP, the plasma concentrations of interlukin-6 (IL-6; *P* = 0.04) and IL-17A (*P* = 0.05) were lower in ALA than in CTL cows (Fig. 1A,B), and the concentrations of the chemokines CCL4 (*P* = 0.08) and CCL2 (*P* = 0.12) tended to be lower in ALA than in CTL cows. The average concentrations of interleukins IL-1α, IL-1β, IL-1RA, IL-2, IL-4, and interferon γ (IFNγ), as well as the chemokines CCL3, CXCL8, CXCL10, and tumor necrosis factor alpha (TNFα) did not differ between groups (data not shown). Postpartum, a decrease in the white blood cell percentage (WBC; *P* < 0.001, Fig. 1C), and an increase in the percentage of basophiles (*P* = 0.03), hemoglobin (HGB; *P* = 0.03), and hematocrit (HCT; *P* = 0.01) were found in the blood of ALA compared with CTL cows (Supplementary Table 5). In addition, the percentage of CD335 (natural killer) T cells in the blood of ALA cows tended to be lower at one week prepartum compared with that of CTL cows (*P* = 0.1; Fig. 1D); however, no differences were observed between groups regarding the percentages of CD4, CD8, CD25, and WC1 cells in blood (data not shown).

In peripheral blood mononuclear cells (PBMC) collected at 10 d PP, the protein abundances of the ECS components cannabinoid-1 receptor (CB1; *P* = 0.004) and monoglycerol lipase (MGLL; *P* = 0.02) were lower, and TNF-α tended to be lower in ALA than in CTL cows (*P* = 0.08; Fig. 1E,F).

### ALA increased systemic insulin sensitivity, whereas AT insulin signaling decreased

**Figure 2:**
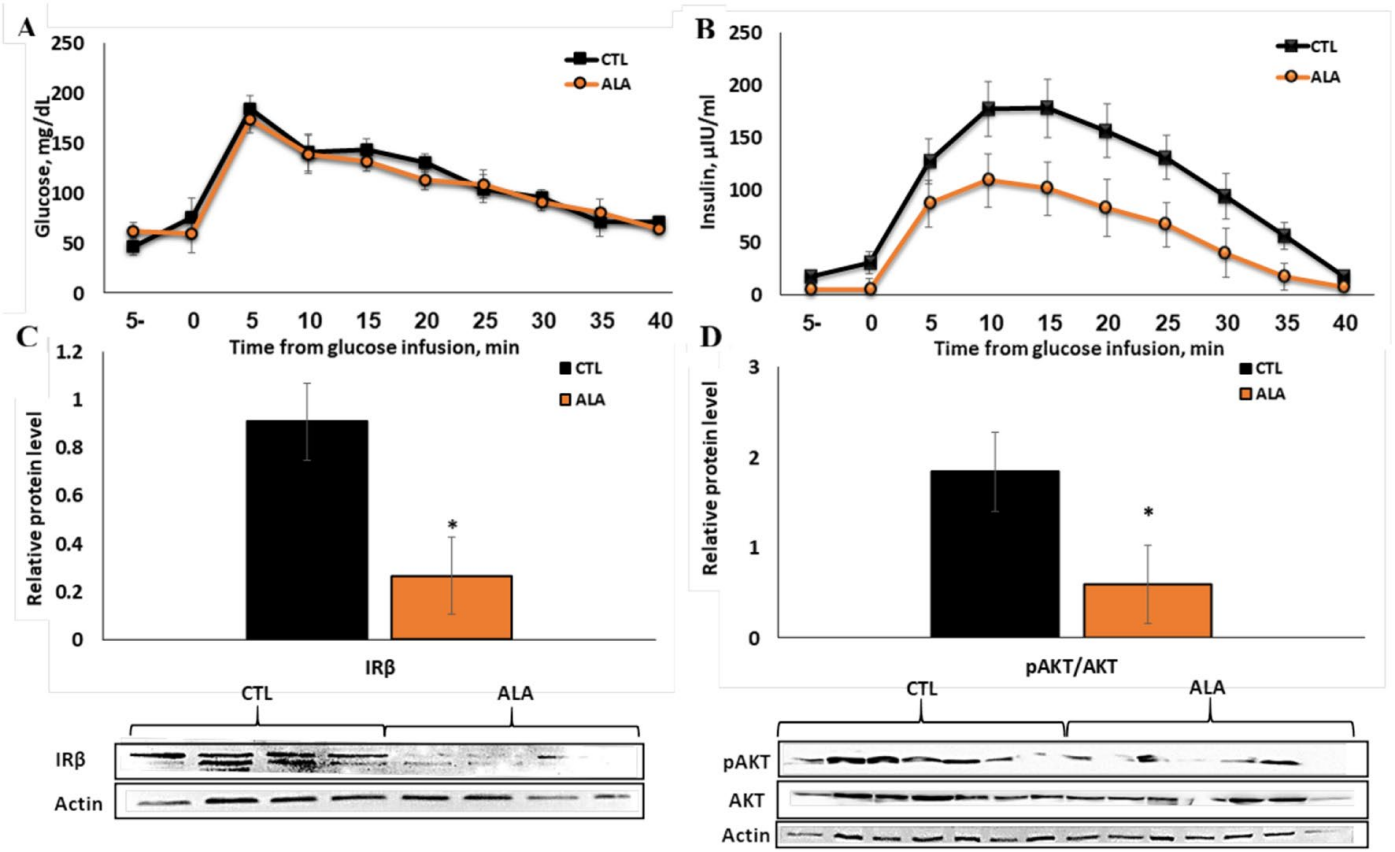
Response to the glucose tolerance test (GTT) in postpartum (PP) dairy cows (5–8 days PP) supplemented with n-3 during the peripartum period. Dairy cows at 257 days of pregnancy were divided into two nutritional groups supplemented with (i) CTL—encapsulated saturated fat, (ii) ALA—flaxseed supplement providing α-linolenic acid (ALA). n = 7. Time 0—Glucose infusion. (**A**) Plasma glucose concentrations during GTT; (**B**) plasma insulin concentrations during GTT; (**C**) protein abundance of insulin receptor beta (IRβ; n = 4) in insulin-stimulated AT; (**D**) phosphorylation of protein kinase B (pAkt; n = 7) in insulin-stimulated AT; two ALA samples were excluded from the analysis, one due to low protein levels and one due to ketosis diagnosed at 10 d PP. Data are presented as the mean ± SEM.

Following glucose infusion, glucose clearance from blood was similar between groups (Fig. 2A), and glucose half-life (33.7 and 36.8 min in CTL and ALA, respectively, SEM = 6.43, *P* = 0.74) and the areas under the curve (AUC) were similar (192.9 and 176.5 mg in CTL and ALA, respectively, SEM = 12.60, *P* = 0.35). However, the insulin concentrations and AUC (197.7 and 109.3 ng in CTL and ALA cows, respectively; SEM = 27.70; *P* = 0.05; Fig. 2B) were significantly lower in ALA than in CTL cows, thus indicating a higher systemic insulin sensitivity in ALA cows.

In the glucose-stimulated AT, the average protein abundance of the insulin receptor beta subunit (IRβ) was twofold lower in ALA than in CTL (*P* = 0.03, Fig. 2C), and the ratio of phosphorylated protein kinase B (pAkt) to general Akt (pAkt/gAkt) was 1.7-fold lower in ALA than in CTL AT (*P* = 0.03, Fig. 2D).

### ALA affects the phospho-proteome and the proteome of insulin-stimulated adipose

**Figure 3:**
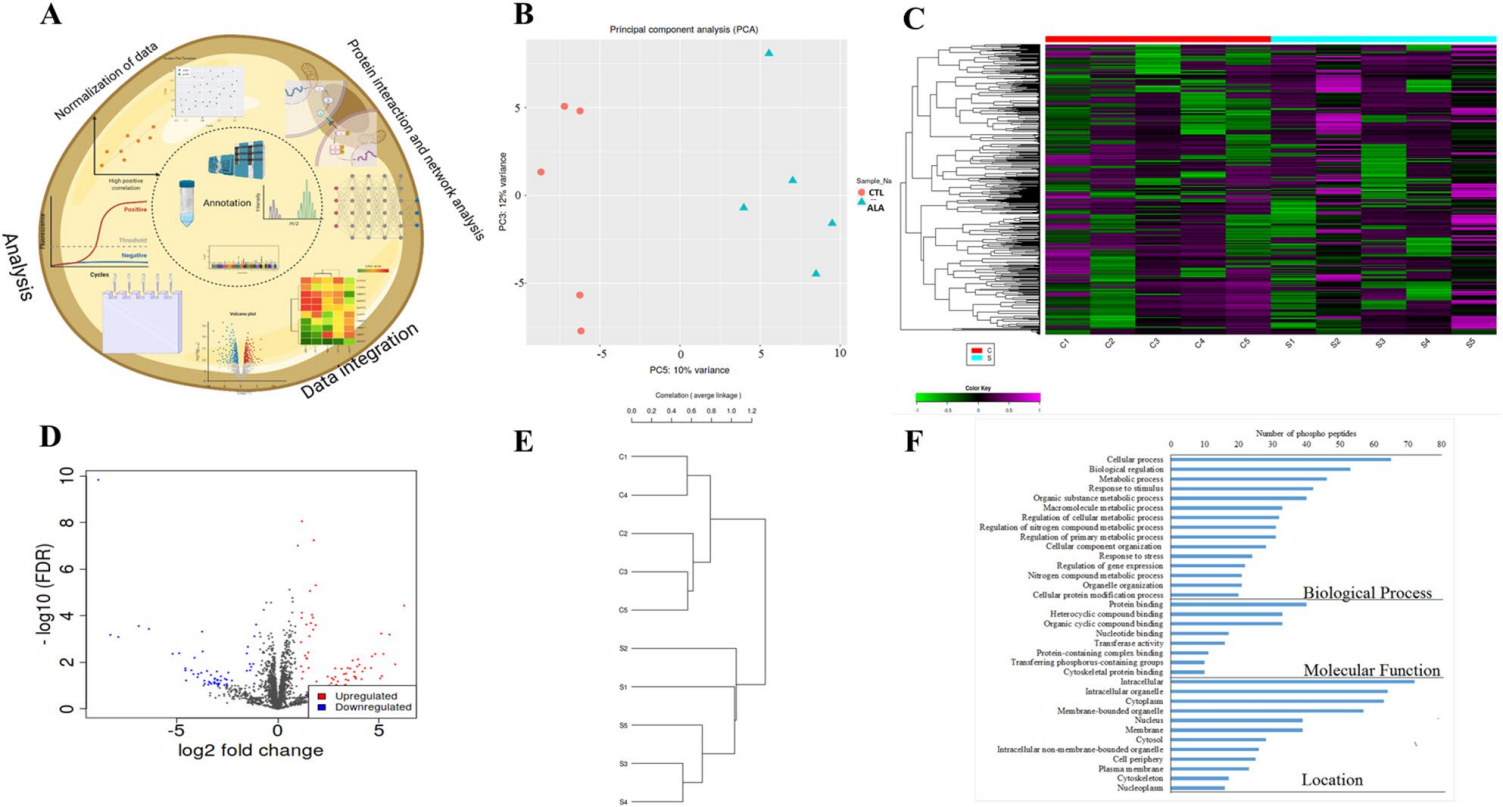
Phospho-proteomic analysis of insulin-stimulated adipose tissue from postpartum (PP) of dairy cows supplemented peripartum with ALA. Dairy cows were divided into two groups from 21 to 60 days PP; (i) CTL—encapsulated saturated fat, (ii) ALA—flaxseed supplement providing α-linolenic acid (ALA). n = 5 per treatment. (**A**) Work flow diagram of phospho-proteome analysis. (**B**) Principal component analysis (PCA) of AT phospho-proteome; CTL samples are denoted as red circles, whereas ALA samples are denoted as blue triangles. (**C**) Heat map analysis of AT phospho-proteome, low phosphopeptide intensity is denoted in green, whereas high intensity is denoted in purple. Each cow in the study is numbered and represented in columns. (**D**) Volcano plot analysis of AT phospho-proteome; the *P* value (≤ 0.05) is represented on the Y-axis and FDR (± 1.5) is represented on the X-axis. Each dot represents one protein; red indicates more, whereas blue indicates fewer phosphoproteins in AT. (**E**) Correlation of CTL vs ALA adipose generated by the IDEP9.5 server where c1, c2, c3, c4, and c5 represent CTL; s1, s2, s3, s4, and s5 represent ALA-supplemented cows. (**F**) GO analysis of AT phosphoproteome supplemented with ALA including the biological process category, the molecular function category, and the cell component category. Image was generated using www.Biorender.com.

In the phosphoproteomics (Fig. 3A), PCA showed 10% variance and the treatment groups were well clustered; indicating a change in the phosphoproteome (Fig. 3B). In total, 3501 phosphopeptides were identified in AT, of which 169 were significantly different between ALA and CTL (*P* ≤ 0.05; FC ± 1.5, Supplementary Table 6). Among the 169 differential phosphopeptides, 149 were dephosphorylated in ALA vs. CTL, whereas 20 were phosphorylated, as shown in the heat map (Fig. 3C) and the Volcano plot (Fig. 3D). The Pearson hierarchical cluster analysis revealed differential expression between two groups among the 169 differential phosphopeptides (Fig. 3E). We conducted a gene ontology (GO) analysis of the significant phosphopeptides to better understand the functional roles of the phosphoproteins found in our dataset (Fig. 3F). The biological processes showed the highest enrichment for “cellular” and the “metabolic process”, “biological regulation”, and the “organic substance metabolic process”. In addition, most proteins were localized in the intracellular and cytoplasm compartments, with molecular functions including “protein binding”, “heterocyclic compound binding”, and “organo cyclic binding” (Fig. 3F).

Proteomic analysis (Fig. 5A) of AT revealed 2,295 proteins, of which 144 were significantly abundant between ALA and CTL (*P* ≤ 0.05; FC ± 1.5, Supplementary Table 7). The PCA of ALA vs. CTL showed a variance of 46% change, and the treatment groups were well clustered, indicating the change in the proteome of ALA vs. the CTL samples (Fig. 5B). Among the 144 differential proteins in ALA, 77 were downregulated, whereas 67 were upregulated, as shown in the heat map (Fig. 5C) and the Volcano plot (Fig. 5D). The Pearson hierarchical cluster analysis indicated a differential expression between two groups among the 144 differentially abundant peptides (Fig. 5E). GO analysis of significant proteins revealed that the biological processes showed the highest enrichment for the “cellular” and “metabolic process”, “biological regulation”, and the “organic substance metabolic process”. In addition, many proteins were localized in the intracellular and cytoplasm compartments, with molecular functions including “ion”, “protein”, and “metal ion binding” (Fig. 5F).

### ALA enriches the phospho-proteome and the proteome of insulin-stimulated AT with insulin signaling and inflammatory pathways and functions

**Figure 4:**
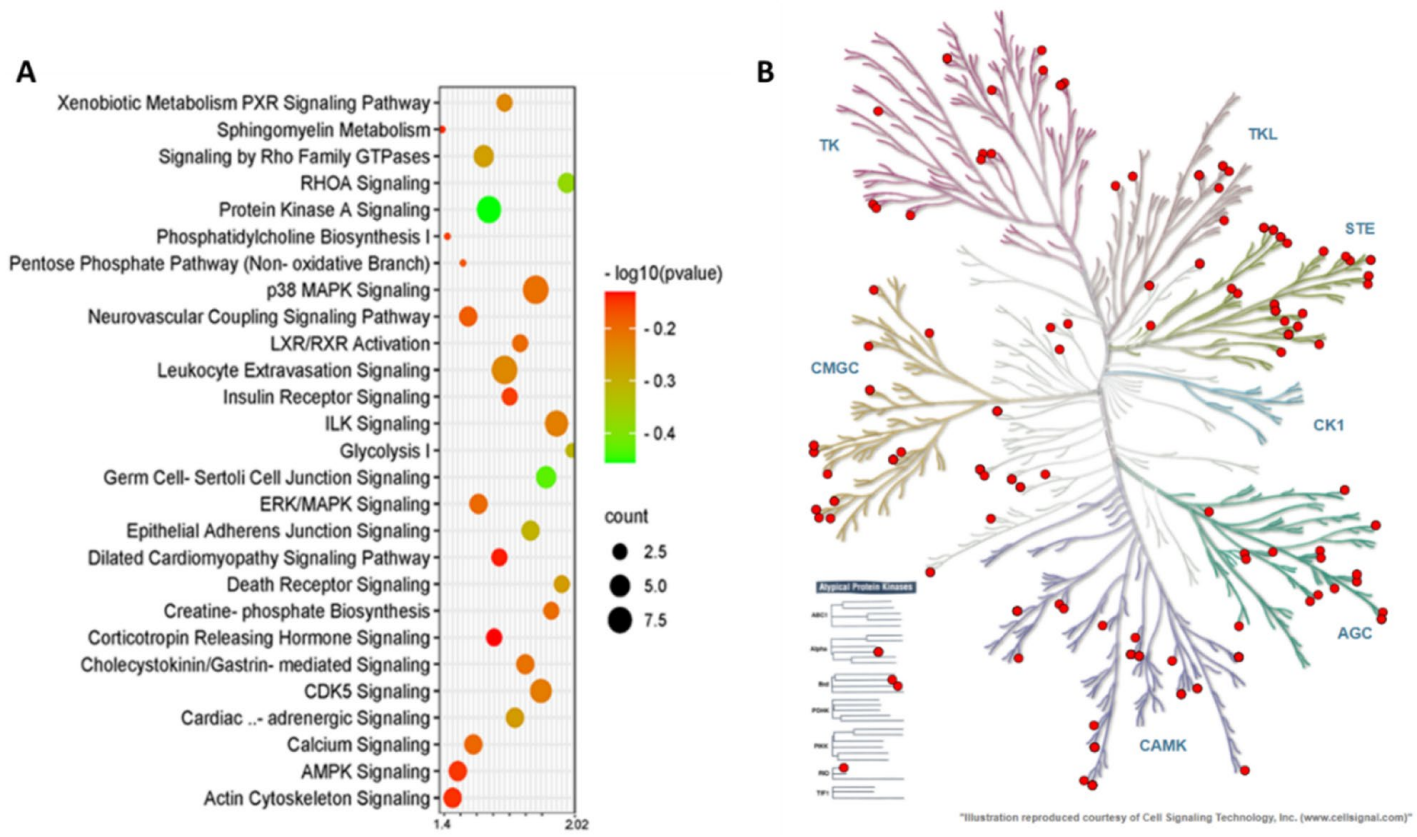
Top canonical pathway analysis and the kinome tree analysis of the adipose tissue phospho-proteome of postpartum (PP) dairy cows supplemented with ALA 20 min after glucose infusion. Dairy cows were divided into two groups from 21 to 60 days PP; (i) CTL—encapsulated saturated fat, (ii) ALA—flaxseed supplement providing α-linolenic acid (ALA). n = 5 per treatment. (**A**) Top canonical pathways associated with ALA supplementation of dairy cows on the Y-axis and—log (P-value) on the X-axis. The size and color of each bubble represent the number of peptides in each pathway and the P value, respectively. (**B**) The predicted kinases of phospho-proteome in ALA-supplemented AT as represented in the kinome tree using the kinmap server. The identified kinases of the Phosphoproteome of dairy cows are represented as follows: *AGC* PKA/PKG/PKC-family kinases, *CAMK* calcium/calmodulin-dependent kinases, *CK1* casein kinases, *CMGC* CDK/MAPK/GSK3/CLK-family kinases, *STE* sterile homologue kinases, *TK* tyrosine kinases, *TKL* tyrosine kinase-like kinases. The kinases are separated and denoted as red circles on the branches of the tree based on their category.

In phosphoproteomics, the top enriched canonical pathways in ALA vs. CTL AT were as follows: Protein Kinase A Signaling, RHOA Signaling, Signaling by Rho Family GTPases, the Xenobiotic Metabolism PXR Signaling Pathway, ILK Signaling, CDK5 Signaling, Cholecystokinin/Gastrin-mediated Signaling, ERK/MAPK Signaling, p38 MAPK Signaling, Calcium Signaling, LXR/RXR Activation, Insulin Receptor Signaling, and the AMPK Signaling pathways (Fig. 4A). The complete list of the top canonical pathways in ALA vs. CTL AT phosphoproteome is provided in Supplementary Table 8. Two signaling pathways associated with insulin signaling were activated: “Protein Kinase A Signaling” and “ERK/MAPK Signaling”. In these pathways, ALA AT showed differential phosphorylation of proteins such as the serine/threonine protein kinases: Adducin1 (ADD1), A-kinase anchoring protein1 (AKAP1), A-kinase anchoring protein12 (AKAP12), FilaminA (FLNA), MHC class I region proline-rich protein CAT53 (PPP1R10), Paxillin (PXN), the Nuclear factor of activated T-cells, and cytoplasmic 1 (NFATC1) in Protein kinase A signaling, whereas NFATC1, PRKAR1A, PXN, and PPP1R10 were phosphorylated in ERK/MAPK Signaling. Furthermore, the activation of “Signaling by Rho Family GTPases” was also detected in the phosphoproteome of ALA AT (Fig. 4).

**Figure 5:**
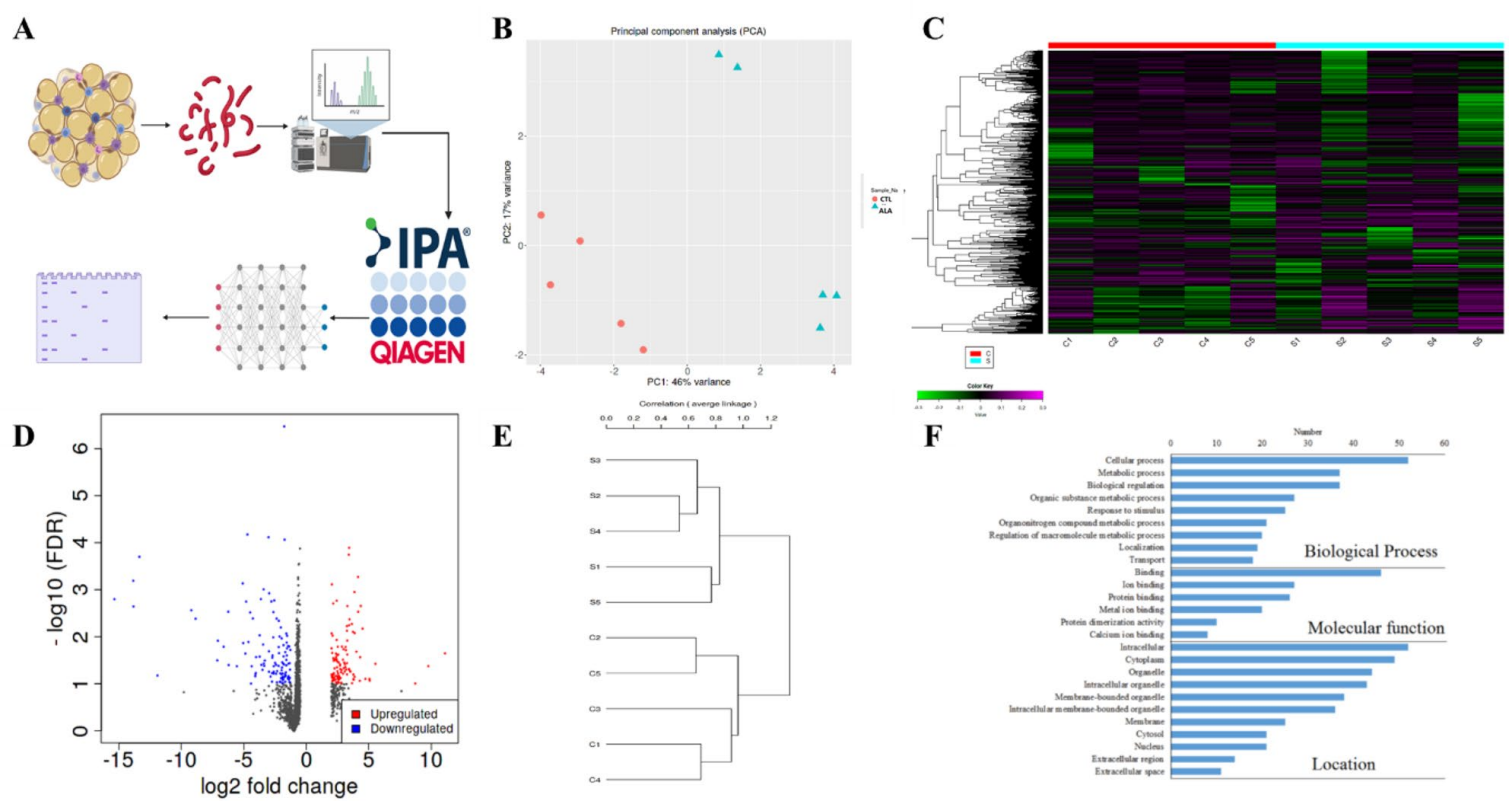
Proteomic analysis of insulin-stimulated adipose tissue from postpartum (PP) dairy cows supplemented with ALA. Dairy cows were divided into two nutritional regiment groups from 21 to 60 days PP; (i) CTL—encapsulated saturated fat, (ii) ALA—flaxseed supplement providing α-linolenic acid (ALA). n = 5 per treatment. (**A**) Work flow for the proteomic analysis; proteins extracted from adipose were digested and peptides were identified for their differential expression. Bioinformatic analysis and network analysis were performed to identify the enriched pathways. Western blot analysis was used for validation. (**B**) Principal component analysis (PCA) of the AT proteome; CTL samples are denoted in red circles, whereas ALA samples are denoted in blue triangles. (**C**) Heat map analysis of AT proteome: low peptide intensity is denoted in green, whereas high intensity is denoted in purple. Each cow in the study is numbered and represented in columns. (**D**) Volcano plot analysis of the AT proteome; the P value (< 0.05) is represented on the Y-axis and FDR (± 1.5) is represented on the X-axis. Each dot represents one protein: red denotes more, whereas blue denotes fewer proteins in AT. (**E**) Correlation of CTL vs ALA cows generated by the IDEP9.5 server where c1, c2, c3, c4, and c5 represent CTL; s1, s2, s3, s4, and s5 represent ALA-supplemented cows. (**F**) GO analysis of AT proteome supplemented with ALA including the biological process category, the molecular function category, and the cell component category. Image was generated using www.Biorender.com.

The top canonical pathways, according to the differential proteome in ALA vs. CTL, were as follows: Oxidative Phosphorylation, Mitochondrial Dysfunction, Acute Phase Response Signaling, LXR/RXR Activation, the NER (Nucleotide Excision Repair) Enhanced Pathway, Clathrin-mediated Endocytosis Signaling, the Sirtuin Signaling Pathway, FXR/RXR Activation, IL-12 Signaling and Production in Macrophages, IL-4 Signaling, the Production of Nitric Oxide and Reactive Oxygen Species in Macrophages, NRF2-mediated Oxidative Stress Response, and the Protein Ubiquitination Pathways (Fig. 5A). Among the pathways (Supplementary Table 9), we focused on the enriched pathways associated with insulin signaling (Fig. 4A). Interestingly, we found that three pathways (Oxidative Phosphorylation, LXR/RXR Activation, and the Production of Nitric Oxide and Reactive Oxygen Species in Macrophages) were inactivated in ALA supplemented AT with a negative Z-score, and no pathways were activated in ALA AT. The Oxidative Phosphorylation pathway included the following differential proteins: ATP5F1A, ATP5MF, COX5B, CYB5A, MT-ATP6, and SDHC. The LXR/RXR Activation pathway included APOM, LBP, ORM1, ITIH4, and RBP4. The Production of Nitric Oxide and the Reactive Oxygen Species in the Macrophage pathways included the following proteins: APOM, PPP2CA, ORM1, and RBP4.

### ALA affects kinase-substrate enrichment and networks in insulin-stimulated AT

**Figure 6:**
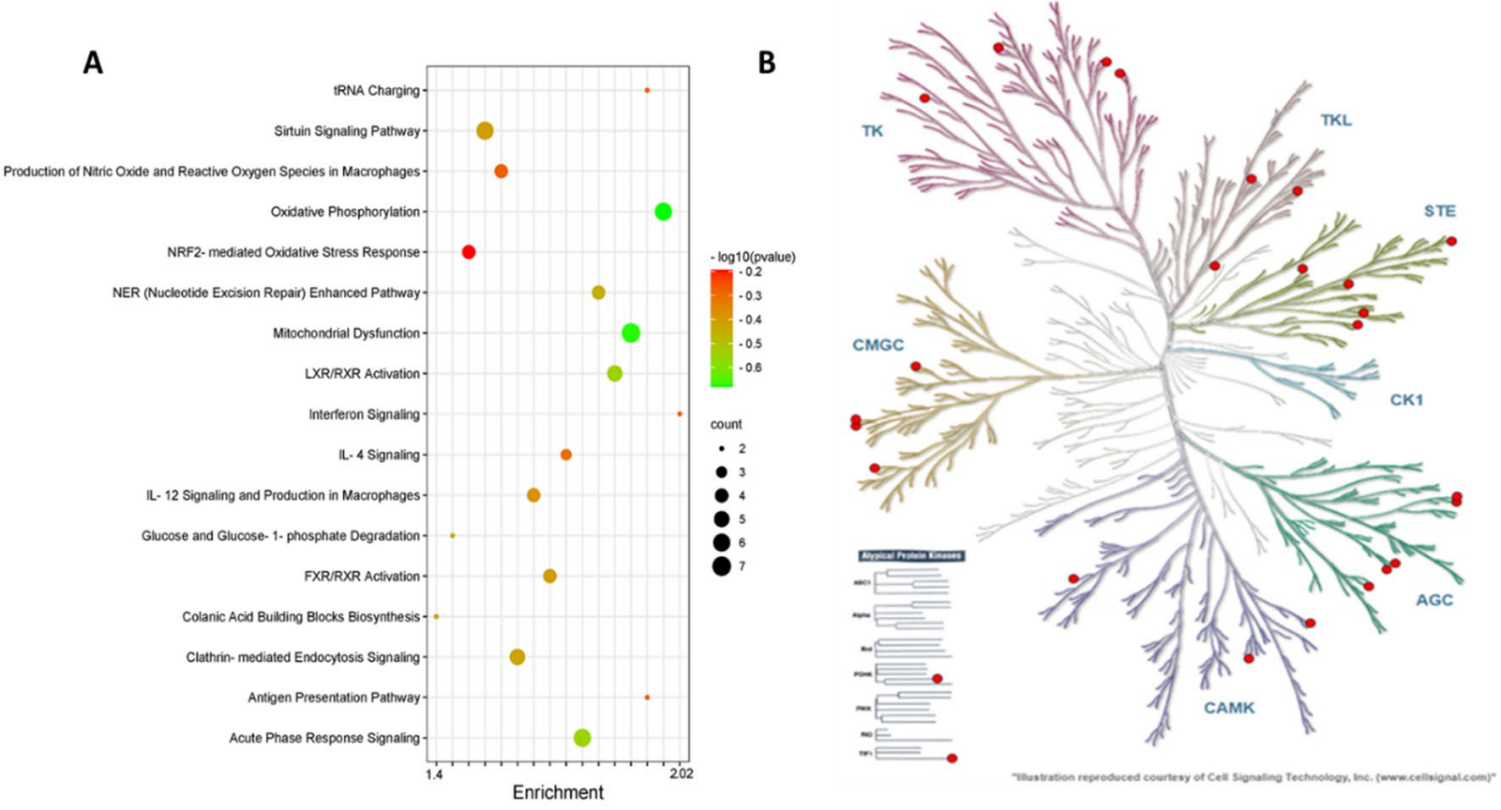
Top canonical pathway analysis and the kinome tree analysis of insulin-stimulated adipose tissue proteome from postpartum (PP) dairy cows supplemented with ALA. Dairy cows were fed; (i) CTL—encapsulated saturated fat, (ii) ALA—flaxseed supplement providing α-linolenic acid (ALA). n = 5 per treatment. (**A**) Top canonical pathways according to differential proteins in ALA vs. CTL (Y-axis), and—log (*P*-value) on the X-axis. The size and color of each bubble represent the number of proteins enriched in each pathway and the P value, respectively. (**B**) The predicted kinases of proteome in ALA-supplemented AT represented in the kinome tree using the kinmap server. The identified kinases in the proteome of AT are represented as follows: *AGC* PKA/PKG/PKC-family kinases, *CAMK* calcium/calmodulin-dependent kinases, *CK1* casein kinases, *CMGC* CDK/MAPK/GSK3/CLK-family kinases, *STE* sterile homologue kinases, *TK* tyrosine kinases, *TKL* tyrosine kinase-like kinases. The kinases are separated and denoted as red circles on the branches of the tree, based on their category.

To study the kinase activity based on phosphorylation changes, we analyzed the differential phosphorylated sites by Kinemap, and found kinases that are influenced by ALA supplementation. Among the phospho-serine, threonine, and tyrosine sites, Tyrosine kinase phosphorylation was found to be more frequent on protein kinases compared with other identified proteins. The phosphoproteomic data were used to classify kinase activity in ALA AT and to identify kinases that control the insulin-signaling pathway (Figs. 4B, 6B).

**Figure 7:**
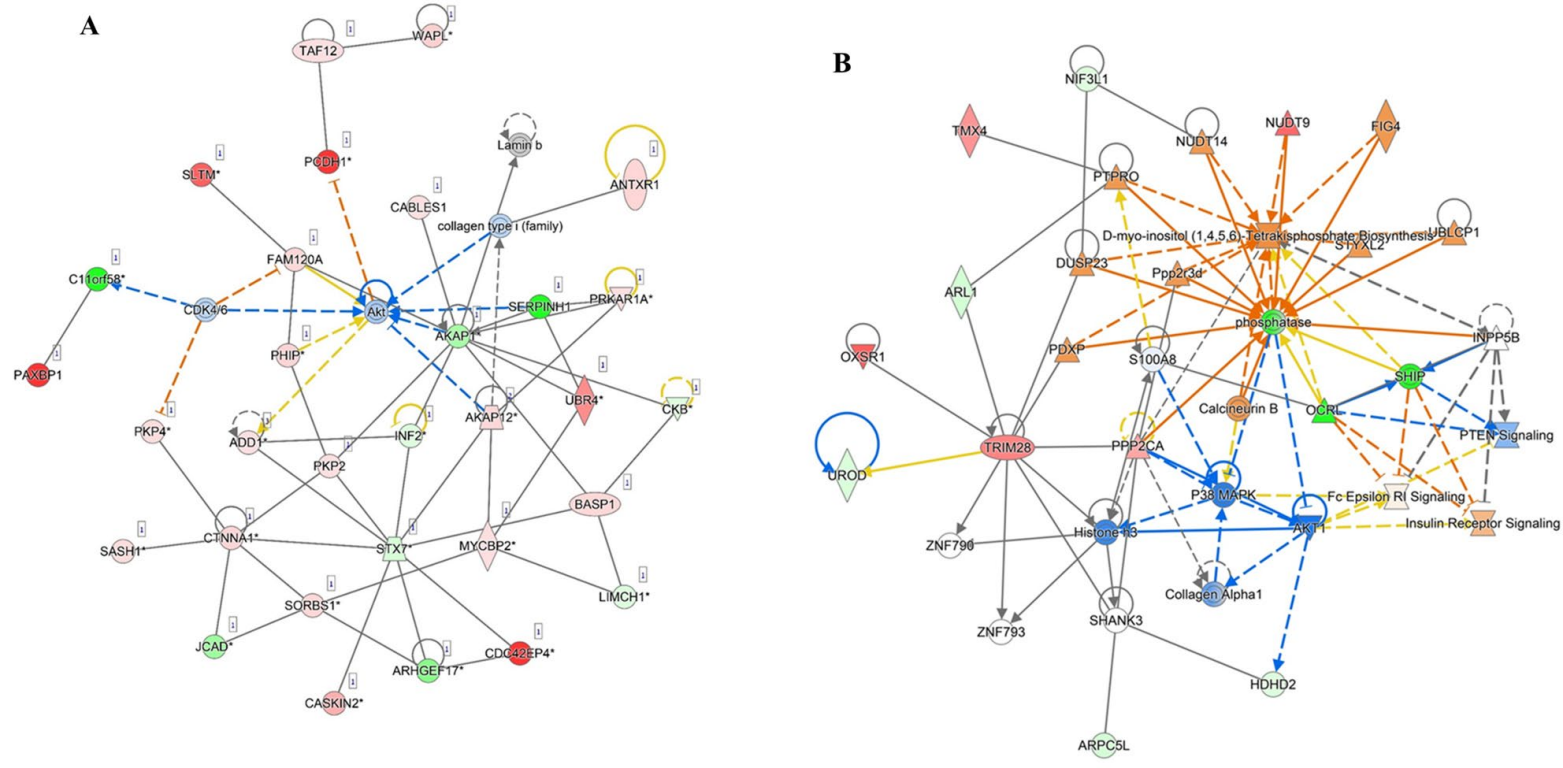
Network analysis of phosphoproteomics (**A**) and proteomics (**B**) of insulin-stimulated adipose tissue reveals the main role of Akt signaling following peripartum dietary ALA supplementation in postpartum dairy cows. Dairy cows were fed; (i) CTL—encapsulated saturated fat, (ii) ALA—flaxseed supplement providing α-linolenic acid (ALA). n = 5 per treatment. The upregulated phosphopeptides or proteins are represented in red, whereas green indicates downregulation. Full lines represent direct interaction, whereas dotted lines represent indirect interaction.

Both in phosphoproteome and in proteome analysis, one of the top networks that were differentially enriched in ALA vs. CTL AT and generated under ‘Carbohydrate and lipid metabolism’ captured 19 and 13 proteins, respectively, with MAPK, ERK1/2, PI3K, and Akt as the main effectors (Fig. 7A,B). According to the networks, in the insulin-stimulated AT of ALA-supplemented cows, AKT1 directly acts on insulin and insulin receptor signaling and interdependently on MAPK, which in turn, again acts on ERK1/2 and PI3K.

### ALA decreases plasma and adipose endocannabinoids during GTT

**Figure 8:**
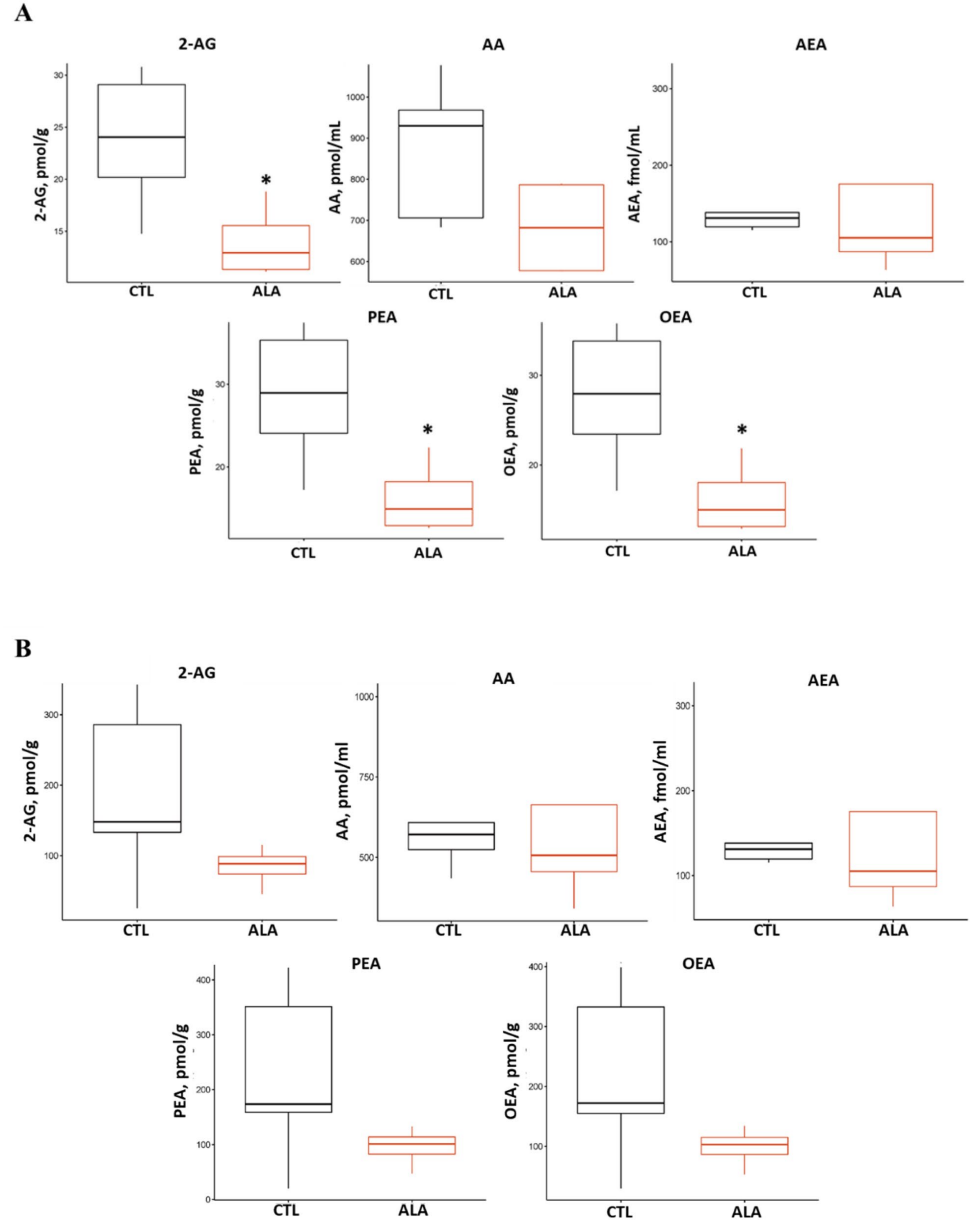
Endocannabinoid concentrations in plasma (**A**) and adipose tissue (**B**) at 20 min after glucose infusion from postpartum (PP) dairy cows supplemented with ALA. *P ≤ 0.05. Dairy cows were divided into two nutritional groups from 21 to 60 days PP; (i) CTL—encapsulated saturated fat, (ii) ALA—flaxseed supplement providing α-linolenic acid (ALA). n = 5 per treatment. *2-AG* 2-arachidonoylglycerol, *AA* arachidonic acid, *AEA* anandamide, *PEA N*-palmitoylethanolamide, *OEA N*-oleoylethanolamide.

As shown in Fig. 8A, the average plasma concentrations of 2-arachydonoylglycerol (2-AG; *P* = 0.03), *N*-palmitoylethanolamide (PEA; *P* = 0.03), and *N*-oleoylethanolamide (OEA; *P* = 0.03) were ~ twofold lower in ALA than in CTL, whereas AA tended to be lower in ALA (*P* = 0.10) at 20 min post-glucose infusion. In AT, the average concentrations of 2-AG, *N*-arachidonoylethanolamide (Anandamide; AEA), PEA, and OEA were numerically but not significantly lower in ALA than in CTL (*P* = 0.16, Fig. 8B). In addition, the relative levels of the eCB precursors 2-Dihomo-gamma-Lineoyl Glycerol (2-HG, *P* = 0.03) and *N*-arachidonoyl serine (ARA-S, *P* = 0.05) were lower in plasma of ALA compared with CTL (Supplementary Fig. 2).

### ALA lowers the protein levels of inflammatory and ECS components in AT

The levels of TNFα (*P* = 0.03) and the p65 subunit of the transcription factor NF-κB (RELA, *P* < 0.01) were lower in ALA than in CTL AT (Supplementary Table 10). The average protein abundance of MGLL was twofold lower (*P* = 0.05), whereas fatty acid amide hydrolase (FAAH; *P* = 0.11) and AMP-activated protein kinase (AMPK, *P* = 0.06) tended to be lower in the AT of ALA than in CTL (Supplementary Table 10). No differences were found in the AT protein abundance of CB1, CB2, and diacylglycerol lipase-a (DAGLA) between groups (Supplementary Table 10).

## Discussion

In the present study, we demonstrated that peripartum dairy cows fed with n-3 supplement rich in ALA had a lower n-6/n-3 ratio in blood, coinciding with a reduction in immune response, lower ECS components, and increased insulin sensitivity. Stimulating AT with glucose showed a strong dietary group clustering in phosphoproteomic and proteomic data, which were used to identify proteomic networks supporting the lower insulin sensitivity observed in ALA AT, in addition to fewer inflammatory proteins in ALA AT. The systemic- and AT-specific alterations in insulin sensitivity and inflammation PP could be associated with the modulation of ECS activation by n-3 supplementation.

Peripartum supplementation with ALA lowered n-6/n-3 levels in plasma, reduced feed intake PP, and had little effect on the concentrations of blood metabolites and hormones. These results are similar to our previous studies in which peripartum ALA supplementation decreased the n-6/n-3 ratio in plasma and in PBMC, and reduced feed intake PP without affecting the blood metabolic indices^26^. However, other studies in which ALA was supplemented to peripartum cows showed increased intake^31^, whereas others observed no effect on feed intake^32–34^, or reduced intake^35^; the possible causes for these inconsistencies were previously discussed^36^. It should be noted that the supplement of extruded flaxseed is not completely protected in the rumen, and therefore some of the FA maybe partly bio-hydrogenated, which can depress the feed intake. Alternatively, in mammals, activation of CB1 in the brain increases feed intake^37^, and in mice and obese humans it was demonstrated that blocking CB1 or a genetic knockout resulted in decreased feed intake^38,39^. In this study we found lower plasma levels of the following eCBs: 2-AG, PEA, and OEA; and AA tended to be lower in ALA vs. CTL. We also found lower levels of the eCB precursors 2-HG and ARA-S in plasma, as well as reduced protein abundances of CB1 and MGLL in the PBMC of ALA compared with CTL. Hence, the reduction in feed intake in ALA cows may be also associated with the lower ECS activation, which is in line with our previously proposed premise^26^.

We found that peripartum supplementation with ALA was associated with lower levels of systemic pro-inflammatory cytokines (IL-6 and IL17-α), a lower percentage of WBC, and decreased TNFα in PBMC PP. In addition, we found a reduction in the percentage of natural killer T cells prepartum in ALA vs. CTL. These findings support our previous report on the anti-inflammatory effects of ALA on PP cows, and they are in agreement with an in-vitro report in which mononuclear cells from peripartum cows were cultured with ALA^40^. Omega-3 FA can directly improve immune function, since they exert anti-inflammatory effects, which include producing prostaglandins, inhibiting inflammatory factor NF-κB, a decreased production of inflammatory cytokines, and synthesis of inflammatory resolution mediators such as resolvin and other oxylipids^22–24,40^. Nonetheless, n-3 supplementation, and specifically high levels of ALA supplementation can also affect immune function via alterations in ECS activation^1^. In this study, we found lower plasma concentrations of 2-AG and a tendency for lower AA levels in ALA cows PP compared with CTL cows. 2-AG and its precursor, as well as degrading product, AA may have pro-inflammatory properties^41^, since 2-AG contributes to the recruitment of leukocytes^42–44^. Moreover, CB1 can be upregulated by inflammatory cytokines in the PBMC of autoimmune-diseased humans^45^. Thus, our finding of decreased CB1 in the PBMC of ALA cows PP and lower levels of eCBs in their blood could be related to the anti-inflammatory effect of ALA, suggesting that ECS is associated with peripartum immune function.

A reduction in the protein levels of TNFα and of RELA, one of the structural regulators of NF-kB, was found in the AT of ALA vs. CTL. The AT proteomic analysis revealed several inflammatory pathways enriched in ALA: interferon signaling, IL-4 signaling, and IL-12 signaling, and the production of macrophages, as well as the acute phase response-signaling pathway (Fig. 6). Recently, we have demonstrated by proteomic analysis of PBMC lower levels of acute phase response signaling proteins in cows supplemented peripartum with ALA. Taken together, these effects of ALA on inflammatory pathways in AT and PBMC are indicative of tissue-specific anti-inflammatory effects. Interestingly, these changes in inflammatory proteins in AT were coupled with lower levels of MGLL and a tendency for lower levels of FAAH in ALA AT. The levels of AT eCBs were only numerically, but not significantly, lower in ALA than in CTL; nonetheless, this trend may indicate that ALA supplementation reduces ECS activation in the AT of ALA PP, which supports our previous findings in which peripartum ALA reduced PEA levels in AT, but not in other eCBs^26^. Increased AT inflammation was found in cows that had a higher degree of AT lipolysis PP, coupled with a higher gene expression of the CB2 receptor^46^; we previously reported that 2-AG and AEA levels were higher in the PP AT of cows with higher inflammation in AT^47^. In the present study, we found that ALA decreased inflammation and affected some ECS components in AT; therefore, along with the findings of others^46,47^, it is reasonable to propose that an association exists between AT inflammation and ECS activation in PP dairy cows.

ALA supplementation decreased insulin concentrations and AUC while maintaining similar glucose clearance during GTT, which indicates higher systemic insulin sensitivity compared with that of CTL. Dietary supplementation of n-3 FA has been shown to improve insulin sensitivity in animal and human models^48–50^; in mice, a high-fat diet containing ALA increased insulin sensitivity, as manifested by a higher phosphorylation of Akt and glycogen synthase kinase-3 beta (GSK3β) in the liver^48^. In humans with metabolic disorders, short-term n-3 supplementation increased insulin sensitivity^49^, and in insulin-resistant mares, ALA supplementation increased insulin sensitivity under GTT^50^. Taken together, these reports support our findings of increased systemic insulin sensitivity in cows supplemented with ALA. We found that ALA supplementation lowered the plasma concentrations of PEA and OEA during GTT compared with CTL; however, studies in humans and rodents indicate that PEA and OEA are associated with increased insulin sensitivity^51,52^. This discrepancy can be attributed to the possible difference between healthy PP animals and diseased models.

In the present study, AT biopsies were collected 20 min after glucose infusion, at peak insulin levels as previously described^53^; in-vitro studies using mouse adipocytes indicated that approximately 20% of their phosphoproteome is insulin regulated within 20 min post-stimulation^19,54^, thus validating our experimental model. In the glucose-stimulated AT, we found lower levels of the insulin receptor, reduced phosphorylation of Akt, and clear clustering in the phosphoproteomic data of ALA vs. CTL AT, which indicated several differential pathways related to insulin signaling, such as PKA signaling, insulin receptor signaling, and ERK/MAPK signaling. Taken together, these findings suggest lower insulin signaling in the AT of ALA compared with CTL. However, since the ALA cows secreted lower levels of insulin in response to glucose infusion, we can assume that the AT was exposed to less insulin; thus, the lower signaling may be a dose response, and not necessarily indicative of lower sensitivity. Nonetheless, the clear clustering and vast changes in protein phosphorylation and in the ALA AT levels exhibited in phosphoproteomics, proteomics, and immunoblots support a decrease in insulin sensitivity in AT, which is contrary to the systemic effect of increased insulin sensitivity observed in ALA vs. CTL cows. Indeed, impaired Akt phosphorylation in AT is observed in cases of insulin resistance where the insulin-stimulated glucose transport is decreased^55^. We propose that the lower insulin sensitivity in AT could be a counter-balancing response to the systemic increase in insulin sensitivity observed PP in ALA-supplemented cows.

In our study we found that ALA AT had lower levels of pro-inflammatory proteins (TNFα and RELA) along with lower insulin sensitivity. In humans, it was shown that obesity-induced low insulin sensitivity correlates with higher pro-inflammatory cytokines^56^, which contrasts with our findings. Moreover, n-3 FAs have been linked to higher insulin sensitivity in AT of obese mice, along with reduced inflammation, lipogenesis, and elevated markers of fatty acid oxidation in cultured adipocytes^3^. Therefore, we can postulate that the etiology of obesity in humans greatly differs from the physiological response of AT in healthy PP cows that experience lipolysis rather than lipid accumulation; thus, they are different physiological states. Nevertheless, TNFα has been shown to induce phosphorylation of the insulin receptor in murine adipocytes^57^, which supports our finding of reduced TNFα protein levels and lower insulin sensitivity in ALA AT.

Our phosphoproteomic analysis of AT revealed that ALA AT increased the phosphorylation of AMPK signaling, along with a tendency of reduced AMPK levels in the AT. We suggest that lower PEA levels in AT may be associated with the decrease in insulin signaling in ALA AT. Moreover, we found that ARA-S was lower in the plasma of ALA compared with CTL. ARA-S affects the activation of the ERK1/2, PI3K, and Akt in human endothelial cells^58^. This is in accordance with our findings of lower ARA-S levels in ALA cows, as well as the differential enrichment of the ERK1/2/AMPK signaling, and PKA pathways, as shown by the phospho-proteomic results discussed next.

Dysregulation of intracellular signaling by altered phosphorylation is a common underlying mechanism of insulin resistance^59^. In our previous work, we demonstrated that cows that had a high degree of BW loss PP had diminished phosphorylation of Akt in their AT following GTT, supporting this premise^53^. Our phospho-proteomic analysis of insulin-stimulated AT provides new insights into the molecular pathways that were altered by ALA supplementation in PP cows. The insulin receptor, insulin receptor substrates 1 and 2, Akt, and mTORC1 are important components in the insulin signaling cascade that are either kinase or phosphorylation-dependently activated when stimulated with insulin^60^. Several quantitative mass spectrometric studies in mice and in in-vitro cells have studied^13,61–64^ the phospho-proteomic aspects of the PI3K/Akt/mTORC1 pathway, but no data exist on insulin sensitivity based on the phosphoproteomics of AT in livestock.

In the present study, we found that differential phosphopeptides in ALA AT were associated with enriched AGC kinases (a subfamily of protein kinases that include protein kinase A; Fig. 4). The stimulation of downstream kinases by insulin results in the activation of many of kinases that have identical substrate specificities. Both Akt and ERK, which are AGC kinases, can preferentially phosphorylate phospho-Akt Substrate Motif (RxRxxS/T motifs)^65^. We found increased phosphorylation of protein kinase cAMP-dependent type-I regulatory subunit alpha (PRKAR1A), along with decreased Akt signaling in ALA AT. In mice, insufficient protein levels of PRKAR1A are associated with malfunction and over-activation of the Akt signaling pathway^66^. The upregulation of this pathway may be a counter-balancing mechanism in AT to account for changes in insulin sensitivity. Another enriched pathway was RhoA signaling, which was decreased in ALA compared with CTL AT. RhoA is one of the Rho family proteins (RhoA, RhoB, and RhoC), and is a part of the larger Ras superfamily of guanosine triphosphatase hydrolase enzymes (GTPases)^67^. This protein seems to act primarily in migrating cells by regulating vascular contraction and is activated by immune signals^67^; thus, the lower activation of this pathway in ALA AT may be associated with the decreased inflammation that we observed in AT. We also observed enrichment of the peroxisome-proliferator-activated receptor alpha (PPARα) signaling pathway, along with a numerical trend of decreased AT levels of OEA and PEA, which are known to activate PPARα^68^. Most studies on the activation of PPARα in rodents suggest improvement of insulin sensitivity and signaling, due to decreased ectopic lipids in non-ATs and decreased circulating FA and TG^69^. However, no differences in plasma NEFA or TG levels PP were observed between groups. Nevertheless, the LXR/RXR pathways, which are co-factors of the PPARα, were enriched according to the differential proteins in ALA AT (Fig. 4). This may be another counter-balancing mechanism in the AT to regain insulin sensitivity. Taken together, the phosphoproteomics of insulin-stimulated AT provides insights into the molecular pathways that are altered by ALA supplementation in PP animals, and it supports the tissue-specific effect of ALA on insulin signaling in AT compared to the systemic response. The strengths of the present study lie in the complex in-vivo setting that involves multiple procedures and the collection of blood and tissues from PP cows, the vast array of metabolic, immune, and ECS-related analyses in blood and tissues, along with the cutting-edge phosphoproteomics and proteomics of AT following an in-vivo GTT. The main limitation of our study is the relatively low number of cows that could participate in such an intensive and complex experiment.

Our phospho-proteomic and proteomic data of glucose-stimulated AT, combined with functional systemic and AT-specific data, support a model whereby dietary n-3 supplementation rich in ALA during the peripartum period increases systemic insulin sensitivity, while decreasing insulin sensitivity in AT PP. The ALA supplementation exerted anti-inflammatory effects and lowered ECS components, both systematically and in AT. Omega-3 FA and the ECS both regulate immune function; thus, we suggest that the immunometabolic effects of ALA supplementation may also be mediated via the ECS in PP dairy cows.

## Materials and methods

### Animals and experimental procedures

The Volcani Center Animal Care Committee approved the study’s experimental methodology, according to the necessary regulations and guidelines (approval number IL 882/20). This study was carried out in compliance with the ARRIVE guidelines. The experiment included 32 multiparous Israeli-Holstein dairy cows at 256 days of gestation (mean parity 3.2 ± 1.32; mean SD) at the Volcani Center experimental farm in Rishon Lezion, Israel. The cows were housed collectively in a shaded loose pen with an electronic individual feeding system that operated in real time. The cows were divided into groups based on their parity, body weight (BW) upon drying off, and milk production during the first 60 days of the previous lactation. The dietary interventions started at day 256 of pregnancy and continued through day 60 of lactation: (i) CTL cows (*n* = 16) were fed a basal diet supplemented with 250 g/d/cow calcium salt of fatty acids (Adolac, Poliva, Israel) and PP supplemented at 1.6% of the diet (DM basis); (ii) ALA cows (*n* = 16) were fed a basal diet and supplemented prepartum with 700 g/d/cow of extruded flaxseed supplement rich in ALA C18:3n-3 (Valomega 160, Valorex, France), and PP supplemented at 6.4% of diet (dry matter basis). The diets were balanced for energy, protein, and ether extract content. In Supplementary Table 1, the main FAs in the diets are profiled along with the ingredients and the chemical composition of the rations PP. Body weight (BW) and milk output were measured three times each day following calving (SAE, Kibbutz Afikim, Israel). Every 2 weeks, milk samples were taken and examined in the labs of the Israeli Cattle Breeders’ Association for milk fat, protein, lactose, and urea using infrared analysis in accordance with standard IDF 141C:2000 (Caesarea, Israel). Energy balance was calculated according to NRC equations (2001). According to standard cow management in Israel, cows are inspected by a veterinarian five to ten days after calving. The body condition score (BCS; scale 1–5) was examined weekly by a single technician, as described previously^70^. Blood samples were collected twice a week at 0700 h, centrifuged at 4000×*g* for 15 min and kept at − 80 $^\circ$C. An additional blood sample was collected at d 10 PP for separation of peripheral blood mononuclear cells (PBMCs). Blood samples were also collected once a week from 2 weeks prepartum to 2 weeks PP for flow cytometry and complete blood count (CBC). When we were running the study, we performed the invasive procedures on subgroups of randomly selected cows from each group as was planned. However, post factum we decided to remove five cows from all analyses due to clinical ketosis diagnosed at 8–10 d PP.

### Fatty acid composition and analysis of metabolites and inflammatory markers in blood plasma

Fatty acid (FA) composition in the plasma (n = 12) was examined at 14 d PP by gas chromatography as described previously^71^. Metabolic and immune indices in blood were examined in all cows PP; plasma non-esterified fatty acid (NEFA) concentrations were determined PP using the NEFA C Test Kit (Wako Chemicals GmbH, Neuss, Germany); insulin concentrations were determined by RIA (Diagnostic Products, Los Angeles, CA), and β-hydroxybutyric acid (BHBA) concentrations were determined using a Ranbut D-3-Hydroxybutyrate Kit (Randox, Crumlin, UK)^72^. Concentrations of glucose, triglycerides, and aspartate aminotransferase (AST) activity were analyzed using a Cobas C111 analyzer (Roche Holding GmbH, Grenzach-Wyhlen, Germany). Cortisol concentrations were determined by ELISA (EIA1887, DRG International, Inc., Springfield, NJ, USA). Plasma concentrations of 15 cytokines and chemokines (Interleukins 6, 17α, 1α, 1β, 1RA, 2, 8, 10, and 4, interferon γ, TNFα, as well as chemokines 2, 3, and 4) were determined at week 1 PP at the laboratory of Dr. Gilles Foucras (UMR IHAP, ENVT, Veterinary school at Toulouse, France) using a custom bovine cytokine/chemokine bead-based multiplex assay (Merck Millipore) as described^73^.

### WBC subpopulations, complete blood counts, and PBMC separation from blood

The percentages of white blood cell (WBC) subpopulations by flow cytometry^74^ and CBC^75^ were examined at 4 time points (− 14 ± 1.6, − 7 ± 0.5, + 7 ± 0.6, and + 14 ± 0.2 days relative to parturition) in 12 cows per treatment. Peripheral blood mononuclear cells (PBMCs) were extracted from blood at 10 ± 0.1 d PP from 8 CTL and 6 ALA randomly selected cows as described^75^.

### Glucose tolerance test (GTT) and adipose tissue biopsies

To examine the effect of ALA supplementation on insulin sensitivity, an intravenous glucose tolerance test (GTT) was conducted at 5–8 d PP on 7 cows per treatment. Two ALA cows were excluded from analysis because they were diagnosed with ketosis at d 8–10 PP (after GTT). A GTT was performed after the morning milking without access to feed as previously described^53^. Blood samples were collected 5 min before infusion, at time 0 (infusion), then every 5 min until 30 min post-infusion, and at 40 and 60 min post-infusion. Blood tubes were immediately placed on ice, centrifuged within 30 min of collection at 4000×*g* and stored at − 80 °C, pending analysis of glucose and insulin.

Subcutaneous AT biopsies were conducted at 20 min after glucose infusion^53^ from the fat pad around the pin bones as described previously^53^. From each cow, four samples of ~ 40 mg of AT were snap frozen and stored at − 80 °C.

### Sample preparation for proteomic and phospho-proteomic analysis of AT

Adipose samples from 5 cows per treatment were homogenized in 1 mL lysis buffer containing 5% SDS in Tris-HCl, and protease and phosphatase inhibitors. Following centrifugation for 15 min at 20,000×*g* at 4 °C and after debris extraction, the protein concentration was determined by bicinchoninic acid assay (BCA; 9470BCA stand, Cyanagen, Bologna, Italy) and samples were snap-frozen and stored at − 80 °C. For phosphoproteomics, protein lysates were isolated for global phospho-proteome quantification by Fe immobilized metal ion affinity chromatography (Fe-IMAC) with enrichment of phospho-peptides, followed by discovery analysis.

### Sample preparation for LC MS/MS

Using the S-trap approach, the proteome and phospho-proteome samples were lysed and digested using trypsin. Nanoflow liquid chromatography (nanoAcquity) and high-resolution, high-mass accuracy mass spectrometry were used to evaluate the resulting peptides (Q Exactive HFX). In a discovery mode, each of the samples was analyzed separately using the instrument in a random order.

### Data processing and analysis

Using intensity-based label-free proteomics, the raw data were processed and (phospho) proteins were quantified. Raw data were imported using Maxquant v2.0.1. The data were compared to the bovine proteome database, which was supplemented with common lab protein contamination, using the Andromeda search engine. The LFQ approach was used for quantification, based on unique/all peptides. In at least one group, data were screened for replication in at least four out of 6 replicates. Then, using peak volume at retention time, m/z, and intensity space, we carried out isotopic clustering and feature identification. The results were compared to the UniprotKB (http://www.uniprot.org) *Bos taurus* sequences, which were augmented with common laboratory-contaminating proteins. Cysteine carbamidomethylation was set as the fixed modification, whereas methionine oxidation was specified as the variable modification. An in-house script51 was used to group (phospho) proteins and quantify them. To standardize the data, the total ion current was employed.

### Hierarchical clustering of phosphor and proteomic AT samples

Based on the relative abundances of the detected differentially expressed (phospho) proteins in each sample, unsupervised hierarchical clustering of the individual samples and the proteins was performed using the IDEP9.1 server tool, using average linkage clustering, and the Kendall’s tau distance calculation process. The peptide expression was displayed using a heat map and PCA, with dendrograms indicating the clustering results. The fold change was processed using the log2 function to center the data on zero, whereas the Benjamini-Hochberg-corrected P value was − log10 transformed for volcano plot scaling.

### Gene Ontology (GO) analysis of phospho and proteomic AT samples

The significantly altered (phospho) peptides with up- and down-regulated sites on ALA adipose were grouped based on the GO Biological Process and Molecular Structure using the BINGO functional annotation approach in Cytoscape. A minimum of three representative proteins in each class were required for the GO keyword filter to identify significantly enriched groups with a P value of 0.005 for the control and ALA. The filtered P values are shown as bar graphs based on the KEGG pathways, the molecular function, and GO keywords. The route annotation was performed using the Foam tree for lipid metabolism and the Reactome pathway database for metabolic enzymes.

### Kinase-substrate prediction

A kinmap was used to predict probable kinase-substrate interactions and consequently kinase activity. The serine-threonine kinases were identified with a false positive rate (FPR) of 10% using the upregulated and downregulated peptides that were saved in (Phos) PEP format. Only kinases with a P value of 0.05 were deemed significantly altered. Based on the average abundances (log2-fold changes) of phosphorylated substrates for kinases that were thought to be relevant, further kinase-substrate enrichment analysis was carried out.

### Network and pathway analysis of phosphopeptides

The interactions of (phospho) proteins were examined using the STRING database and GeneGO MetaCore. For the metabolic network mapping process, the Kyoto Encyclopedia of Genes and Genomes (KEGG) was used to collect the pathways associated with each protein. The most relevant pathways for phosphoproteins and proteins that were differentially abundant at P ≤ 0.05 and FC ± 1.5 were determined using Qiagen Ingenuity Pathway Analysis (IPA, Qiagen Redwood City, CA). Upstream regulators, canonical pathways, and functional regulatory networks were identified using prediction algorithms and the hypergeometric distribution method. P ≤ 0.05 and P ≤ 0.01, respectively, were chosen as the significant levels for route and network analysis.

### Measurements of endocannabinoids in plasma and adipose tissue

The concentrations of the following endocannabinoids (eCBs): 2-arachydonoylglycerol (2-AG), arachidonic acid (AA), *N*-arachidonoylethanolamide (Anandamide; AEA), *N*-palmitoylethanolamide (PEA), and *N*-oleoylethanolamide (OEA) were measured by using a stable isotope dilution of LC–MS/MS as reported previously^76^ in plasma and in AT 20 min after glucose infusion (n = 5). The plasma values are shown in pmol/mL and fmol/mL, and the AT values are shown in fmol/mg tissue (wet weight) or pmol/mg tissue (wet weight). In addition, we examined the relative quantification of a panel of eCBs and sphingolipids on the same plasma samples according to Malitsky et al.^77^ but with some modifications, by UPLC-ESI–MS/MS equipped with an Acquity UPLC I class system (Waters Corp., MA, USA). An MS detector (Waters Xevo TQ-XS) was equipped with an ESI source. The measurement was performed in positive ionization mode.

### Immunoblot analysis in PBMC and adipose tissue

Protein extractions from PBMC and AT were performed as described previously^74,78^. The AT lysates (n = 7) were loaded at 35 µg/well, whereas the PBMCs were loaded at 35 µL/well (n = 7). The list of antibodies utilized, their dilutions, and their sources are all listed in Supplementary Table 2. The beta-actin protein abundance (1:1000, ab46805, Rabbit Bovine, Abcam Biotech, Cambridge, UK) was used to normalize all samples. Goat anti-rabbit HRP-conjugated secondary antibody (Jackson Immunoresearch; 111-035-003, PA, USA), at a concentration of 1:10,000, was used. It was developed by an ECL reaction for protein detection (Thermo Fisher Scientific, Waltham, MA, USA). The chemiluminescence signals were measured for at least five successive exposure times to establish the linear range of the signal intensity for each antibody. ImageJ software was used to analyze data through densitometry (NIH, Bethesda, MD). Two ALA AT samples were removed from the analysis: one due to low protein levels in the lysate, and the other due to diagnosis of ketosis. The abundance of IRβ was examined in a subset of 4 AT per treatment due to the limited amount of lysate available for analysis.

### Statistical analysis

All continuous variables: feed intake, milk yields, milk components, the calculated energy balance, blood metabolites and hormones, the subpopulations of white blood cells, and CBC were analyzed by PROC MIXED using the following model:

$${\text{Y}}_{{{\text{ijkl}}}} = \, \mu \, + {\text{ T}}_{{\text{i}}} + {\text{ C}}\left( {\text{T}} \right)_{{{\text{ij}}}} + {\text{DIM}}_{{{\text{ijk}}}} + E_{{{\text{ijkl}}}} ,$$

where µ = the overall mean, T_i_ = the treatment effect (_i_ = CTL or ALA), C(T) _ij_ = cow j _nested_ in treatment i, DIM_ijk_ = days in milk as a continuous variable, and *E*_ijkl_ = a random residual.

The FA composition in plasma, weekly BW and BCS, protein abundances, and plasma eCBs were analyzed by SAS GLM (version 9.2, 2002). The pAkt/gAkt values in AT were not normally distributed and therefore were log transformed for statistical analysis. The phosphoproteomic and proteomic data were analyzed by unpaired two-tailed Student’s t-test after log 2 transformation (P ≤ 0.05).

## Supplementary Information

Supplementary Information 1.

Supplementary Information 2.

Supplementary Information 3.

Supplementary Information 4.

## Data Availability

The data that support the findings of this study are available in the “Methods” section and/or the Supplementary Materials of this article.

## Abbreviations

ALA: α-Linolenic acid
AKT: Protein kinase B
PI3K: Phosphatidylinositol-3-kinase
GLUT4: Glucose transporter type 4
FA: Fatty acid
n-3: Omega-3
n-6: Omega-6
ECS: Endocannabinoid system
eCBs: Endocannabinoids
2-AG: 2-Arachidonoylglycerol
AEA: Anandamide or *N*-arachidonoylethanolamide
AA: Arachidonic acid
FAAH: Fatty acid amide hydrolase
DAGLA: Diacylglycerol lipase alpha
MGLL: Monoacylglycerol lipase
CB1: Cannabinoid-1 receptor
CB2: Cannabinoid-2 receptor
PP: Postpartum
GTT: Glucose tolerance test
BW: Body weight
BCS: Body condition score
CBC: Complete blood count
BHBA: β-Hydroxybutyric acid
NEFAs: Non-esterified fatty acids
TG: Triglycerides
AT: Adipose tissue
WBC: White blood cells
PBMC: Peripheral blood mononuclear cells
OEA: *N*-Oleoylethanolamide
PEA: *N*-Palmitoylethanolamide
