# Supplementary figures and images for: Alpha-linolenic acid modulates systemic and adipose tissue-specific insulin sensitivity, inflammation, and the endocannabinoid system in dairy cows
Source: Sci Rep. 2023 Mar 31;13:5280. doi: 10.1038/s41598-023-32433-7 (PMC10066235; doi:10.1038/s41598-023-32433-7)

Adipose  
tissue

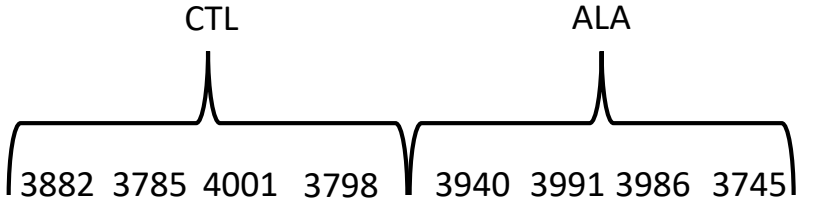

Actin (45 KDa) 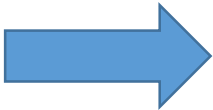

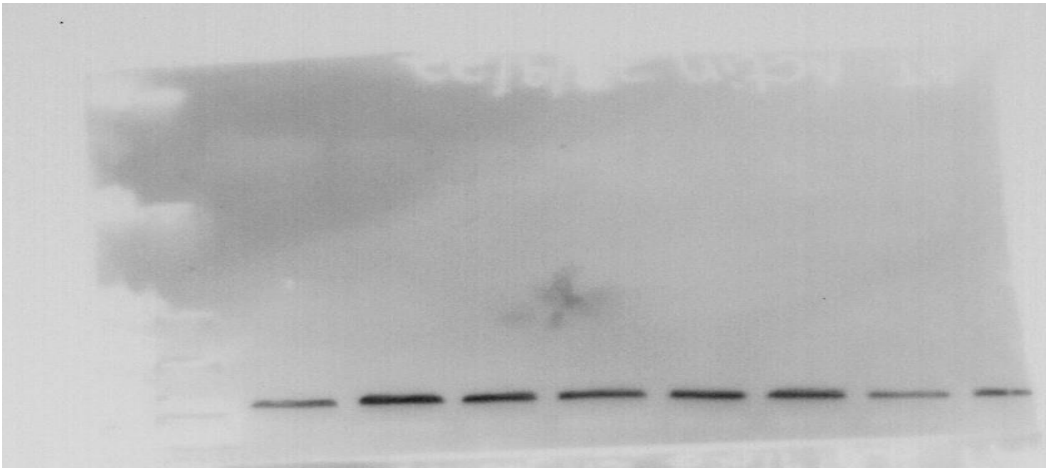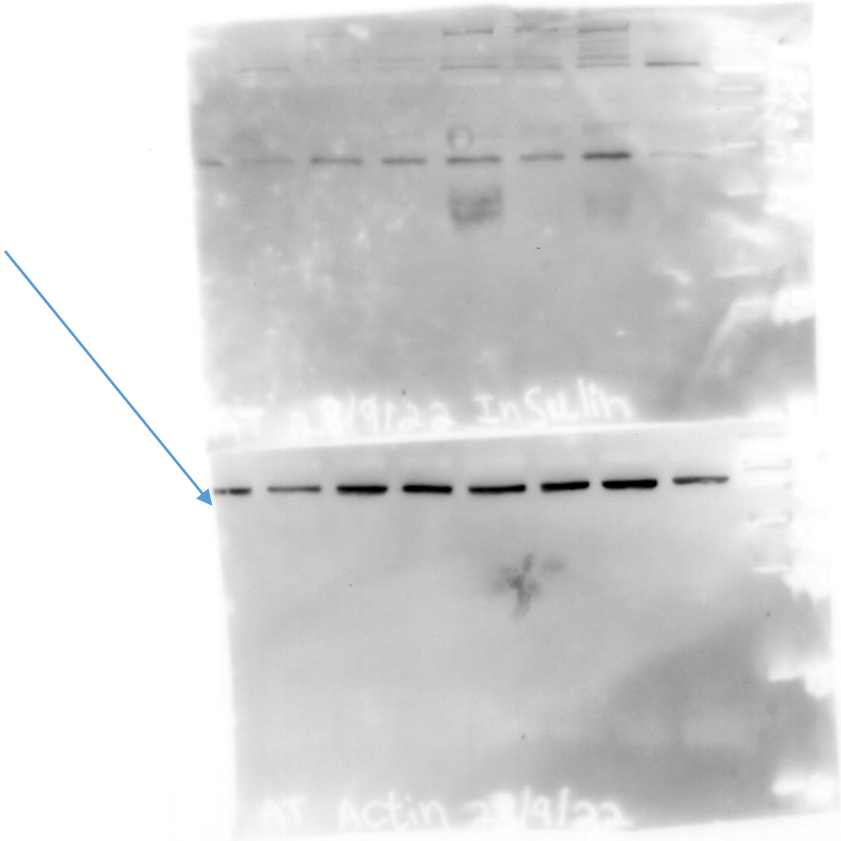

IR $\beta$  (95 KDa)

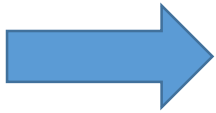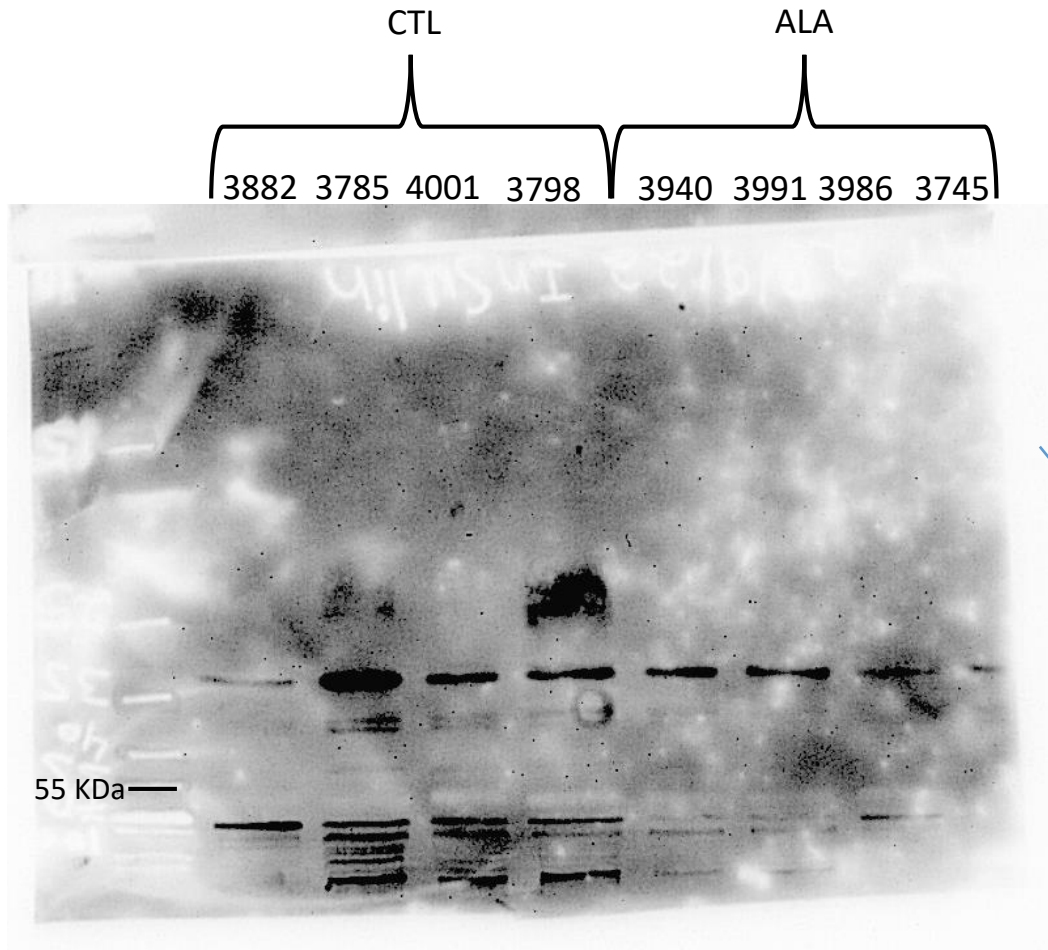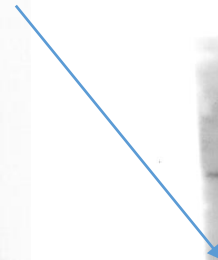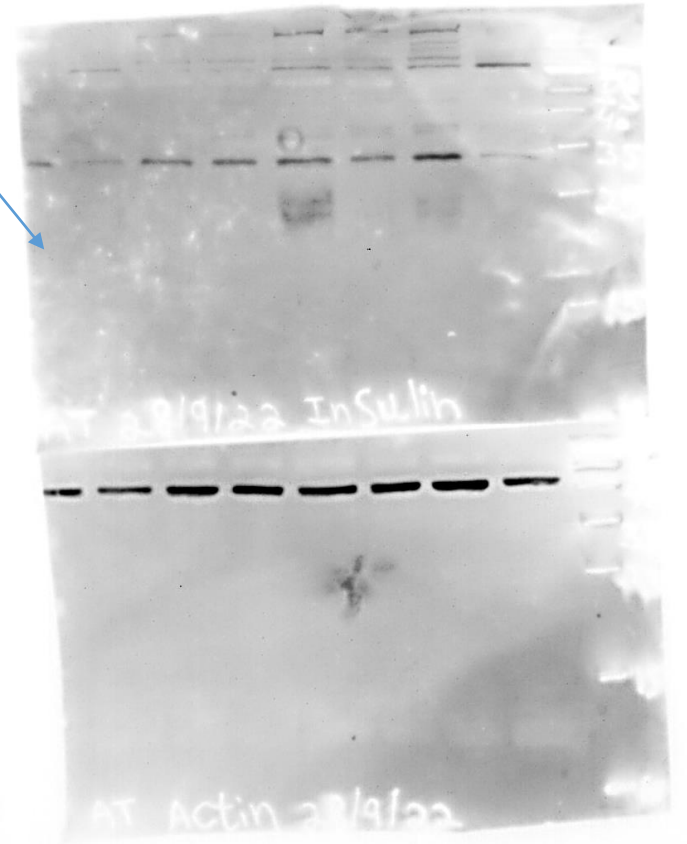

CTL

ALA

3882 3785 4001 3669 3798 3987 3728 3805 3924 3940 3991 3986 3745 3652

AKT (60 KDa)

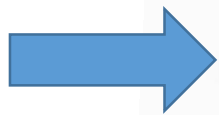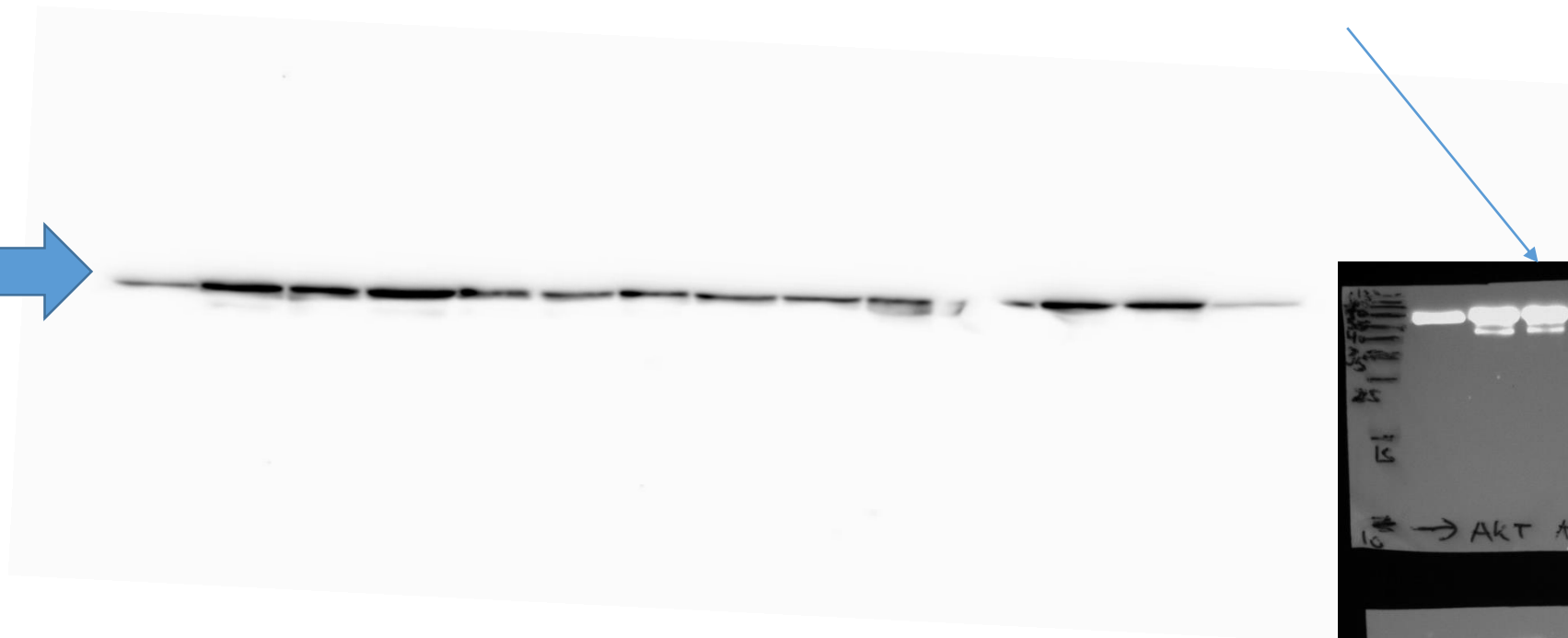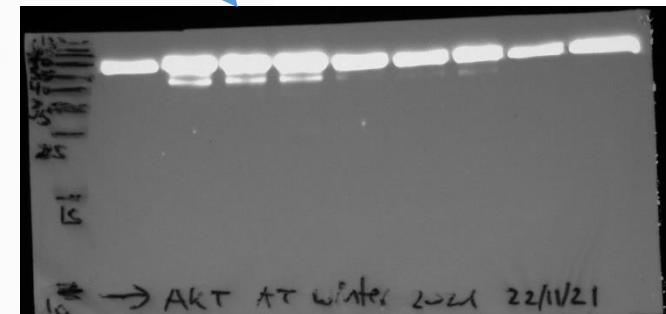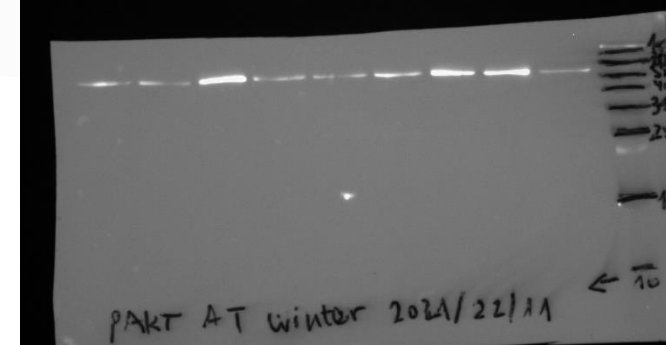

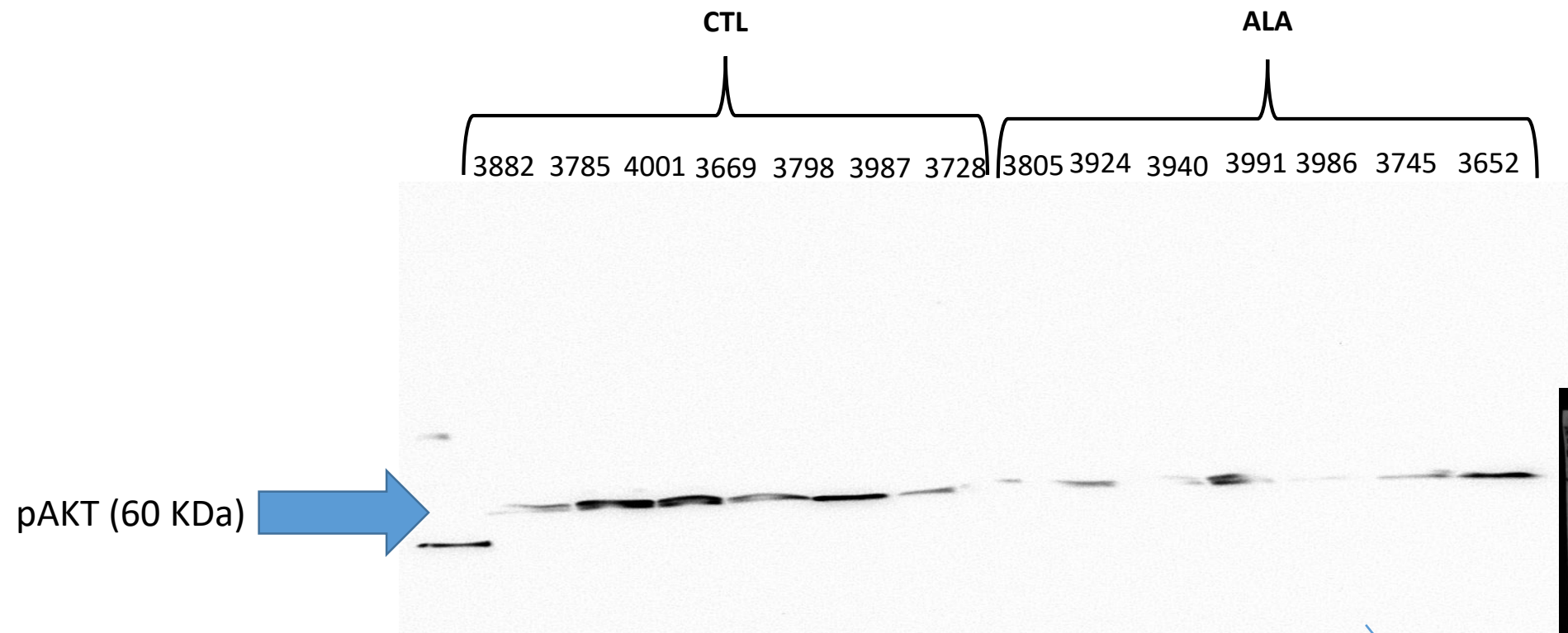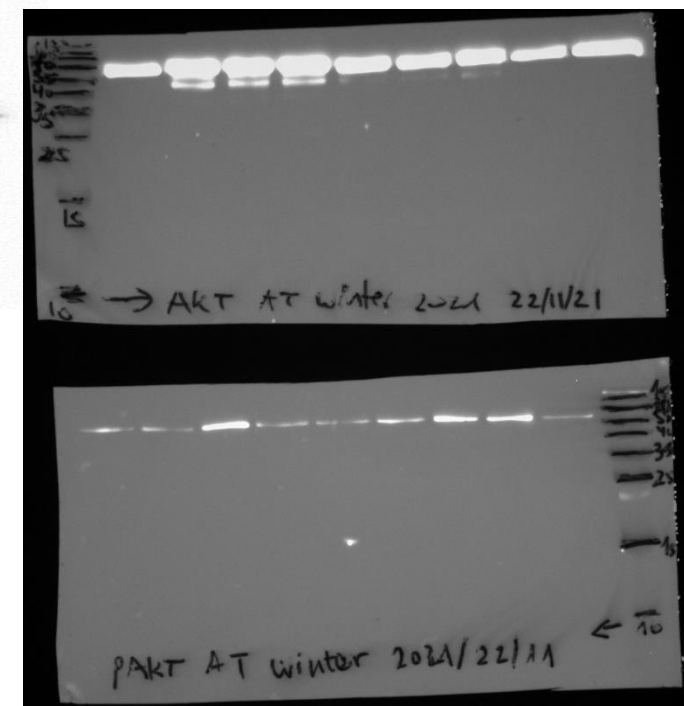

Supplement: Supplementary file 1 — Supplementary Information 1. [file 41598_2023_32433_MOESM1_ESM.pdf]
