# Supplementary material for: Alpha-linolenic acid modulates systemic and adipose tissue-specific insulin sensitivity, inflammation, and the endocannabinoid system in dairy cows
Source: Sci Rep. 2023 Mar 31;13:5280. doi: 10.1038/s41598-023-32433-7 (PMC10066235; doi:10.1038/s41598-023-32433-7)
Supplement: Supplementary file 2 — Supplementary Information 2. [file 41598_2023_32433_MOESM2_ESM.pdf]

PBMC

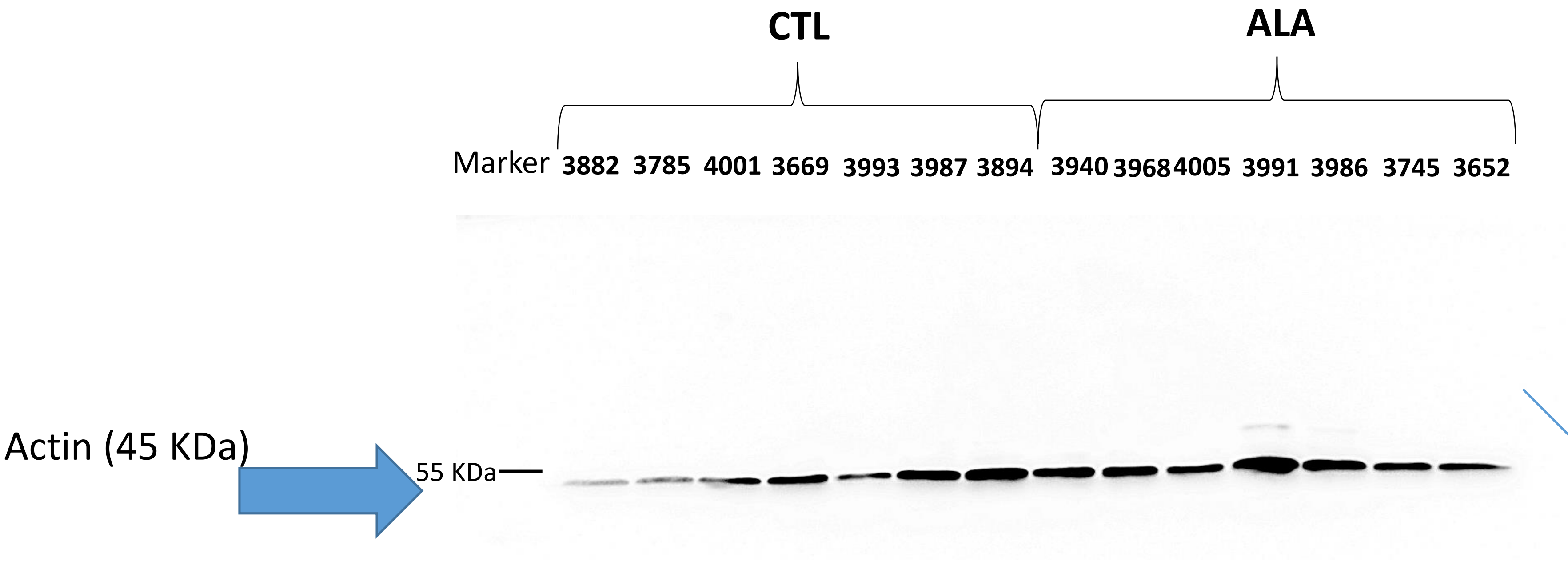

CTL
ALA
Marker
3882
3785
4001
3669
3993
3987
3894
3940
3968
4005
3991
3986
3745
3652
Actin (45 KDa)
55 KDa

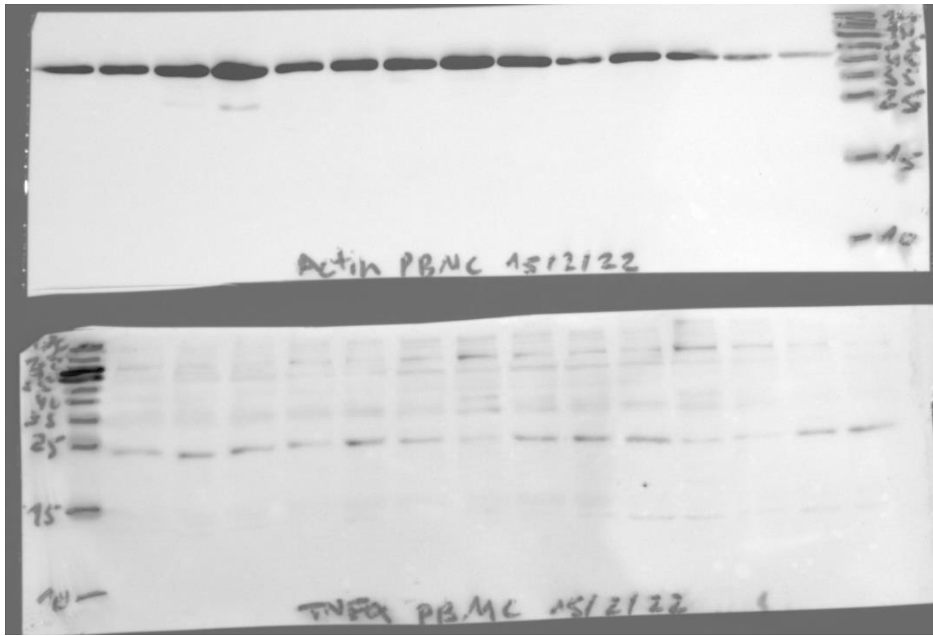

Actin PBMC 15/2/22
TNFα PBMC 15/2/22

PBMC

CTL ALA

Marker 3882 3785 4001 3669 3993 3987 3894 3940 3968 4005 3991 3986 3745 3652

CB1 (60 KDa)

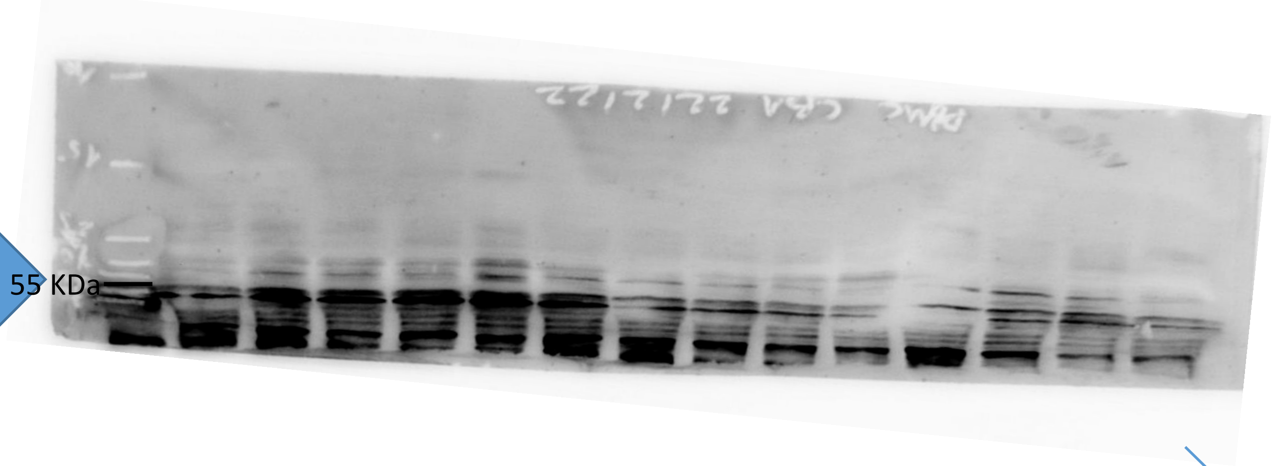

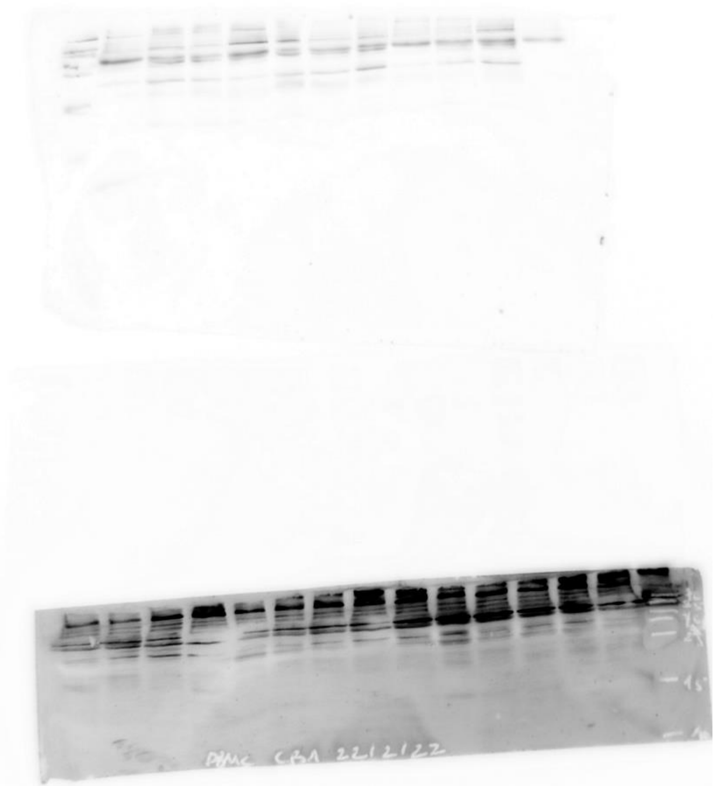

PBMC

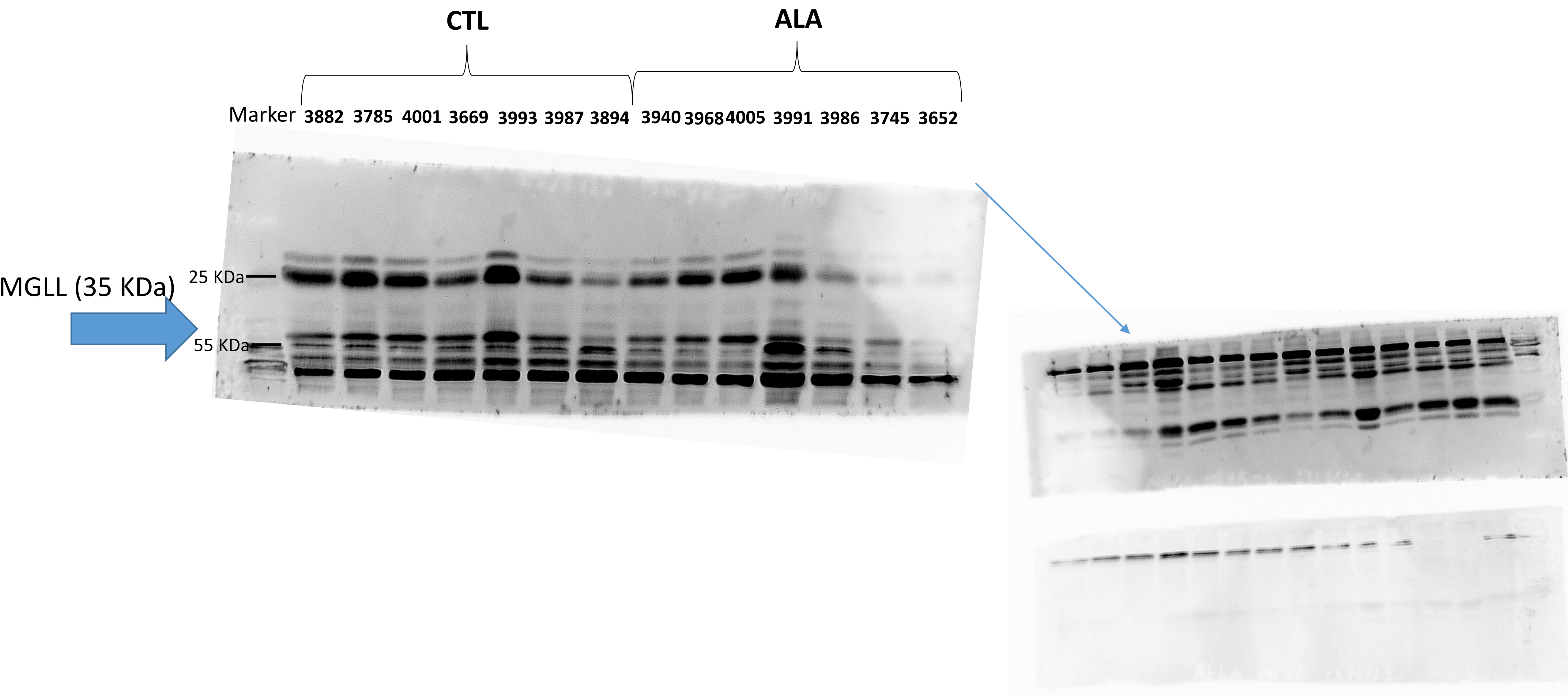
CTL
ALA
Marker
3882 3785 4001 3669 3993 3987 3894 3940 3968 4005 3991 3986 3745 3652
MGLL (35 KDa)
25 KDa
55 KDa

PBMC

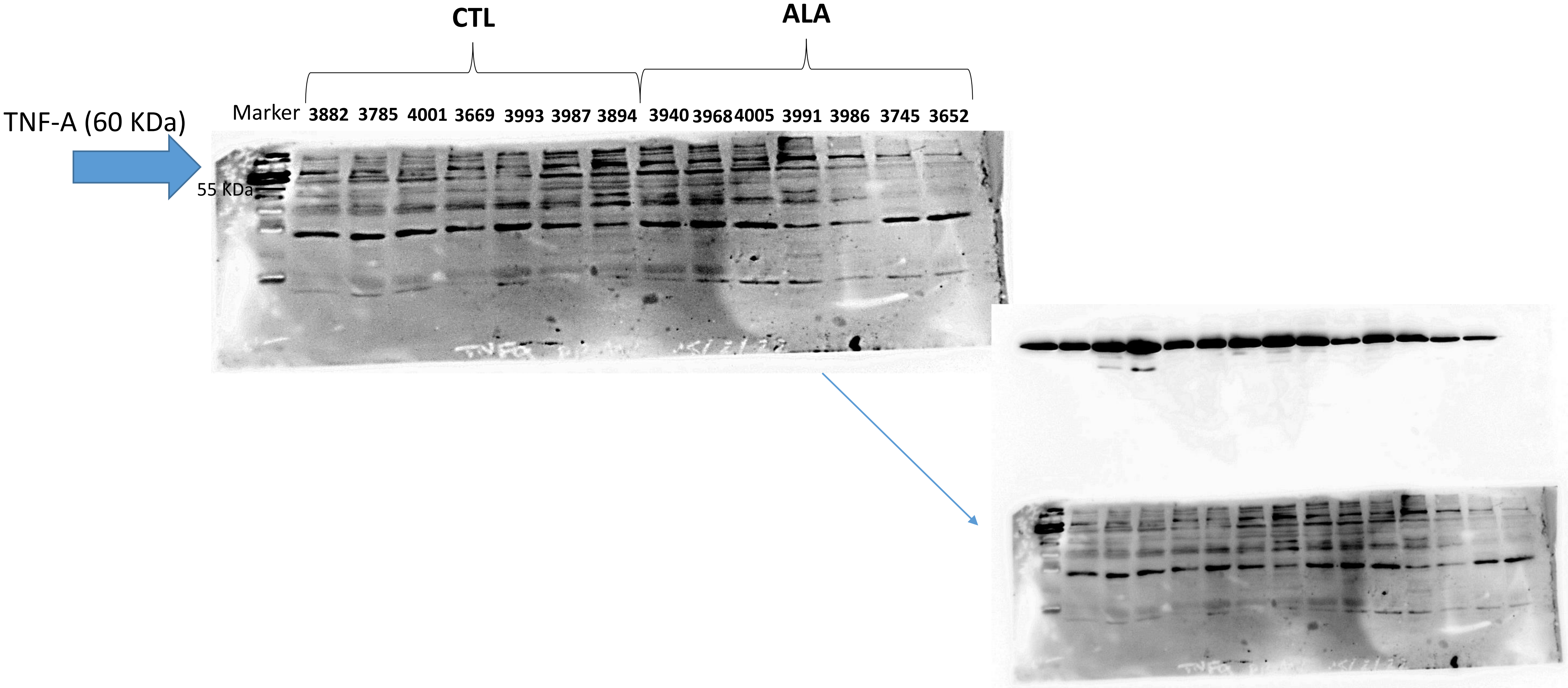
CTL
ALA
TNF-A (60 KDa)
Marker
3882 3785 4001 3669 3993 3987 3894 3940 3968 4005 3991 3986 3745 3652
55 KDa
