# Supplementary material for: Alpha-linolenic acid modulates systemic and adipose tissue-specific insulin sensitivity, inflammation, and the endocannabinoid system in dairy cows
Source: Sci Rep. 2023 Mar 31;13:5280. doi: 10.1038/s41598-023-32433-7 (PMC10066235; doi:10.1038/s41598-023-32433-7)
Supplement: Supplementary file 3 — Supplementary Information 3. [file 41598_2023_32433_MOESM3_ESM.pdf]

Adipose tissue

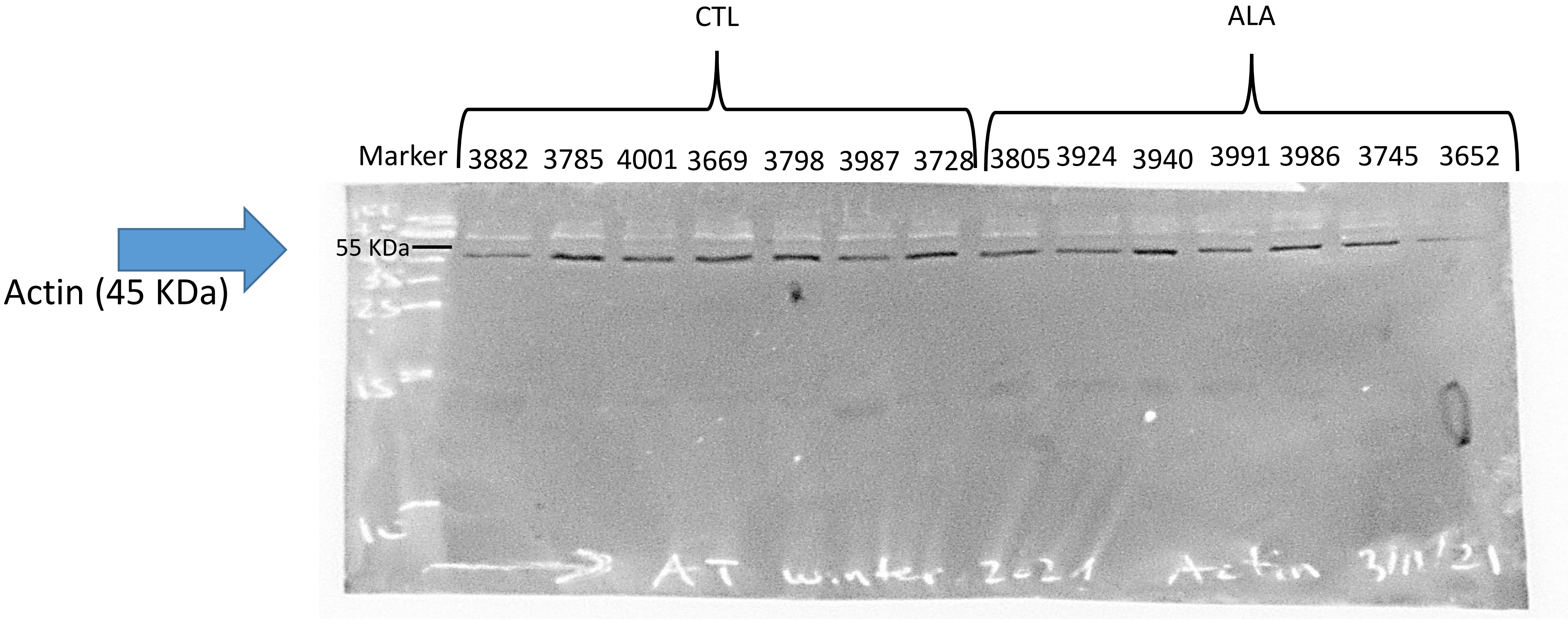

Adipose tissue

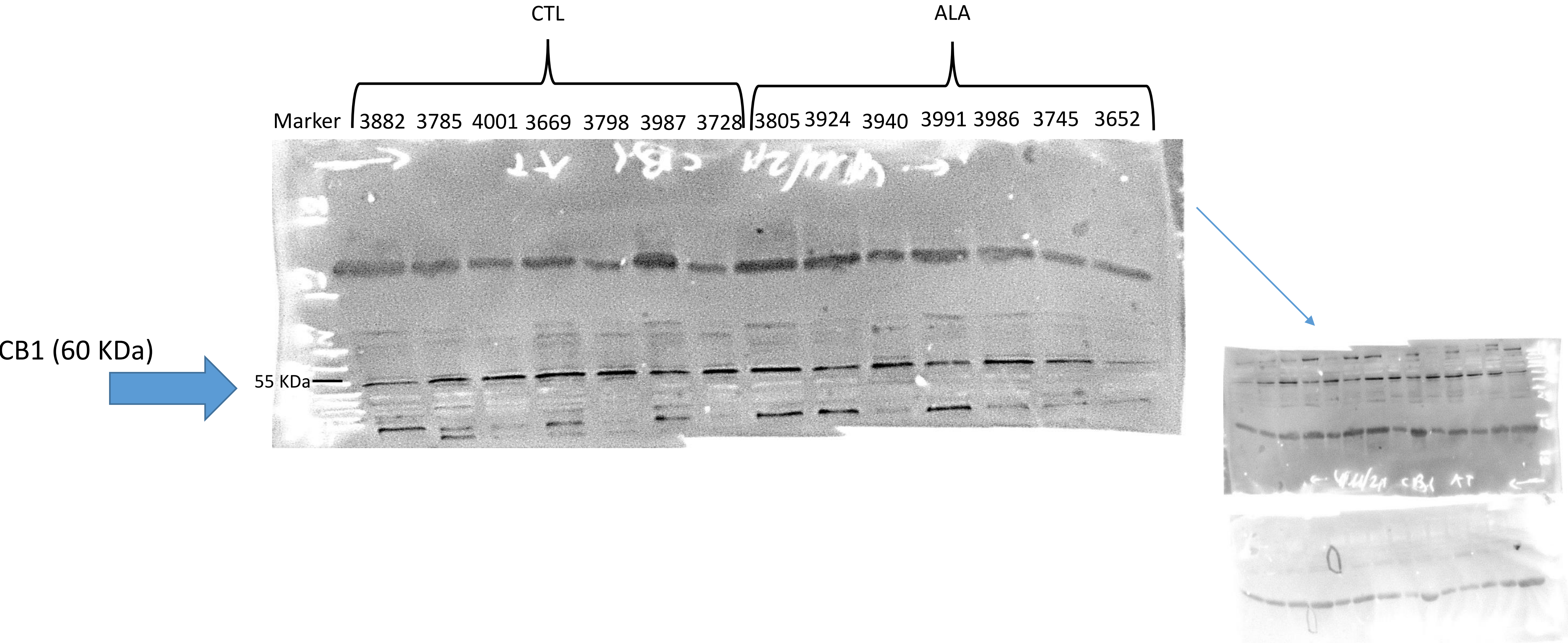

Adipose tissue

CTL

ALA

Marker 3882 3785 4001 3669 3798 3987 3728 3805 3924 3940 3991 3986 3745 3652

CB2 (50 KDa)

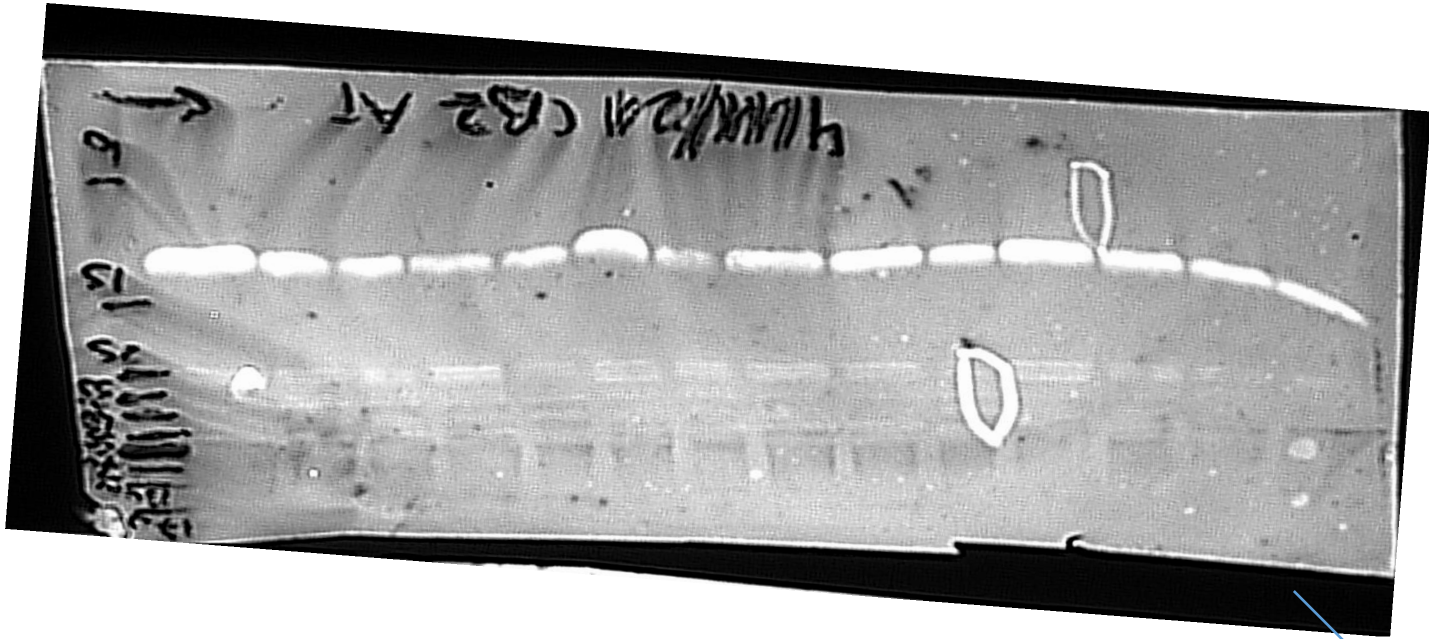

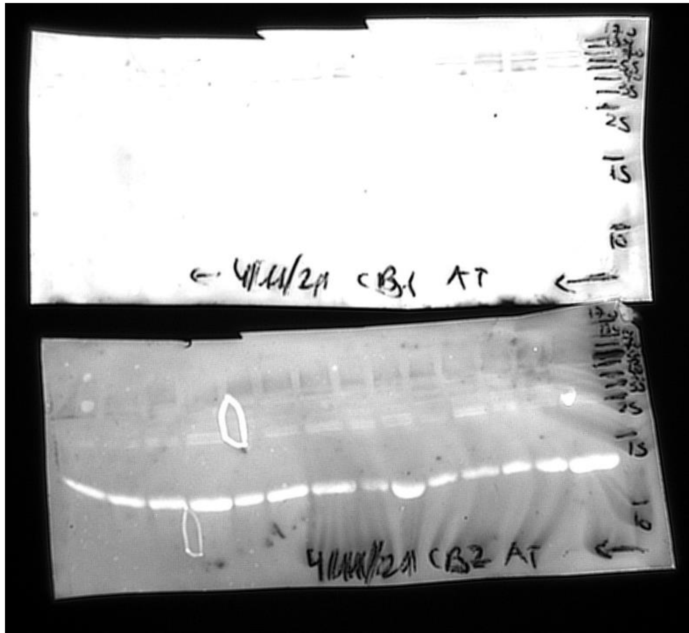

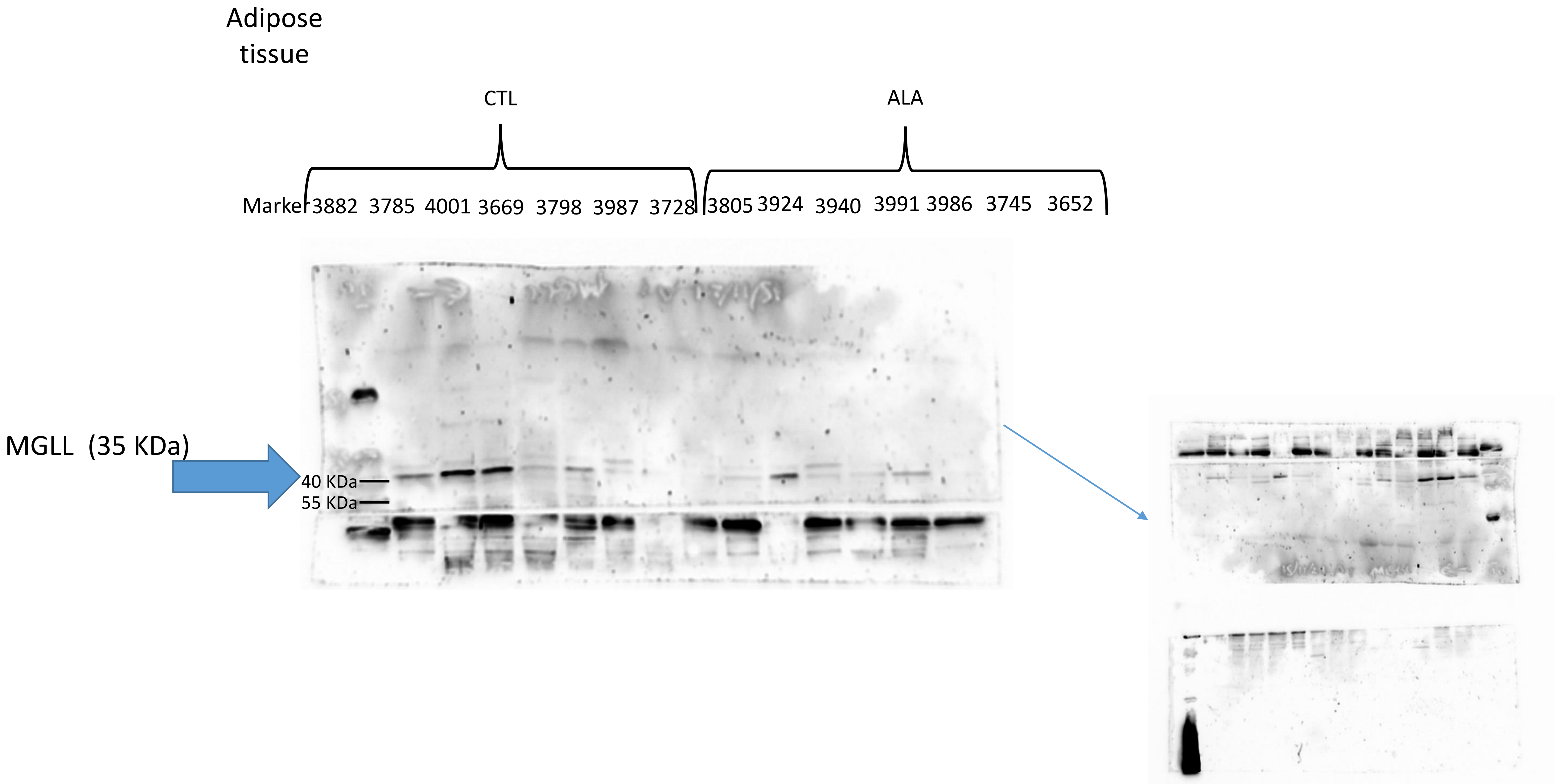
Adipose tissue
CTL
ALA
Marker 3882 3785 4001 3669 3798 3987 3728 3805 3924 3940 3991 3986 3745 3652
MGLL (35 KDa)
40 KDa
55 KDa

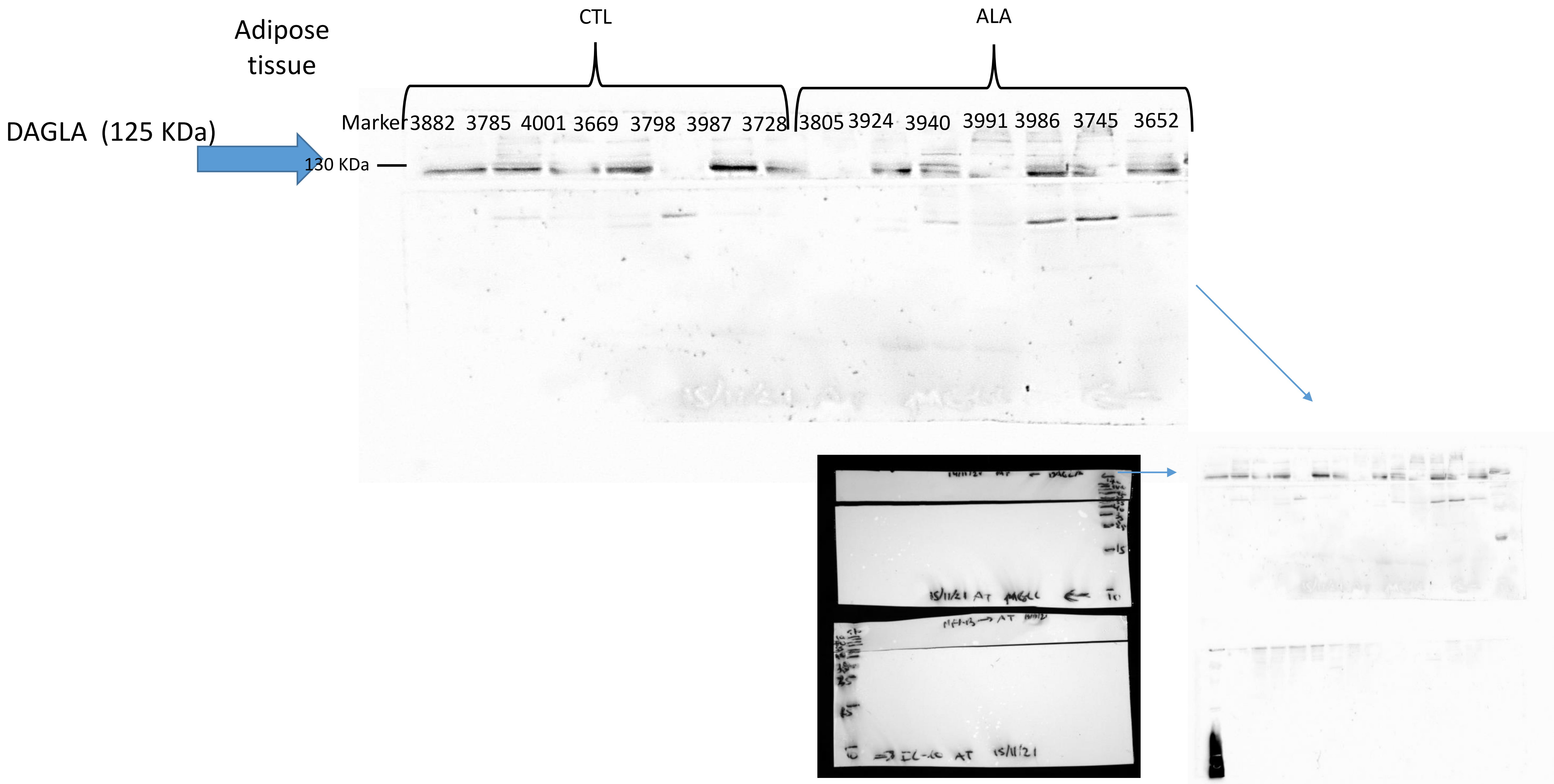
Adipose tissue
DAGLA (125 KDa)
CTL
ALA
Marker 3882 3785 4001 3669 3798 3987 3728 3805 3924 3940 3991 3986 3745 3652
130 KDa

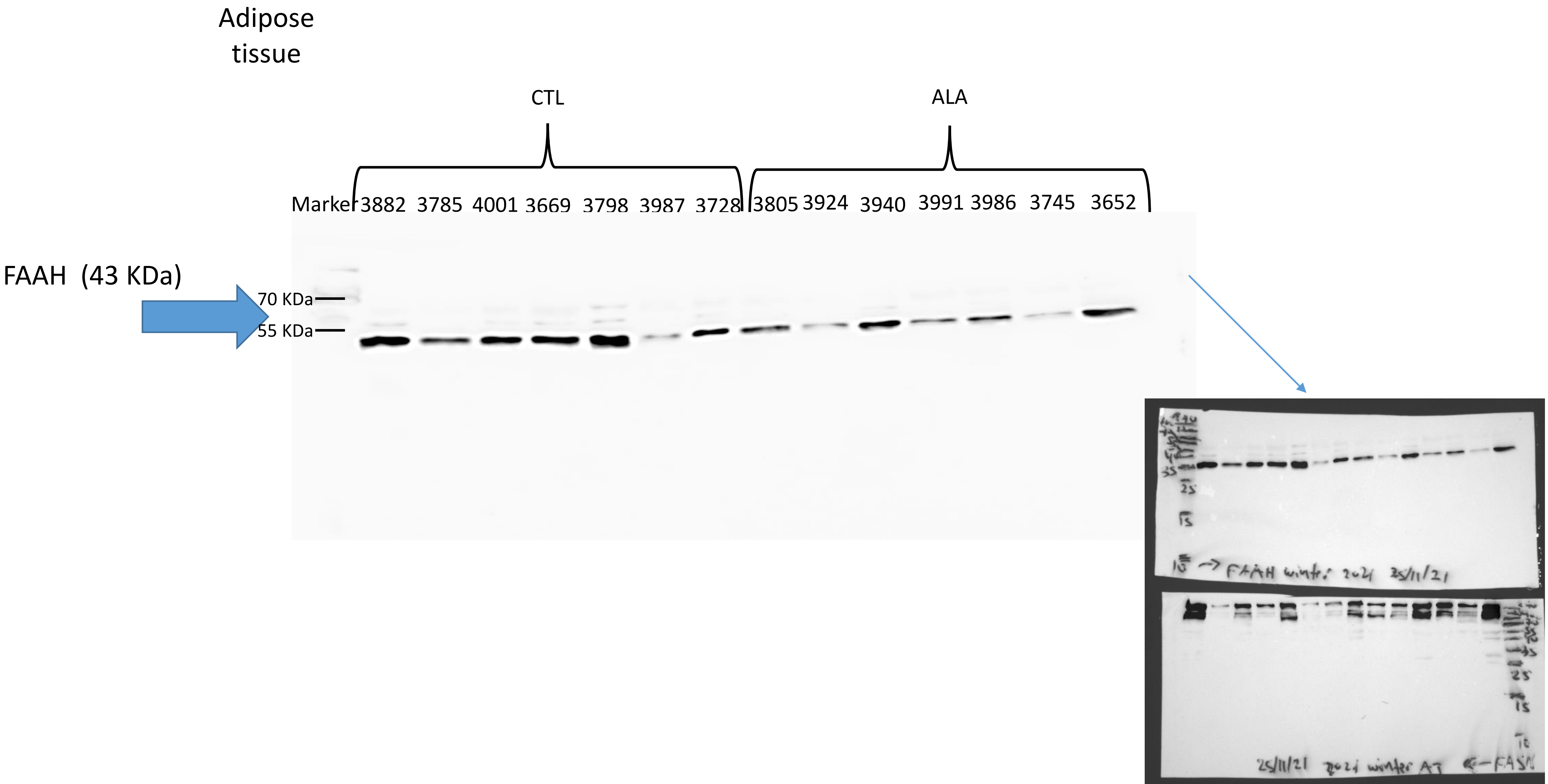
Adipose tissue
CTL
ALA
Marker
3882
3785
4001
3669
3798
3987
3728
3805
3924
3940
3991
3986
3745
3652
FAAH (43 KDa)
70 KDa
55 KDa

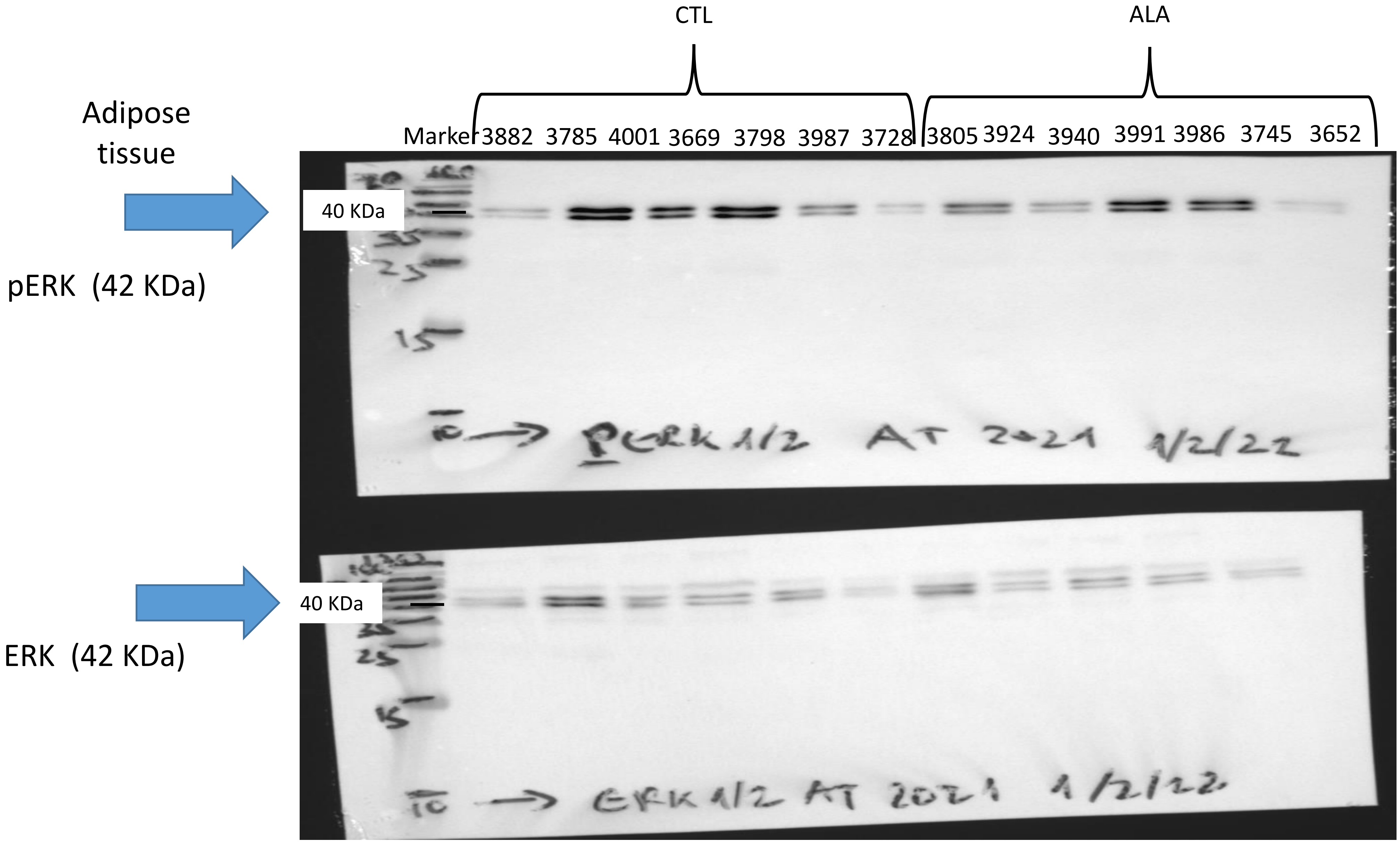
Adipose tissue
CTL
ALA
Marker
3882 3785 4001 3669 3798 3987 3728
3805 3924 3940 3991 3986 3745 3652
40 KDa
pERK (42 KDa)
pERK 1/2 AT 2021 1/2/22
40 KDa
ERK (42 KDa)
ERK 1/2 AT 2021 1/2/22

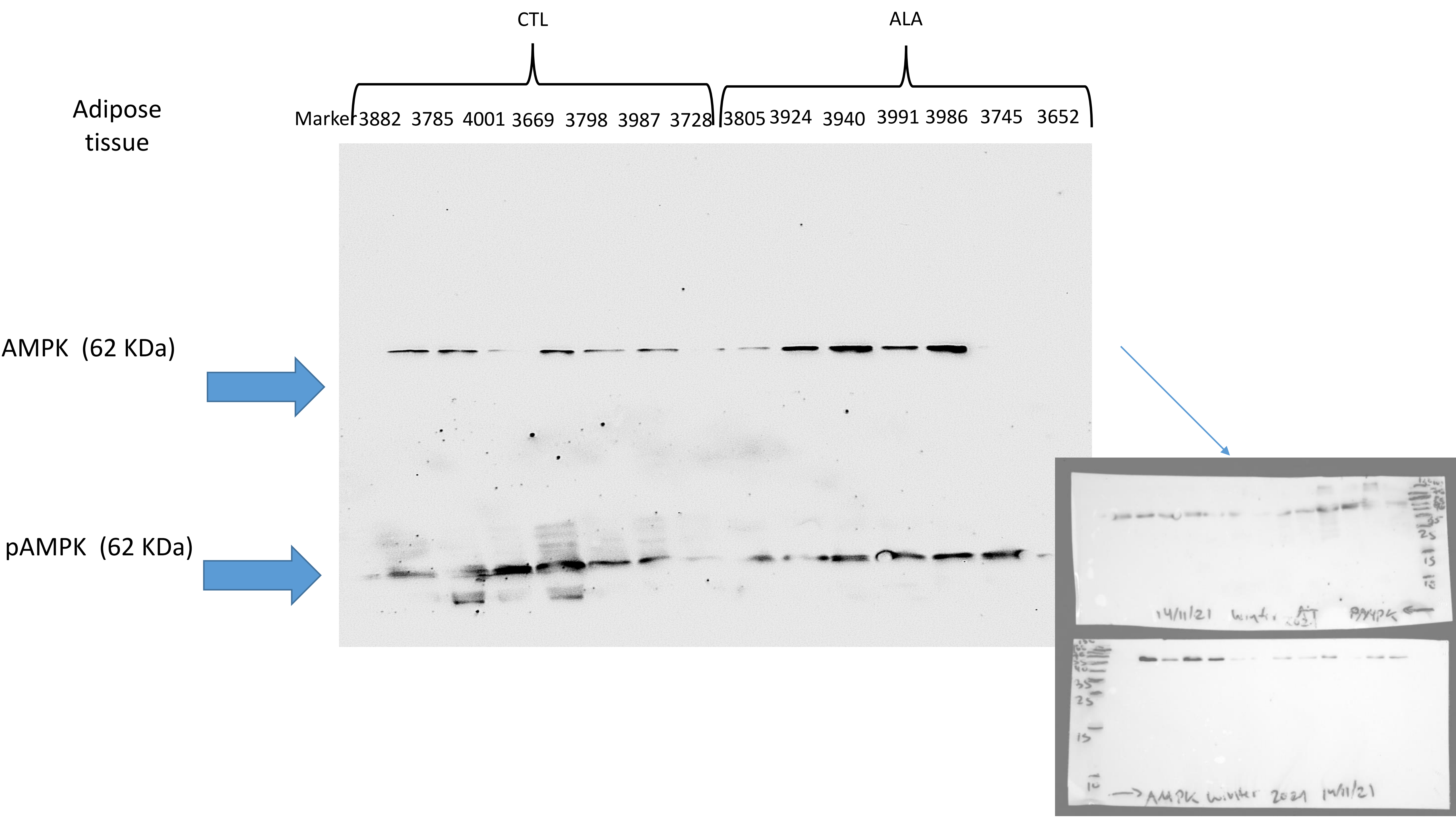
CTL
ALA
Adipose tissue
Marker 3882 3785 4001 3669 3798 3987 3728 3805 3924 3940 3991 3986 3745 3652
AMPK (62 KDa)
pAMPK (62 KDa)
AMPK winter 2021 14/11/21

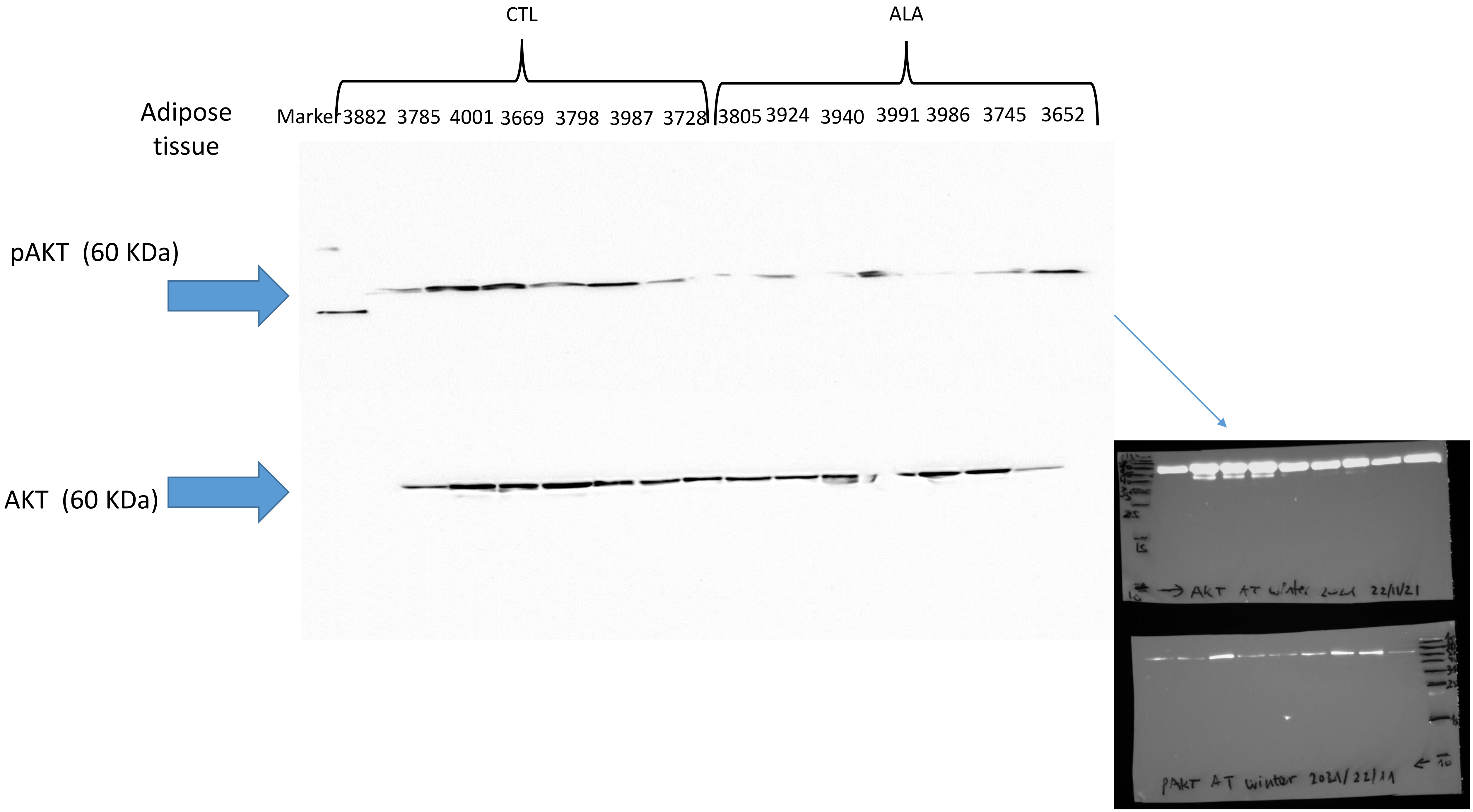

CTL
ALA
Adipose tissue
Marker
3882
3785
4001
3669
3798
3987
3728
3805
3924
3940
3991
3986
3745
3652
pAKT (60 KDa)
AKT (60 KDa)
AKT AT winter 2021 22/11/21
pAKT AT winter 2021/22/11

Adipose tissue

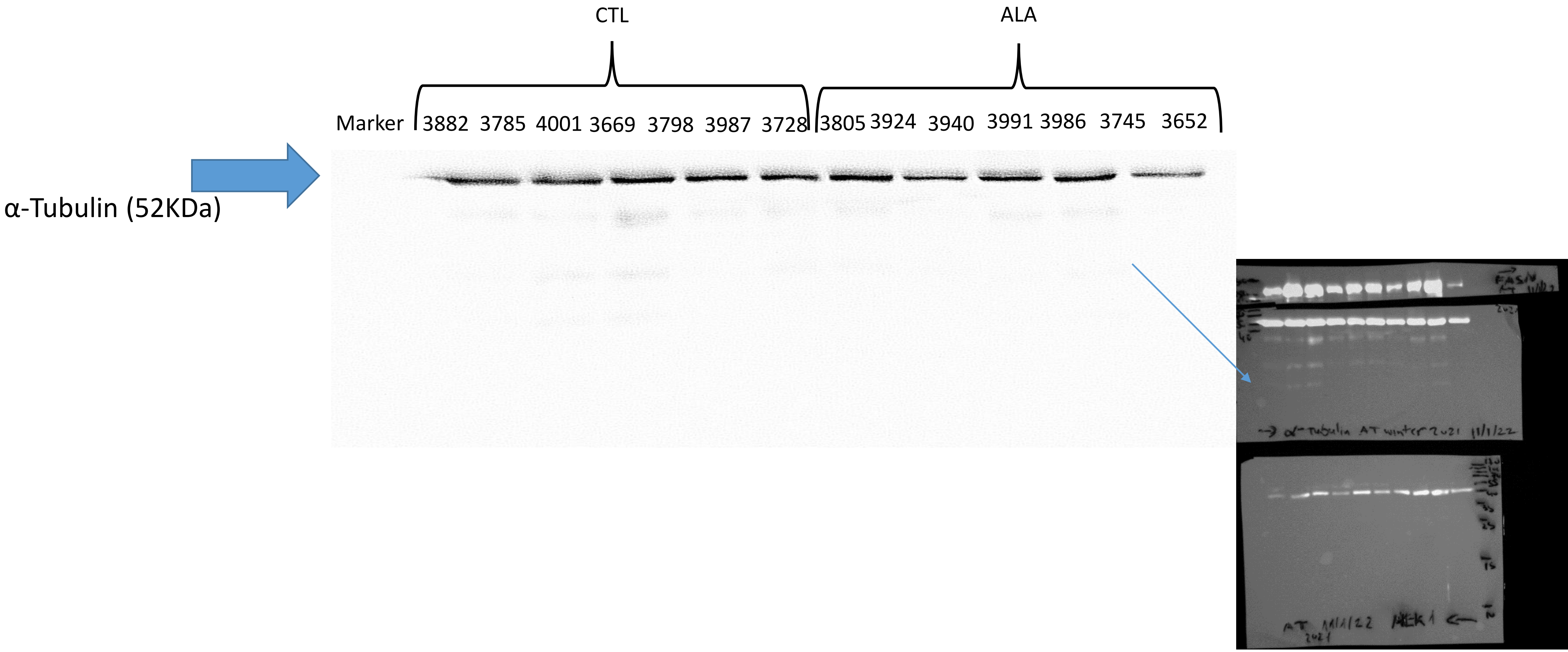

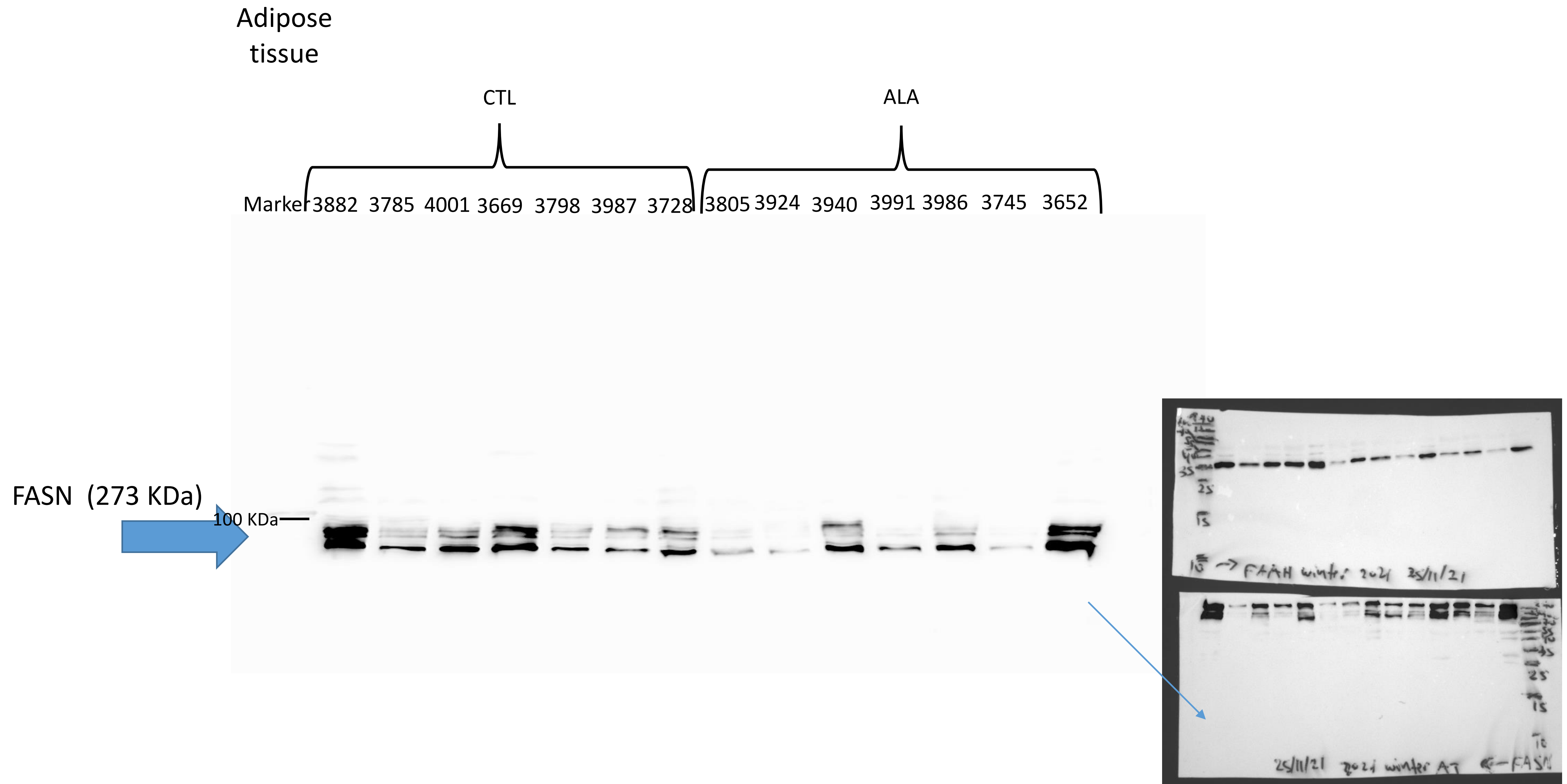
Adipose tissue
CTL
ALA
Marker
3882
3785
4001
3669
3798
3987
3728
3805
3924
3940
3991
3986
3745
3652
FASN (273 KDa)
100 KDa
25/11/21
FASN

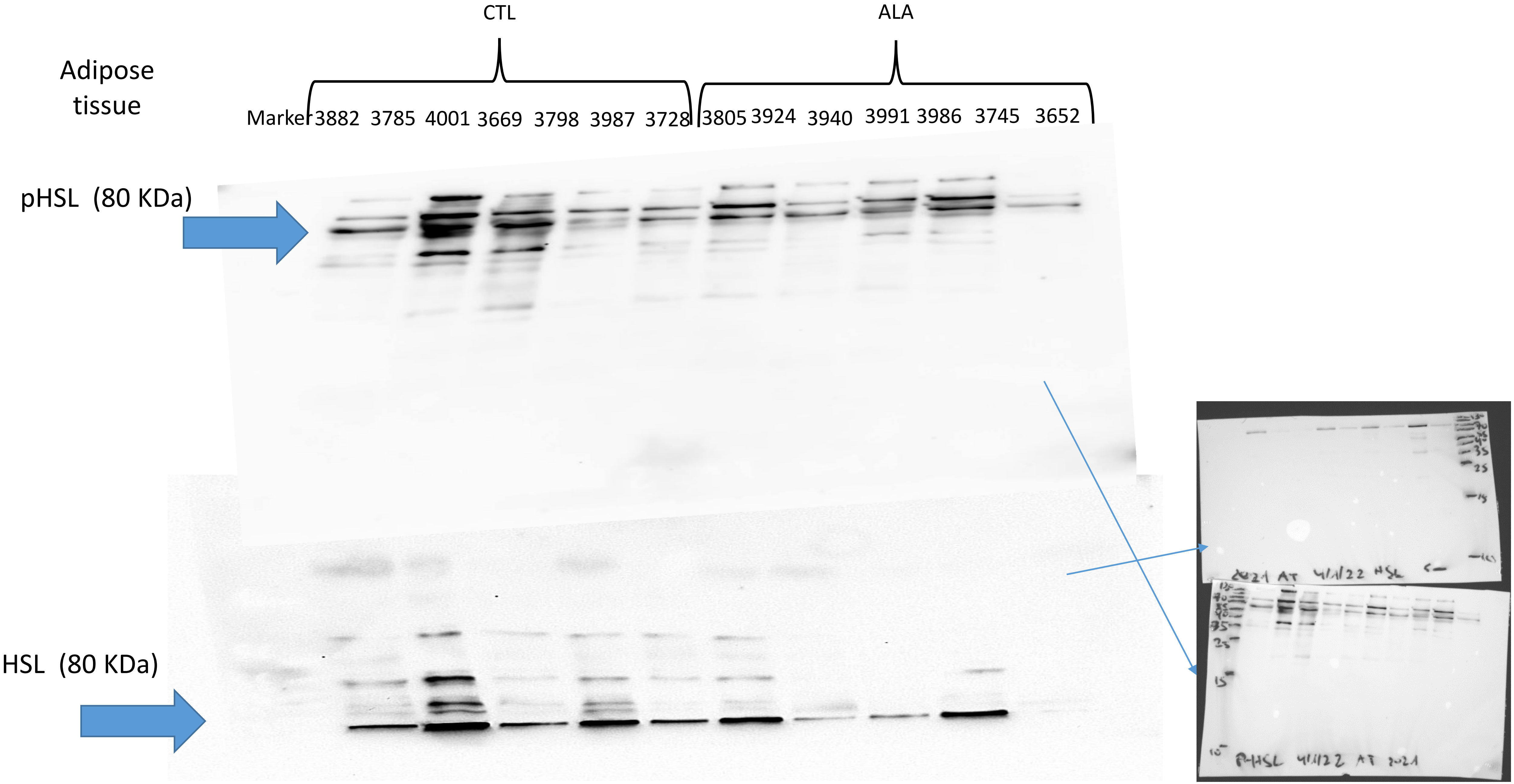
CTL
ALA
Adipose tissue
Marker
3882
3785
4001
3669
3798
3987
3728
3805
3924
3940
3991
3986
3745
3652
pHSL (80 KDa)
HSL (80 KDa)

Adipose tissue

CTL ALA

Marker 3882 3785 4001 3669 3798 3987 3728 3805 3924 3940 3991 3986 3745 3652

TNF-A (60 KDa)

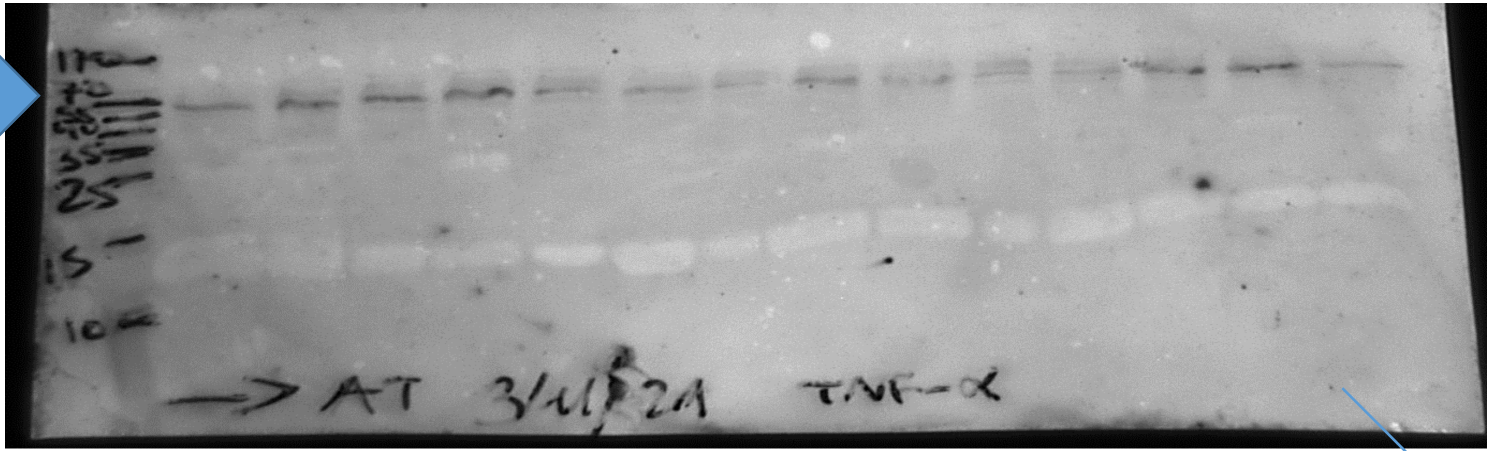

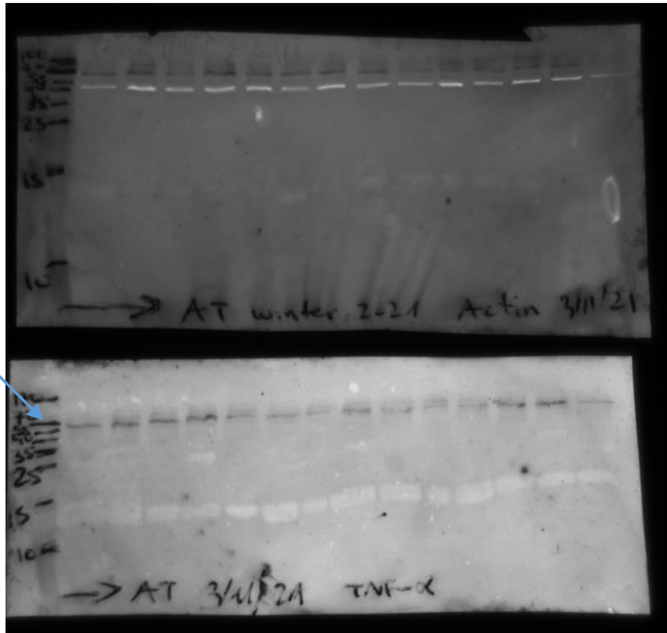

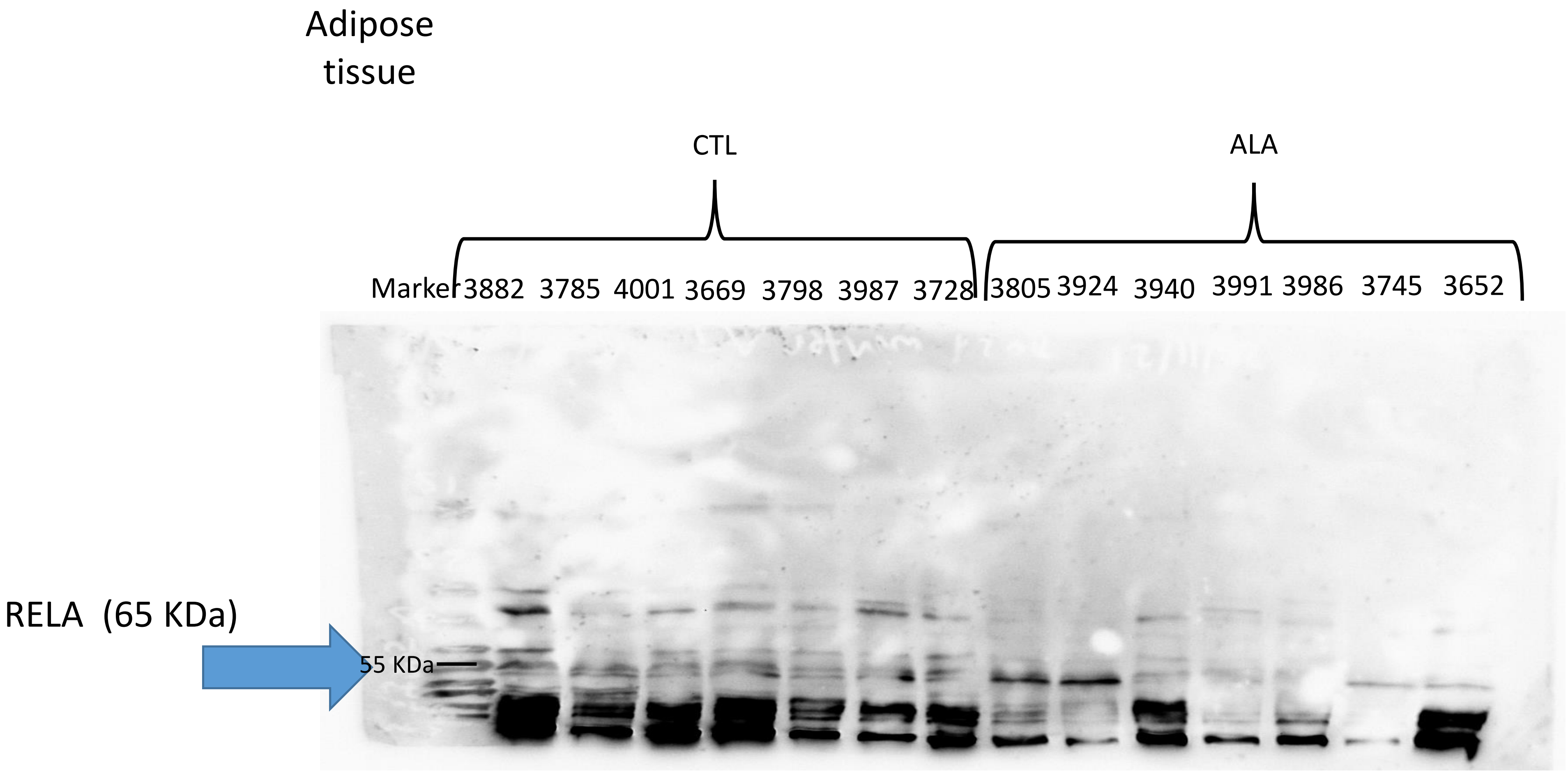
Adipose tissue
CTL
ALA
Marker 3882 3785 4001 3669 3798 3987 3728 3805 3924 3940 3991 3986 3745 3652
RELA (65 KDa)
55 KDa
