# Supplementary material for: Alpha-linolenic acid modulates systemic and adipose tissue-specific insulin sensitivity, inflammation, and the endocannabinoid system in dairy cows
Source: Sci Rep. 2023 Mar 31;13:5280. doi: 10.1038/s41598-023-32433-7 (PMC10066235; doi:10.1038/s41598-023-32433-7)
Supplement: Supplementary file 4 — Supplementary Information 4. [file 41598_2023_32433_MOESM4_ESM.docx]

**Supplementary Materials**

**Supplementary Table 1.** Ingredients, chemical composition fatty acids profile of the fat supplements of the postpartum (PP) experimental diets.

|  |  | | | **Treatment^1^** | | | |
| --- | --- | --- | --- | --- | --- | --- | --- |
| **Item (% of DM)** | | **CTL** | | | **ALA** | | |
| Corn grain, ground | | |  | 21.9 | | 21.5 | |
| Barley grain, rolled | | |  | 1.8 | | 1.3 | |
| Wheat grain, |  | | | 2.7 | | 2.7 | |
| Soybean meal |  | | | 3.5 | | 1.9 | |
| Rapeseed meal |  | | | 6.2 | | 6.2 | |
| Gluten feed |  | | | 10.3 | | 10.6 | |
| Golden DDG^2^ |  | | | 6.6 | | 4.1 | |
| Cottonseed |  | | | 1.0 | | 1.0 | |
| Wheat bran |  | | | 3.5 | | 3.5 | |
| Wheat silage |  | | | 14.8 | | 15.0 | |
| Corn silage |  | | | 6.0 | | 6.0 | |
| Oat hay |  | | | 13.5 | | 13.5 | |
| Clover hay |  | | | 1.5 | | 1.3 | |
| Valomega |  | | | 0.0 | | 6.4 | |
| CSFA |  | | | 2.4 | | 0.0 | |
| Whey |  | | | 2.5 | | 1.6 | |
| Urea |  | | | 0.2 | | 0.2 | |
| Limestone |  | | | 0.3 | | 0.7 | |
| Calcium bicarbonate |  | | | 0.7 | | 0.7 | |
| Salt |  | | | 0.9 | | 1.2 | |
| Vitamins and minerals |  | | | 0.01 | | 0.01 | |
| Chemical composition |  | | |  | | | |
| NE_L_^3^ (Mcal/kg DM) |  | | | 1.8 | | 1.8 | |
| Crude protein, % |  | | | 16.5 | | 16.5 | |
| NDF^4^, % |  | | | 30.5 | | 30.5 | |
| Forage NDF, % |  | | | 17.5 | | 17.5 | |
| Ether extract, % | | |  | 5.3 | | 5.0 | |
| Calcium, % | | |  | 0.9 | | 0.9 | |
| Phosphorus, % | | | 0.5 | | | | 0.5 |
| C18:2n-6, % | | | 34.2 | | | | 32.0 |
| C16:0, % | | | 24.8 | | | | 13.7 |
| C18:0, % | | | 4.0 | | | | 3.8 |
| C18:3n-3, % | | | 3.8 | | | | 24.5 |

^1^ Dairy cows were divided into two nutritional groups from -21- 60 days PP; (i) CTL – saturated fat, (ii) ALA–flaxseed supplement providing α-linolenic acid.^2^DDG, dried distillers grain. Contained 20,000,000 IU/kg vitamin A, 2,000,000 IU/kg vitamin D, 15,000 IU/kg vitamin E, 6000 mg/kg Mn, 6000 mg/kg Zn, 2000 mg/kg Fe, 1500 mg/kg Cu, 120 mg/kg I, 50 mg/kg Se, and 20 mg/kg Co. ^3^NE_L_, net energy for lactation calculated using NRC. ^4^NDF, neutral detergent fiber.

**Supplementary Table 2**. List of immunoblot Ab's used to measure protein abundance.

| Antigen | Abbreviation | Host | Dilution | | | Source |
| --- | --- | --- | --- | --- | --- | --- |
| Cannabinoid receptor1 | CB1 | Rabbit | | 1:200 | Abcam biotech, Cambridge, UK, ab23703 | |
| Cannabinoid receptor2 | CB2 | Rabbit | | 1µg/ml | Enzo, NY, USA, ADI-905-820-100 | |
| Monoglyceride lipase | MGLL | Rabbit | | 1:200 | Abcam biotech, Cambridge, UK, ab24701 | |
| Fatty acid amide hydrolase | FAAH | Rabbit | | 1µg/ml | Aviva systems biology, CA, USA, ARP33121_P050 | |
| Diacylglycerol lipase alpha | DAGLA | Goat | | 0.3µg/ml | Aviva systems biology, CA, USA, OAEB01139 | |
| Insulin receptor beta | IRβ | Rabbit | | 1:1000 | Cell signaling, MA, USA, 3025 | |
| Ras-dependent extracellular signal-regulated kinase | ERK1\2 | Rabbit | | 1:2000 | Cell signaling, MA, USA, 4695 | |
| Phspho- Ras-dependent extracellular signal-regulated kinase Tyr204 | pERK1\2 | Rabbit | | 1:2000 | Cell signaling, MA, USA, 4370 | |
| 5' AMP-activated protein kinase | AMPK | Rabbit | | 1:1000 | Cell signaling, MA, USA, 5832 | |
| Phospho-5' AMP-activated protein kinase | pAMPK | Rabbit | | 1:1000 | Cell signaling, MA, USA, 2531 | |
| Protein kinase B | AKT | Rabbit | | 1:1000 | Cell signaling, MA, USA, 9271 | |
| Phospho-Protein kinase B S473 | pAKT |  | |  |  | |
| α-tubulin | α-tubulin | Rabbit | | 1:1000 | Cell signaling, MA, USA, 2125S | |
| Tumor necrosis factor α | TNF-α | Rabbit | | 1:1000 | Aviva systems biology, CA, USA, OACA04183 | |
| Nuclear factor kappa B | NFκB | Rabbit | | 1:1000 | Cell signaling, MA, USA, 4717 | |
| Hormone sensitive lipase | HSL | Rabbit | | 1:1000 | Cell signaling, MA, USA, 4107P | |
| Phospho-HSL S660pAb | pHSL | Rabbit | | 1:1000 | Cell signaling, MA, USA, 4126P | |
| Fatty acid synthase | FASN | Rabbit | | 1:2000 | Abcam biotech, Cambridge, UK, ab99359 | |

**Supplementary Table 3.** Fatty acid (FA) profile of plasma postpartum (PP) in dairy cows supplemented with ALA.

|  | | **Treatment^1^** | | |  |  |
| --- | --- | --- | --- | --- | --- | --- |
| **FA (%)** | | **CTL** | **ALA** | **SEM** | | ***P*-value** |
| C18:2n-6 | 40.3 | 39.7 | 1.81 | | 0.79 |  |
| C18:3n-6 | 0.7 | 0.6 | 0.05 | | 0.09 |  |
| C18:3n-3 | 1.6^a^ | 4.5^b^ | 0.50 | | <0.001 |  |
| C20:1n9 | 0.7 | 0.8 | 0.07 | | 0.07 |  |
| C20:3 | 1.8^a^ | 1.3^b^ | 0.11 | | 0.001 |  |
| C20:4n-3 | 0.2^a^ | 0.3^b^ | 0.02 | | 0.01 |  |
| C20:4n-6 | 2.3 | 1.9 | 0.18 | | 0.16 |  |
| C22:5n3 | 0.4^a^ | 0.2^b^ | 0.05 | | 0.01 |  |
| C22:6n3 | 2.4 | 1.9 | 0.34 | | 0.30 |  |
| Saturated FA | 35.5 | 33.6 | 2.22 | | 0.54 |  |
| MUFA^2^ | 12.9^a^ | 14.1^b^ | 0.44 | | 0.05 |  |
| PUFA^3^ | 51.1 | 51.7 | 2.26 | | 0.84 |  |
| n-3 | 4.9^a^ | 7.2^b^ | 0.50 | | <0.01 |  |
| n-6 | 43.3 | 42.2 | 1.91 | | 0.69 |  |
| n-6:n-3 | 11.7^a^ | 6.4^b^ | 1.07 | | <0.01 |  |

^a-b^ Values with different superscript letters in a row are significantly different at P < 0.05.

^1^ Dairy cows were divided into two nutritional groups from -21- 60 days PP; (i) CTL –saturated fat, (ii) ALA – flaxseed supplement providing α-linolenic acid (ALA). n=12.^2^monounsaturated FA; ^3^polyunsaturated FA.


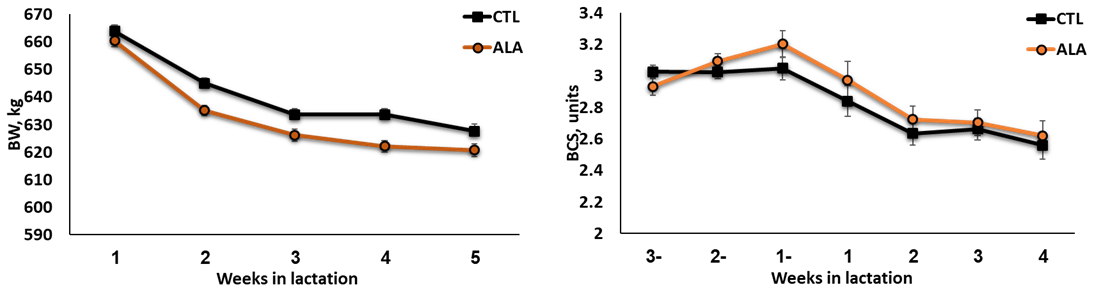


**Supplementary Figure 1**. **Body condition score (BCS) of postpartum (PP) dairy cows supplemented peripartum with ALA.** The BCS was assessed on a weekly basis. Dairy cows were divided into two nutritional groups from -21- 60 days PP; (i) CTL –saturated fat, (ii) ALA–flaxseed supplement providing α-linolenic acid (ALA).

**Supplementary Table 4**. Average concentrations of plasma metabolites postpartum (PP) in dairy cows supplemented with ALA.

|  | | | **Treatment^1^** | | | | | | |  | | | |  |  |  |
| --- | --- | --- | --- | --- | --- | --- | --- | --- | --- | --- | --- | --- | --- | --- | --- | --- |
|  | | | **CTL** | **ALA** | | | | | **SEM** | | ***P*-value** | | |  |  |  |
| **Item** | | | | | |  | |  | | | | |  | |  | |
|  | Glucose, mg/dL | 66.8 | | | | 64.6 | | 1.39 | | | | 0.26 | | | |  |
|  | NEFA^2^, µEq/L | 406.6 | | | | 492.8 | | 36.99 | | | | 0.11 | | | |  |
|  | TG^3^, mg/dL | | 9.6 | | | 9.4 | | | | 0.35 | | | 0.73 |  |  |  |
|  | AST^4^, u/L | | 78.8 | | 72.7 | | | | | 3.60 | | | 0.24 |  |  |  |
|  | BHBA^5^, mg/dL | | 0.6^a^ | | | | 0.5^b^ | | | 0.03 | | | 0.02 |  |  |  |
|  | Cortisol, ng/mL | | 11.2 | | | 14.0 | | | | 3.05 | | | 0.54 |  |  |  |
|  | Insulin, uIU/mL | | 17.8 | | | 15.6 | | | | 2.06 | | | 0.47 |  |  |  |

^a-b^ Values with different superscript letters in a row are significantly different at *P* < 0.05.

^1^ Dairy cows were divided into two nutritional groups from -21- 60 days PP; (i) CTL – saturated fat, (ii) ALA –flaxseed supplement providing α-linolenic acid (ALA). ^2^Non-esterified fatty acid; ^3^Triglyceride; ^4^Aspartate Aminotransferase; ^5^β-Hydroxybutyrate acid.

**Supplementary Table 5.** Complete blood count of postpartum dairy cows supplemented with ALA.

|  | **Treatment^1^** | | | |  |  |  |  |  |
| --- | --- | --- | --- | --- | --- | --- | --- | --- | --- |
| **Item** | | **CTL** | | **ALA** | | **SEM** | ***P*-value** | |  |
| Neutrophils, % | | | 48.9 | 47.9 | | 2.94 | | 0.82 | |
| Monocytes, % | | | 7.5 | 8.5 | | 1.01 | | 0.47 | |
| Lymphocytes, % | | | 40.4 | 40.4 | | 2.56 | | 0.99 | |
| Basophiles, % | | | 1.1^a^ | 1.2^b^ | | 0.06 | | 0.03 | |
| Eosinophils, % | | | 1.3 | 1.3 | | 0.17 | | 0.95 | |
| Reticulocytes, % | | | 0.1 | 0.1 | | 0.02 | | 0.40 | |
| LUC^2^, % | | | 0.8 | 0.6 | | 0.26 | | 0.53 | |
| WBC^3^, 10.e3/uL | | | 8.2 | 7.2 | | 0.41 | | 0.12 | |
| Neutrophils, 10.e3/uL | | | 4.1 | 3.6 | | 0.34 | | 0.39 | |
| Monocytes, 10.e3/uL | | | 0.6 | 0.6 | | 0.08 | | 0.84 | |
| Lymphocytes, 10.e3/uL | | | 3.2^a^ | 2.8^b^ | | 0.22 | | 0.18 | |
| Basophiles, 10.e3/uL | | | 0.1 | 0.09 | | 0.01 | | 0.49 | |
| Eosinophils, 10.e3/uL | | | 0.1 | 0.09 | | 0.01 | | 0.42 | |
| Reticulocytes, 10.e9/L | | | 33.1 | 33.50 | | 1.60 | | 0.87 | |
| LUC^2^, 10.e3/uL | | | 0.1 | 0.04 | | 0.02 | | 0.41 | |
| RBC^3^, 10.e6/uL | | | 6.1 | 6.21 | | 0.08 | | 0.21 | |
| Platelet, 10.e3/uL | | | 9.0 | 8.67 | | 0.24 | | 0.32 | |
| RDW^4^, % | | | 19.9 | 19.82 | | 0.23 | | 0.74 | |
| HCT^5^, % | | | 28.7^a^ | 30.84^b^ | | 0.51 | | 0.01 | |
| HGB^6^, g/dL | | | 10.0^a^ | 10.59^b^ | | 0.18 | | 0.03 | |
| MCV^7^, fL | | | 47.5 | 49.29 | | 0.66 | | 0.07 | |
| MCV^8^ Reticulocytes | | | 60.2 | 64.98 | | 1.61 | | 0.06 | |
| HDW^9^, g/dL | | | 2.3 | 2.29 | | 0.06 | | 0.67 | |
| CH^10^, | | | 18.3 | 19.47 | | 0.45 | | 0.08 | |
| CHCM^11^, g/dL | | | 33.3 | 33.19 | | 0.18 | | 0.78 | |
| MCH^12^, pg | | | 16.5 | 17.10 | | 0.29 | | 0.19 | |
| MCHC^13^, g/dL | | | 34.8 | 34.22 | | 0.27 | | 0.18 | |
| CHCM^14^ Reticulocytes g/dL | | | 31.0 | 30.46 | | 0.32 | | 0.22 | |
| MPV^15^, fL | | | 0.5 | 0.38 | | 0.04 | | 0.21 | |
| PCT^16^, ng/ml | | | 40.1 | 37.99 | | 2.14 | | 0.49 | |
| PDW^17^, % | | | 9.4^a^ | 7.14^b^ | | 0.15 | | <0.001 | |
| CHDW^18^, Ratio | | | 506.9 | 435.56 | | 48.36 | | 0.31 | |

^a-b^ Values with different superscript letters in a row are significantly different at *P* < 0.05.

^1^Dairy cows were divided into two nutritional groups from -21- 60 days PP; (i) CTL– saturated fat, (ii) ALA –flaxseed supplement providing α-linolenic acid (ALA).^2^Large unstained cells;^3^Red blood cells;^4^Red blood cell distribution width;^5^Hematocrit;^6^Hemoglobin;^7^Mean corpuscular volume;^8^Mean cell volume;^9^Hemoglobin distribution width;^10^Cellular hemoglobin concentration mean;^11^Mean corpuscular hemoglobin;^12^Mean corpuscular hemoglobin concentration;^13^Mean platelet volume;^14^Procalcitonin test;^15^Platelet distribution width;^16^Cell hemoglobin distribution width;^17^Cell hemoglobin distribution width.

**Supplementary Table 6:** Significant phosphopetides in ALA vs. CTL adipose tissue

| S.no | Protein | Fasta headers | Gene | Student's T-test p-value 1_2 | Fold Change 1/2 |
| --- | --- | --- | --- | --- | --- |
| 1 | A0A3Q1LUD1 | tr\|A0A3Q1LUD1\|A0A3Q1LUD1_BOVIN KN motif and ankyrin repeat domains 3 OS=Bos taurus OX=9913 GN=KANK3 PE=4 SV=1;tr\|E1BNU3\|E1BNU3_BOVIN KN motif and ankyrin repeat domains 3 OS=Bos taurus OX=9913 GN=KANK3 PE=4 SV=3;tr\|A0A3Q1MKV0\|A0A3Q1MKV0_BOVIN KN motif and | KANK3 | 5.83E-10 | 2079.741 |
| 2 | P02253 | sp\|P02253\|H12_BOVIN Histone H1.2 OS=Bos taurus OX=9913 GN=H1-2 PE=1 SV=2;tr\|A0A452DIF5\|A0A452DIF5_BOVIN H1.2 linker histone, cluster member OS=Bos taurus OX=9913 GN=H1-2 PE=3 SV=1 | H1-2 | 3.22E-09 | -629.719 |
| 3 | P08814 | sp\|P08814\|PTMS_BOVIN Parathymosin OS=Bos taurus OX=9913 GN=PTMS PE=1 SV=2;tr\|A6QPM9\|A6QPM9_BOVIN PTMS protein OS=Bos taurus OX=9913 GN=PTMS PE=2 SV=1 | PTMS | 2.88E-08 | 741.2426 |
| 4 | A0A3Q1ND55 | tr\|A0A3Q1ND55\|A0A3Q1ND55_BOVIN 2-hydroxyacyl-CoA lyase 1 OS=Bos taurus OX=9913 GN=HACL1 PE=1 SV=1;tr\|F1MVP8\|F1MVP8_BOVIN 2-hydroxyacyl-CoA lyase 1 OS=Bos taurus OX=9913 GN=HACL1 PE=1 SV=3;tr\|A5PJL6\|A5PJL6_BOVIN HACL1 protein OS=Bos taurus OX=9913 GN=HACL1 | HACL1 | 1.84E-07 | -2797.66 |
| 5 | Q2KJH6 | sp\|Q2KJH6\|SERPH_BOVIN Serpin H1 OS=Bos taurus OX=9913 GN=SERPINH1 PE=2 SV=1 | SERPIN | 7.86E-07 | -984.321 |
| 6 | A0A3Q1M0D6 | tr\|A0A3Q1M0D6\|A0A3Q1M0D6_BOVIN Serine/arginine repetitive matrix 2 OS=Bos taurus OX=9913 GN=SRRM2 PE=4 SV=1;tr\|F1MI03\|F1MI03_BOVIN Serine/arginine repetitive matrix 2 OS=Bos taurus OX=9913 GN=SRRM2 PE=4 SV=2;tr\|A0A3Q1MP73\|A0A3Q1MP73_BOVIN Serine/arginine r | SRRM2 | 0.002566 | 256.0934 |
| 7 | A0A3Q1MSB2 | tr\|A0A3Q1MSB2\|A0A3Q1MSB2_BOVIN A-kinase anchoring protein 12 OS=Bos taurus OX=9913 GN=AKAP12 PE=1 SV=1;tr\|G3MWT9\|G3MWT9_BOVIN A-kinase anchoring protein 12 OS=Bos taurus OX=9913 GN=AKAP12 PE=1 SV=2 | AKAP12 | 0.003264 | 213.8408 |
| 8 | Q1RMQ9 | tr\|Q1RMQ9\|Q1RMQ9_BOVIN CDC42 effector protein (Rho GTPase binding) 4 OS=Bos taurus OX=9913 GN=CDC42EP4 PE=2 SV=1;tr\|A0A3Q1MPX4\|A0A3Q1MPX4_BOVIN CRIB domain-containing protein OS=Bos taurus OX=9913 GN=CDC42EP4 PE=3 SV=1 | CDC42E | 0.003765 | 409.4374 |
| 9 | A0A3Q1LZ84 | tr\|A0A3Q1LZ84\|A0A3Q1LZ84_BOVIN Catenin delta 1 OS=Bos taurus OX=9913 GN=CTNND1 PE=1 SV=1;tr\|A0A3Q1MQD5\|A0A3Q1MQD5_BOVIN Catenin delta 1 OS=Bos taurus OX=9913 GN=CTNND1 PE=1 SV=1;tr\|A0A3Q1LLH4\|A0A3Q1LLH4_BOVIN Catenin delta 1 OS=Bos taurus OX=9913 GN=CTNND1 | CTNND1 | 0.003872 | 217.6744 |
| 10 | E1BLT2 | tr\|E1BLT2\|E1BLT2_BOVIN SAFB like transcription modulator OS=Bos taurus OX=9913 GN=SLTM PE=4 SV=3;tr\|A0A3Q1NB02\|A0A3Q1NB02_BOVIN SAFB like transcription modulator OS=Bos taurus OX=9913 GN=SLTM PE=4 SV=1 | SLTM | 0.004036 | 275.7248 |
| 11 | P48616 | sp\|P48616\|VIME_BOVIN Vimentin OS=Bos taurus OX=9913 GN=VIM PE=1 SV=3 | VIM | 0.004037 | -208.082 |
| 12 | Q0VC46 | tr\|Q0VC46\|Q0VC46_BOVIN GC-rich sequence DNA-binding factor homolog OS=Bos taurus OX=9913 GN=GCFC PE=2 SV=1 | GCFC | 0.004048 | 3737.086 |
| 13 | A5D984 | tr\|A5D984\|A5D984_BOVIN Pyruvate kinase OS=Bos taurus OX=9913 GN=PKM PE=1 SV=1;tr\|Q3ZC87\|Q3ZC87_BOVIN Pyruvate kinase (Fragment) OS=Bos taurus OX=9913 GN=PKM2 PE=2 SV=1 | PKM | 0.004053 | 282.7009 |
| 14 | F1MTZ0 | tr\|F1MTZ0\|F1MTZ0_BOVIN Protein phosphatase inhibitor 2 OS=Bos taurus OX=9913 GN=PPP1R2 PE=1 SV=1;sp\|Q3SZX2\|IPP2_BOVIN Protein phosphatase inhibitor 2 OS=Bos taurus OX=9913 GN=PPP1R2 PE=2 SV=1 | PPP1R2 | 0.004072 | 1022.59 |
| 15 | F1MCK2 | tr\|F1MCK2\|F1MCK2_BOVIN AHNAK nucleoprotein OS=Bos taurus OX=9913 GN=AHNAK PE=1 SV=3;tr\|A0A3Q1NE82\|A0A3Q1NE82_BOVIN AHNAK nucleoprotein OS=Bos taurus OX=9913 GN=AHNAK PE=1 SV=1 | AHNAK | 0.004145 | 215.5093 |
| 16 | A0A3Q1M5S0 | tr\|A0A3Q1M5S0\|A0A3Q1M5S0_BOVIN Laminin subunit alpha 4 OS=Bos taurus OX=9913 GN=LAMA4 PE=1 SV=1;tr\|A0A3Q1M8W5\|A0A3Q1M8W5_BOVIN Laminin subunit alpha 4 OS=Bos taurus OX=9913 GN=LAMA4 PE=1 SV=1;tr\|A0A3Q1LGL7\|A0A3Q1LGL7_BOVIN Laminin subunit alpha 4 OS=Bos ta | LAMA4 | 0.004177 | 958.5562 |
| 17 | E1BF96 | tr\|E1BF96\|E1BF96_BOVIN MHC class I region proline-rich protein CAT53 OS=Bos taurus OX=9913 GN=PPP1R10 PE=4 SV=2 | PPP1R1 | 0.00418 | -151.741 |
| 18 | A0A3Q1NIS1 | tr\|A0A3Q1NIS1\|A0A3Q1NIS1_BOVIN Non-specific serine/threonine protein kinase OS=Bos taurus OX=9913 GN=ULK1 PE=4 SV=1;tr\|F1MFB2\|F1MFB2_BOVIN Non-specific serine/threonine protein kinase OS=Bos taurus OX=9913 GN=ULK1 PE=4 SV=3 | ULK1 | 0.004184 | 289.0085 |
| 19 | Q3MHL8 | sp\|Q3MHL8\|SMAP_BOVIN Small acidic protein OS=Bos taurus OX=9913 GN=SMAP PE=2 SV=1 | SMAP | 0.004193 | -1352.43 |
| 20 | A5D7T6 | tr\|A5D7T6\|A5D7T6_BOVIN YY1 protein OS=Bos taurus OX=9913 GN=YY1 PE=2 SV=1 | YY1 | 0.004196 | 124.1933 |
| 21 | G3MYP5 | tr\|G3MYP5\|G3MYP5_BOVIN Microtubule associated protein 1A OS=Bos taurus OX=9913 GN=MAP1A PE=4 SV=2;tr\|A0A3Q1M9Z4\|A0A3Q1M9Z4_BOVIN Microtubule associated protein 1A OS=Bos taurus OX=9913 GN=MAP1A PE=4 SV=1 | MAP1A | 0.004221 | 529.7862 |
| 22 | F1MCK2 | tr\|F1MCK2\|F1MCK2_BOVIN AHNAK nucleoprotein OS=Bos taurus OX=9913 GN=AHNAK PE=1 SV=3;tr\|A0A3Q1NE82\|A0A3Q1NE82_BOVIN AHNAK nucleoprotein OS=Bos taurus OX=9913 GN=AHNAK PE=1 SV=1;tr\|A0A3Q1MHT0\|A0A3Q1MHT0_BOVIN AHNAK nucleoprotein OS=Bos taurus OX=9913 GN=AHNA | AHNAK | 0.004237 | 171.7675 |
| 23 | A0A3Q1M024 | tr\|A0A3Q1M024\|A0A3Q1M024_BOVIN Protocadherin 1 OS=Bos taurus OX=9913 GN=PCDH1 PE=4 SV=1;tr\|F6Q0C0\|F6Q0C0_BOVIN Protocadherin 1 OS=Bos taurus OX=9913 GN=PCDH1 PE=4 SV=2;tr\|A4IFK1\|A4IFK1_BOVIN PCDH1 protein OS=Bos taurus OX=9913 GN=PCDH1 PE=2 SV=1 | PCDH1 | 0.004272 | 352.6751 |
| 24 | G3X696 | tr\|G3X696\|G3X696_BOVIN Non-specific serine/threonine protein kinase OS=Bos taurus OX=9913 GN=SLK PE=1 SV=1 | SLK | 0.004276 | 202.8028 |
| 25 | E1BEH7 | tr\|E1BEH7\|E1BEH7_BOVIN Tandem C2 domains, nuclear OS=Bos taurus OX=9913 GN=TC2N PE=4 SV=1 | TC2N | 0.004292 | 144.8656 |
| 26 | E1BFB9 | tr\|E1BFB9\|E1BFB9_BOVIN Protein FAM114A2 OS=Bos taurus OX=9913 GN=FAM114A1 PE=3 SV=2;tr\|A0A3Q1NHI6\|A0A3Q1NHI6_BOVIN Protein FAM114A2 OS=Bos taurus OX=9913 GN=FAM114A1 PE=3 SV=1 | FAM114 | 0.004317 | -254.317 |
| 27 | F1N269 | tr\|F1N269\|F1N269_BOVIN Myosin light chain kinase, smooth muscle OS=Bos taurus OX=9913 GN=MYLK PE=1 SV=3;sp\|Q28824\|MYLK_BOVIN Myosin light chain kinase, smooth muscle OS=Bos taurus OX=9913 GN=MYLK PE=1 SV=1 | MYLK | 0.004338 | 492.7017 |
| 28 | A0A3Q1MGX4 | tr\|A0A3Q1MGX4\|A0A3Q1MGX4_BOVIN Tyrosine-protein kinase OS=Bos taurus OX=9913 GN=ABL2 PE=3 SV=1;tr\|F1MWH8\|F1MWH8_BOVIN Tyrosine-protein kinase OS=Bos taurus OX=9913 GN=ABL2 PE=3 SV=2 | ABL2 | 0.004363 | 217.4476 |
| 29 | Q865S1 | sp\|Q865S1\|AP3D1_BOVIN AP-3 complex subunit delta-1 OS=Bos taurus OX=9913 GN=AP3D1 PE=1 SV=2;tr\|A0A3Q1MCC2\|A0A3Q1MCC2_BOVIN AP-3 complex subunit delta OS=Bos taurus OX=9913 GN=AP3D1 PE=3 SV=1 | AP3D1 | 0.004385 | 195.5746 |
| 30 | F6RWF1 | tr\|F6RWF1\|F6RWF1_BOVIN Nuclear factor 1 OS=Bos taurus OX=9913 GN=NFIC PE=3 SV=1;tr\|A5D9C4\|A5D9C4_BOVIN Nuclear factor 1 (Fragment) OS=Bos taurus OX=9913 GN=NFIC PE=2 SV=1;tr\|Q4ZJ65\|Q4ZJ65_BOVIN Nuclear factor 1 OS=Bos taurus OX=9913 PE=2 SV=1;tr\|Q0V7M9\|Q0V | NFIC | 0.004421 | 194.9047 |
| 31 | Q9TTE1 | sp\|Q9TTE1\|SPA31_BOVIN Serpin A3-1 OS=Bos taurus OX=9913 GN=SERPINA3-1 PE=1 SV=3;sp\|A2I7M9\|SPA32_BOVIN Serpin A3-2 OS=Bos taurus OX=9913 GN=SERPINA3-2 PE=3 SV=1;tr\|A0A452DJK6\|A0A452DJK6_BOVIN Serpin A3-1 OS=Bos taurus OX=9913 GN=SERPINA3-1 PE=3 SV=1;tr\|A0A0 | SERPIN | 0.004613 | -289.43 |
| 32 | E1BLC8 | tr\|E1BLC8\|E1BLC8_BOVIN Eukaryotic translation initiation factor 4 gamma 3 OS=Bos taurus OX=9913 GN=EIF4G3 PE=3 SV=3;tr\|A0A3Q1NNI9\|A0A3Q1NNI9_BOVIN Eukaryotic translation initiation factor 4 gamma 3 OS=Bos taurus OX=9913 GN=EIF4G3 PE=3 SV=1;tr\|A0A3Q1M1I5\|A0 | EIF4G3 | 0.004836 | 218.0482 |
| 33 | A0A3Q1LWE9 | tr\|A0A3Q1LWE9\|A0A3Q1LWE9_BOVIN Histone deacetylase OS=Bos taurus OX=9913 GN=HDAC4 PE=3 SV=1;tr\|F1MYR0\|F1MYR0_BOVIN Histone deacetylase OS=Bos taurus OX=9913 GN=HDAC4 PE=3 SV=3;tr\|F1MWS5\|F1MWS5_BOVIN Histone deacetylase OS=Bos taurus OX=9913 GN=HDAC9 PE=3 S | HDAC4 | 0.005718 | 359.8166 |
| 34 | Q3T0Q4 | sp\|Q3T0Q4\|NDKB_BOVIN Nucleoside diphosphate kinase B OS=Bos taurus OX=9913 GN=NME2 PE=1 SV=1;sp\|P52175\|NDKA2_BOVIN Nucleoside diphosphate kinase A 2 OS=Bos taurus OX=9913 GN=NME1-2 PE=1 SV=3;sp\|P52174\|NDKA1_BOVIN Nucleoside diphosphate kinase A 1 OS=Bos ta | NME2 | 0.005995 | 217.6212 |
| 35 | A0A3Q1LRQ8 | sp\|Q9GLM4\|TENS1_BOVIN Tensin-1 OS=Bos taurus OX=9913 GN=TNS1 PE=2 SV=1;tr\|A0A1P8NW33\|A0A1P8NW33_BOVIN Tensin 1 OS=Bos taurus OX=9913 GN=TNS1 PE=2 SV=1;tr\|A0A3Q1LRQ8\|A0A3Q1LRQ8_BOVIN Tensin-1 OS=Bos taurus OX=9913 GN=TNS1 PE=3 SV=1;tr\|A0A3Q1MT62\|A0A3Q1MT62_ | TNS1 | 0.006004 | -701.194 |
| 36 | F1MXS1 | tr\|F1MXS1\|F1MXS1_BOVIN E3 SUMO-protein ligase RanBP2 OS=Bos taurus OX=9913 GN=RANBP2 PE=4 SV=1;tr\|A0A3Q1LLS4\|A0A3Q1LLS4_BOVIN E3 SUMO-protein ligase RanBP2 OS=Bos taurus OX=9913 GN=RANBP2 PE=4 SV=1;sp\|P48820\|RBP2_BOVIN E3 SUMO-protein ligase RanBP2 (Fragme | RANBP2 | 0.006012 | 545.2737 |
| 37 | P12763 | sp\|P12763\|FETUA_BOVIN Alpha-2-HS-glycoprotein OS=Bos taurus OX=9913 GN=AHSG PE=1 SV=2;tr\|B0JYN6\|B0JYN6_BOVIN Alpha-2-HS-glycoprotein OS=Bos taurus OX=9913 GN=AHSG PE=2 SV=1 | AHSG | 0.006041 | 3.498954 |
| 38 | P12763 | sp\|P12763\|FETUA_BOVIN Alpha-2-HS-glycoprotein OS=Bos taurus OX=9913 GN=AHSG PE=1 SV=2;tr\|B0JYN6\|B0JYN6_BOVIN Alpha-2-HS-glycoprotein OS=Bos taurus OX=9913 GN=AHSG PE=2 SV=1 | AHSG | 0.006623 | 2.8483 |
| 39 | A0A3Q1M6W0 | tr\|A0A3Q1M6W0\|A0A3Q1M6W0_BOVIN Nuclear receptor corepressor 2 OS=Bos taurus OX=9913 GN=NCOR2 PE=3 SV=1;tr\|A0A3Q1MCC9\|A0A3Q1MCC9_BOVIN Nuclear receptor corepressor 2 OS=Bos taurus OX=9913 GN=NCOR2 PE=3 SV=1;tr\|G3X7K5\|G3X7K5_BOVIN Nuclear receptor corepresso | NCOR2 | 0.006699 | 369.5763 |
| 40 | G3MXY7 | tr\|G3MXY7\|G3MXY7_BOVIN Uncharacterized protein OS=Bos taurus OX=9913 PE=4 SV=2;tr\|G3MY42\|G3MY42_BOVIN Uncharacterized protein OS=Bos taurus OX=9913 PE=4 SV=2;tr\|A0A3Q1MEP3\|A0A3Q1MEP3_BOVIN Uncharacterized protein OS=Bos taurus OX=9913 PE=4 SV=1;tr\|A0A3Q1MK | #VALUE! | 0.006942 | 197.7287 |
| 41 | A0A3Q1MJX0 | tr\|A0A3Q1MJX0\|A0A3Q1MJX0_BOVIN Uncharacterized protein OS=Bos taurus OX=9913 GN=HRCT1 PE=4 SV=1 | HRCT1 | 0.011164 | 274.0696 |
| 42 | F1MYG5 | tr\|F1MYG5\|F1MYG5_BOVIN Lamin A/C OS=Bos taurus OX=9913 GN=LMNA PE=1 SV=1;tr\|Q3SZI2\|Q3SZI2_BOVIN Lamin A/C OS=Bos taurus OX=9913 GN=LMNA PE=2 SV=1 | LMNA | 0.011892 | 4.565126 |
| 43 | F1MCK2 | tr\|F1MCK2\|F1MCK2_BOVIN AHNAK nucleoprotein OS=Bos taurus OX=9913 GN=AHNAK PE=1 SV=3;tr\|A0A3Q1NE82\|A0A3Q1NE82_BOVIN AHNAK nucleoprotein OS=Bos taurus OX=9913 GN=AHNAK PE=1 SV=1 | AHNAK | 0.018009 | 3.206035 |
| 44 | P80195 | sp\|P80195\|GLCM1_BOVIN Glycosylation-dependent cell adhesion molecule 1 OS=Bos taurus OX=9913 GN=GLYCAM1 PE=1 SV=2;tr\|A0A6F8Z1X1\|A0A6F8Z1X1_BOVIN Glycosylation-dependent cell adhesion molecule 1 OS=Bos taurus OX=9913 GN=GlyCam1 PE=2 SV=1 | GLYCAM | 0.025859 | 132.4656 |
| 45 | F2Z4I4 | tr\|F2Z4I4\|F2Z4I4_BOVIN Cardiac phospholamban OS=Bos taurus OX=9913 GN=PLN PE=3 SV=1;sp\|A4IFH6\|PPLA_BOVIN Cardiac phospholamban OS=Bos taurus OX=9913 GN=PLN PE=1 SV=1 | PLN | 0.029383 | 99.99005 |
| 46 | A0A3Q1MR36 | tr\|A0A3Q1MR36\|A0A3Q1MR36_BOVIN Anion exchange protein OS=Bos taurus OX=9913 GN=SLC4A1 PE=3 SV=1;tr\|Q9XSW5\|Q9XSW5_BOVIN Anion exchange protein OS=Bos taurus OX=9913 GN=SLC4A1 PE=2 SV=1;tr\|F6QPG2\|F6QPG2_BOVIN Anion exchange protein OS=Bos taurus OX=9913 GN=S | SLC4A1 | 0.029436 | 185.4862 |
| 47 | A0A3Q1MTY6 | tr\|A0A3Q1MTY6\|A0A3Q1MTY6_BOVIN Microtubule associated protein 1B OS=Bos taurus OX=9913 GN=MAP1B PE=4 SV=1;tr\|F1N1S2\|F1N1S2_BOVIN Microtubule associated protein 1B OS=Bos taurus OX=9913 GN=MAP1B PE=4 SV=3;tr\|Q148E3\|Q148E3_BOVIN MAP1B protein (Fragment) OS=B | MAP1B | 0.030182 | -182.754 |
| 48 | F1MCK2 | tr\|F1MCK2\|F1MCK2_BOVIN AHNAK nucleoprotein OS=Bos taurus OX=9913 GN=AHNAK PE=1 SV=3;tr\|A0A3Q1NE82\|A0A3Q1NE82_BOVIN AHNAK nucleoprotein OS=Bos taurus OX=9913 GN=AHNAK PE=1 SV=1;tr\|A0A3Q1MHT0\|A0A3Q1MHT0_BOVIN AHNAK nucleoprotein OS=Bos taurus OX=9913 GN=AHNA | AHNAK | 0.03113 | 143.3969 |
| 49 | A0A3Q1ME14 | tr\|A0A3Q1ME14\|A0A3Q1ME14_BOVIN Nucleosome assembly protein 1-like 4 OS=Bos taurus OX=9913 GN=NAP1L4 PE=3 SV=1;sp\|Q2TA40\|NP1L4_BOVIN Nucleosome assembly protein 1-like 4 OS=Bos taurus OX=9913 GN=NAP1L4 PE=2 SV=1;tr\|A0A3Q1M1N7\|A0A3Q1M1N7_BOVIN Nucleosome ass | NAP1L4 | 0.031472 | 273.7757 |
| 50 | Q0VCZ5 | tr\|Q0VCZ5\|Q0VCZ5_BOVIN Protein phosphatase 1 regulatory subunit 13 like OS=Bos taurus OX=9913 GN=PPP1R13L PE=1 SV=1;tr\|A0A3Q1M2S9\|A0A3Q1M2S9_BOVIN Protein phosphatase 1 regulatory subunit 13 like OS=Bos taurus OX=9913 GN=PPP1R13L PE=1 SV=1 | PPP1R1 | 0.032267 | 43.84628 |
| 51 | CON__P02662 | sp\|P02662\|CASA1_BOVIN Alpha-S1-casein OS=Bos taurus OX=9913 GN=CSN1S1 PE=1 SV=2;tr\|B5B3R8\|B5B3R8_BOVIN Alpha-S1-casein OS=Bos taurus OX=9913 GN=CSN1S1 PE=2 SV=1 | CSN1S1 | 0.032395 | -3.74551 |
| 52 | E1BHT5 | tr\|E1BHT5\|E1BHT5_BOVIN Ubiquitin protein ligase E3 component n-recognin 4 OS=Bos taurus OX=9913 GN=UBR4 PE=3 SV=2 | UBR4 | 0.032449 | 195.8527 |
| 53 | A0A3Q1LPN3 | tr\|A0A3Q1LPN3\|A0A3Q1LPN3_BOVIN SAM and SH3 domain containing 1 OS=Bos taurus OX=9913 GN=SASH1 PE=4 SV=1;tr\|F1N4B0\|F1N4B0_BOVIN SAM and SH3 domain containing 1 OS=Bos taurus OX=9913 GN=SASH1 PE=4 SV=2 | SASH1 | 0.032522 | 57.96175 |
| 54 | E1BHK2 | tr\|E1BHK2\|E1BHK2_BOVIN Adducin 1 OS=Bos taurus OX=9913 GN=ADD1 PE=3 SV=2 | ADD1 | 0.033994 | 304.5612 |
| 55 | Q0VCA5 | sp\|Q0VCA5\|SAMH1_BOVIN Deoxynucleoside triphosphate triphosphohydrolase SAMHD1 OS=Bos taurus OX=9913 GN=SAMHD1 PE=2 SV=1 | SAMHD1 | 0.034722 | 102.3059 |
| 56 | P12763 | sp\|P12763\|FETUA_BOVIN Alpha-2-HS-glycoprotein OS=Bos taurus OX=9913 GN=AHSG PE=1 SV=2;tr\|B0JYN6\|B0JYN6_BOVIN Alpha-2-HS-glycoprotein OS=Bos taurus OX=9913 GN=AHSG PE=2 SV=1 | AHSG | 0.035246 | 2.532432 |
| 57 | A4PJ02 | tr\|A4PJ02\|A4PJ02_BOVIN Glycophorin A OS=Bos taurus OX=9913 GN=gpa PE=2 SV=1 | gpa | 0.036685 | -227.962 |
| 58 | A5D9H5 | tr\|A5D9H5\|A5D9H5_BOVIN Heterogeneous nuclear ribonucleoprotein D OS=Bos taurus OX=9913 GN=HNRNPD PE=1 SV=1;tr\|A6H6Y0\|A6H6Y0_BOVIN HNRNPD protein OS=Bos taurus OX=9913 GN=HNRNPD PE=1 SV=1;tr\|A0A3Q1M8I4\|A0A3Q1M8I4_BOVIN Heterogeneous nuclear ribonucleoprotei | HNRNPD | 0.037106 | 67.21156 |
| 59 | P01966 | sp\|P01966\|HBA_BOVIN Hemoglobin subunit alpha OS=Bos taurus OX=9913 GN=HBA PE=1 SV=2;tr\|A0A1K0FUD3\|A0A1K0FUD3_BOVIN Globin C1 OS=Bos taurus OX=9913 GN=GLNC1 PE=3 SV=1;tr\|A0A452DIQ5\|A0A452DIQ5_BOVIN GLOBIN domain-containing protein OS=Bos taurus OX=9913 GN=H | HBA | 0.037133 | -60.8754 |
| 60 | A4IFK4 | tr\|A4IFK4\|A4IFK4_BOVIN SYNPO2 protein OS=Bos taurus OX=9913 GN=SYNPO2 PE=1 SV=1 | SYNPO2 | 0.037556 | 115.8565 |
| 61 | F1N169 | tr\|F1N169\|F1N169_BOVIN Filamin A OS=Bos taurus OX=9913 GN=FLNA PE=1 SV=3;tr\|A0A3Q1M057\|A0A3Q1M057_BOVIN Filamin A OS=Bos taurus OX=9913 GN=FLNA PE=1 SV=1;tr\|A0A3Q1LMA3\|A0A3Q1LMA3_BOVIN Filamin A OS=Bos taurus OX=9913 GN=FLNA PE=1 SV=1;tr\|Q8WMQ6\|Q8WMQ6_BOVI | FLNA | 0.038073 | -80.1341 |
| 62 | Q1RMR9 | tr\|Q1RMR9\|Q1RMR9_BOVIN Protein kinase C and casein kinase substrate in neurons 2 OS=Bos taurus OX=9913 GN=PACSIN2 PE=2 SV=1 | PACSIN | 0.038177 | 197.7637 |
| 63 | A0A3Q1LVW2 | tr\|A0A3Q1LVW2\|A0A3Q1LVW2_BOVIN SWI/SNF related, matrix associated, actin dependent regulator of chromatin subfamily c member 2 OS=Bos taurus OX=9913 GN=SMARCC2 PE=4 SV=1;tr\|F1N4N6\|F1N4N6_BOVIN SWI/SNF related, matrix associated, actin dependent regulator o | SMARCC | 0.038505 | -97.4551 |
| 64 | G5E507 | tr\|G5E507\|G5E507_BOVIN Heat shock protein HSP 90-beta OS=Bos taurus OX=9913 GN=HSP90AB1 PE=1 SV=2;sp\|Q76LV1\|HS90B_BOVIN Heat shock protein HSP 90-beta OS=Bos taurus OX=9913 GN=HSP90AB1 PE=2 SV=3;tr\|Q3SZD6\|Q3SZD6_BOVIN HSP90AB1 protein (Fragment) OS=Bos tau | HSP90A | 0.038529 | 69.33629 |
| 65 | F1MYG5 | tr\|F1MYG5\|F1MYG5_BOVIN Lamin A/C OS=Bos taurus OX=9913 GN=LMNA PE=1 SV=1;tr\|Q3SZI2\|Q3SZI2_BOVIN Lamin A/C OS=Bos taurus OX=9913 GN=LMNA PE=2 SV=1 | LMNA | 0.038662 | 59.52933 |
| 66 | E1BLG8 | tr\|E1BLG8\|E1BLG8_BOVIN AMP deaminase OS=Bos taurus OX=9913 GN=AMPD2 PE=3 SV=2;tr\|A0A3Q1M723\|A0A3Q1M723_BOVIN AMP deaminase OS=Bos taurus OX=9913 GN=AMPD2 PE=3 SV=1 | AMPD2 | 0.038761 | 143.7513 |
| 67 | A2VE02 | sp\|A2VE02\|JCAD_BOVIN Junctional protein associated with coronary artery disease homolog OS=Bos taurus OX=9913 GN=JCAD PE=2 SV=1 | JCAD | 0.039675 | -107.447 |
| 68 | Q58CS7 | sp\|Q58CS7\|CHMP3_BOVIN Charged multivesicular body protein 3 OS=Bos taurus OX=9913 GN=CHMP3 PE=2 SV=4;tr\|A0A3Q1MCF4\|A0A3Q1MCF4_BOVIN Charged multivesicular body protein 3 OS=Bos taurus OX=9913 GN=CHMP3 PE=3 SV=1 | CHMP3 | 0.039835 | 106.1013 |
| 69 | Q8HYY3 | tr\|Q8HYY3\|Q8HYY3_BOVIN L-caldesmon OS=Bos taurus OX=9913 PE=2 SV=1;tr\|F1MLW0\|F1MLW0_BOVIN Non-muscle caldesmon OS=Bos taurus OX=9913 GN=CALD1 PE=1 SV=2;tr\|A0A3Q1LTX4\|A0A3Q1LTX4_BOVIN Non-muscle caldesmon OS=Bos taurus OX=9913 GN=CALD1 PE=1 SV=1;tr\|Q32L20\|Q | CALD1 | 0.039969 | 54.67254 |
| 70 | A0A3Q1N1R3 | tr\|A0A3Q1N1R3\|A0A3Q1N1R3_BOVIN RAB11 family interacting protein 1 OS=Bos taurus OX=9913 GN=RAB11FIP1 PE=4 SV=1;tr\|E1BKQ2\|E1BKQ2_BOVIN RAB11 family interacting protein 1 OS=Bos taurus OX=9913 GN=RAB11FIP1 PE=4 SV=2 | RAB11F | 0.039999 | 132.2647 |
| 71 | F1N565 | tr\|F1N565\|F1N565_BOVIN Zinc finger CCCH domain-containing protein 14 OS=Bos taurus OX=9913 GN=ZC3H14 PE=3 SV=3;tr\|F1N567\|F1N567_BOVIN Zinc finger CCCH domain-containing protein 14 OS=Bos taurus OX=9913 GN=ZC3H14 PE=3 SV=3;sp\|Q3ZC82\|ZC3HE_BOVIN Zinc finger | ZC3H14 | 0.040073 | 52.17286 |
| 72 | G3N1L5 | tr\|Q2YDP5\|Q2YDP5_BOVIN Solute carrier family 9 (Sodium/hydrogen exchanger), member 3 regulator 2 OS=Bos taurus OX=9913 GN=SLC9A3R2 PE=2 SV=1;tr\|M5FK54\|M5FK54_BOVIN Solute carrier family 9 isoform 3 regulator 2 OS=Bos taurus OX=9913 GN=SLC9A3R2 PE=4 SV=1;tr | SLC9A3 | 0.04008 | 54.50826 |
| 73 | A0A3Q1M1X1 | tr\|A0A3Q1M1X1\|A0A3Q1M1X1_BOVIN Paxillin OS=Bos taurus OX=9913 GN=PXN PE=1 SV=1;tr\|F1MFD1\|F1MFD1_BOVIN Paxillin OS=Bos taurus OX=9913 GN=PXN PE=1 SV=3 | PXN | 0.040121 | 55.71107 |
| 74 | A1L5B6 | tr\|A1L5B6\|A1L5B6_BOVIN Annexin OS=Bos taurus OX=9913 GN=ANXA5 PE=2 SV=1 | ANXA5 | 0.040168 | 122.0701 |
| 75 | P80724 | sp\|P80724\|BASP1_BOVIN Brain acid soluble protein 1 OS=Bos taurus OX=9913 GN=BASP1 PE=1 SV=3 | BASP1 | 0.040177 | 67.98424 |
| 76 | E1B762 | tr\|E1B762\|E1B762_BOVIN Intercellular adhesion molecule 4 OS=Bos taurus OX=9913 GN=ICAM4 PE=3 SV=1 | ICAM4 | 0.040187 | -91.9923 |
| 77 | F6PXE3 | tr\|F6PXE3\|F6PXE3_BOVIN Caveolae associated protein 2 OS=Bos taurus OX=9913 GN=CAVIN2 PE=1 SV=1;tr\|Q17QZ6\|Q17QZ6_BOVIN SDPR protein (Fragment) OS=Bos taurus OX=9913 GN=SDPR PE=2 SV=1 | CAVIN2 | 0.040205 | -131.722 |
| 78 | A0A3Q1MAM1 | tr\|A0A3Q1MAM1\|A0A3Q1MAM1_BOVIN Uncharacterized protein OS=Bos taurus OX=9913 PE=3 SV=1;sp\|F1N5S9\|FUND1_BOVIN FUN14 domain-containing protein 1 OS=Bos taurus OX=9913 GN=FUNDC1 PE=2 SV=1 | FUNDC1 | 0.040253 | 113.0597 |
| 79 | F1MV32 | tr\|F1MV32\|F1MV32_BOVIN Adhesion G protein-coupled receptor F5 OS=Bos taurus OX=9913 GN=ADGRF5 PE=3 SV=2 | ADGRF5 | 0.040283 | 90.79064 |
| 80 | E1BIX5 | tr\|E1BIX5\|E1BIX5_BOVIN Nucleoporin 153 OS=Bos taurus OX=9913 GN=NUP153 PE=1 SV=3;tr\|A0A3Q1MAS6\|A0A3Q1MAS6_BOVIN Nucleoporin 153 OS=Bos taurus OX=9913 GN=NUP153 PE=1 SV=1 | NUP153 | 0.040344 | -43.5503 |
| 81 | F6QH94 | tr\|F6QH94\|F6QH94_BOVIN Protein disulfide-isomerase A6 OS=Bos taurus OX=9913 GN=PDIA6 PE=1 SV=1;tr\|A6QNL5\|A6QNL5_BOVIN Protein disulfide-isomerase A6 (Fragment) OS=Bos taurus OX=9913 GN=PDIA6 PE=2 SV=1 | PDIA6 | 0.040362 | 40.61689 |
| 82 | A0A3Q1MSN5 | tr\|A0A3Q1MSN5\|A0A3Q1MSN5_BOVIN Phosphoinositide 5-phosphatase OS=Bos taurus OX=9913 GN=SYNJ1 PE=3 SV=1;tr\|E1BD68\|E1BD68_BOVIN Phosphoinositide 5-phosphatase OS=Bos taurus OX=9913 GN=SYNJ1 PE=3 SV=2;tr\|A0A3Q1MIH5\|A0A3Q1MIH5_BOVIN Phosphoinositide 5-phosphat | SYNJ1 | 0.040367 | 102.5338 |
| 83 | A8E644 | tr\|A8E644\|A8E644_BOVIN DCLK1 protein OS=Bos taurus OX=9913 GN=DCLK1 PE=2 SV=1;tr\|F1MDA8\|F1MDA8_BOVIN Protein kinase domain-containing protein OS=Bos taurus OX=9913 GN=DCLK1 PE=3 SV=3 | DCLK1 | 0.040395 | 40.85895 |
| 84 | A0A3Q1LQV4 | tr\|A0A3Q1LQV4\|A0A3Q1LQV4_BOVIN Tetratricopeptide repeat protein 4 OS=Bos taurus OX=9913 GN=TTC4 PE=3 SV=1 | TTC4 | 0.040411 | -61.4015 |
| 85 | A0A3Q1MTY6 | tr\|A0A3Q1MTY6\|A0A3Q1MTY6_BOVIN Microtubule associated protein 1B OS=Bos taurus OX=9913 GN=MAP1B PE=4 SV=1;tr\|F1N1S2\|F1N1S2_BOVIN Microtubule associated protein 1B OS=Bos taurus OX=9913 GN=MAP1B PE=4 SV=3 | MAP1B | 0.040412 | -100.221 |
| 86 | E1BEH7 | tr\|E1BEH7\|E1BEH7_BOVIN Tandem C2 domains, nuclear OS=Bos taurus OX=9913 GN=TC2N PE=4 SV=1 | TC2N | 0.040435 | 43.07315 |
| 87 | A7Z032 | tr\|A7Z032\|A7Z032_BOVIN M-RIP protein OS=Bos taurus OX=9913 GN=M-RIP PE=2 SV=1;tr\|A0A3Q1NK54\|A0A3Q1NK54_BOVIN Myosin phosphatase Rho interacting protein OS=Bos taurus OX=9913 GN=MPRIP PE=4 SV=1;tr\|F1MNF9\|F1MNF9_BOVIN Myosin phosphatase Rho interacting prote | M-RIP | 0.040466 | 122.6136 |
| 88 | O77834 | sp\|O77834\|PRDX6_BOVIN Peroxiredoxin-6 OS=Bos taurus OX=9913 GN=PRDX6 PE=1 SV=3 | PRDX6 | 0.040513 | 68.55715 |
| 89 | Q2NKU4 | tr\|Q2NKU4\|Q2NKU4_BOVIN Calcium regulated heat stable protein 1, 24kDa OS=Bos taurus OX=9913 GN=CARHSP1 PE=1 SV=1;tr\|M5FKD4\|M5FKD4_BOVIN Calcium regulated heat stable protein 1, 24kDa OS=Bos taurus OX=9913 GN=CARHSP1 PE=4 SV=1 | CARHSP | 0.040531 | 121.3962 |
| 90 | A1A4J1 | sp\|A1A4J1\|PFKAL_BOVIN ATP-dependent 6-phosphofructokinase, liver type OS=Bos taurus OX=9913 GN=PFKL PE=2 SV=1 | PFKL | 0.040575 | 60.36511 |
| 91 | P01966 | sp\|P01966\|HBA_BOVIN Hemoglobin subunit alpha OS=Bos taurus OX=9913 GN=HBA PE=1 SV=2;tr\|A0A1K0FUD3\|A0A1K0FUD3_BOVIN Globin C1 OS=Bos taurus OX=9913 GN=GLNC1 PE=3 SV=1 | HBA | 0.040593 | 121.0886 |
| 92 | E1BH45 | tr\|E1BH45\|E1BH45_BOVIN RB1 inducible coiled-coil 1 OS=Bos taurus OX=9913 GN=RB1CC1 PE=4 SV=1 | RB1CC1 | 0.040593 | -64.1128 |
| 93 | F1N4U5 | tr\|F1N4U5\|F1N4U5_BOVIN G-patch domain containing 3 OS=Bos taurus OX=9913 GN=GPATCH3 PE=4 SV=1;tr\|A5PKC9\|A5PKC9_BOVIN GPATCH3 protein OS=Bos taurus OX=9913 GN=GPATCH3 PE=2 SV=1 | GPATCH | 0.04061 | 932.9322 |
| 94 | Q9BDK2 | sp\|Q9BDK2\|AIF1_BOVIN Allograft inflammatory factor 1 OS=Bos taurus OX=9913 GN=AIF1 PE=2 SV=1 | AIF1 | 0.040636 | -47.8859 |
| 95 | E1BND0 | tr\|E1BND0\|E1BND0_BOVIN Ubiquitinyl hydrolase 1 OS=Bos taurus OX=9913 GN=USP24 PE=3 SV=1;tr\|A0A3Q1M1T8\|A0A3Q1M1T8_BOVIN Ubiquitinyl hydrolase 1 OS=Bos taurus OX=9913 GN=USP24 PE=3 SV=1 | USP24 | 0.040645 | 81.43581 |
| 96 | G3N3P0 | tr\|G3N3P0\|G3N3P0_BOVIN BICD cargo adaptor 2 OS=Bos taurus OX=9913 GN=BICD2 PE=3 SV=2 | BICD2 | 0.040674 | 111.5119 |
| 97 | G3N214 | tr\|G3N214\|G3N214_BOVIN Protein phosphatase 6 regulatory subunit 1 OS=Bos taurus OX=9913 GN=PPP6R1 PE=3 SV=2 | PPP6R1 | 0.040704 | 68.10668 |
| 98 | Q3T174 | sp\|Q3T174\|TAF12_BOVIN Transcription initiation factor TFIID subunit 12 OS=Bos taurus OX=9913 GN=TAF12 PE=2 SV=1 | TAF12 | 0.04071 | 47.21591 |
| 99 | F1N7C1 | tr\|F1N7C1\|F1N7C1_BOVIN HECT and RLD domain containing E3 ubiquitin protein ligase family member 6 OS=Bos taurus OX=9913 GN=HERC6 PE=4 SV=3 | HERC6 | 0.040768 | 57.03512 |
| 100 | A7YWH2 | tr\|A7YWH2\|A7YWH2_BOVIN Zinc finger Ran-binding domain-containing protein 2 OS=Bos taurus OX=9913 GN=ZRANB2 PE=2 SV=1 | ZRANB2 | 0.040773 | 55.94077 |
| 101 | E1BI26 | tr\|E1BI26\|E1BI26_BOVIN Mediator of DNA damage checkpoint protein 1 OS=Bos taurus OX=9913 GN=MDC1 PE=4 SV=3 | MDC1 | 0.040784 | 59.87123 |
| 102 | E1BH40 | tr\|E1BH40\|E1BH40_BOVIN RCR-type E3 ubiquitin transferase OS=Bos taurus OX=9913 GN=MYCBP2 PE=3 SV=3 | MYCBP2 | 0.040809 | 52.22687 |
| 103 | F6PXE3 | tr\|F6PXE3\|F6PXE3_BOVIN Caveolae associated protein 2 OS=Bos taurus OX=9913 GN=CAVIN2 PE=1 SV=1;tr\|Q17QZ6\|Q17QZ6_BOVIN SDPR protein (Fragment) OS=Bos taurus OX=9913 GN=SDPR PE=2 SV=1 | CAVIN2 | 0.040811 | 226.4013 |
| 104 | A7Z082 | tr\|A7Z082\|A7Z082_BOVIN ACIN1 protein OS=Bos taurus OX=9913 GN=ACIN1 PE=2 SV=1 | ACIN1 | 0.040816 | -30.0557 |
| 105 | F1MPF7 | tr\|F1MPF7\|F1MPF7_BOVIN Pleckstrin homology domain interacting protein OS=Bos taurus OX=9913 GN=PHIP PE=4 SV=2;tr\|A0A3Q1LLQ0\|A0A3Q1LLQ0_BOVIN Pleckstrin homology domain interacting protein OS=Bos taurus OX=9913 GN=PHIP PE=4 SV=1 | PHIP | 0.040839 | 72.97976 |
| 106 | A0A3Q1MBY4 | tr\|A0A3Q1MBY4\|A0A3Q1MBY4_BOVIN cAMP-dependent protein kinase type I-alpha regulatory subunit OS=Bos taurus OX=9913 GN=PRKAR1A PE=3 SV=1;sp\|P00514\|KAP0_BOVIN cAMP-dependent protein kinase type I-alpha regulatory subunit OS=Bos taurus OX=9913 GN=PRKAR1A PE=1 | PRKAR1 | 0.04088 | 42.91624 |
| 107 | E1BGB0 | tr\|E1BGB0\|E1BGB0_BOVIN Kinesin family member 13B OS=Bos taurus OX=9913 GN=KIF13B PE=3 SV=3;tr\|A0A3Q1NM85\|A0A3Q1NM85_BOVIN Kinesin family member 13B OS=Bos taurus OX=9913 GN=KIF13B PE=3 SV=1;tr\|A0A3Q1M799\|A0A3Q1M799_BOVIN Kinesin family member 13B OS=Bos ta | KIF13B | 0.040897 | -109.59 |
| 108 | A0A3Q1M831 | tr\|A0A3Q1M831\|A0A3Q1M831_BOVIN Jumonji domain containing 1C OS=Bos taurus OX=9913 GN=JMJD1C PE=4 SV=1;tr\|F1N685\|F1N685_BOVIN Jumonji domain containing 1C OS=Bos taurus OX=9913 GN=JMJD1C PE=4 SV=2 | JMJD1C | 0.040973 | -36.0779 |
| 109 | E1BP14 | tr\|E1BP14\|E1BP14_BOVIN Rho guanine nucleotide exchange factor 17 OS=Bos taurus OX=9913 GN=ARHGEF17 PE=4 SV=3;tr\|A0A3Q1MT41\|A0A3Q1MT41_BOVIN Rho guanine nucleotide exchange factor 17 OS=Bos taurus OX=9913 GN=ARHGEF17 PE=4 SV=1 | ARHGEF | 0.040994 | -135.103 |
| 110 | E1BBR6 | tr\|E1BBR6\|E1BBR6_BOVIN FYVE and coiled-coil domain autophagy adaptor 1 OS=Bos taurus OX=9913 GN=FYCO1 PE=4 SV=2 | FYCO1 | 0.041001 | 91.77889 |
| 111 | A0A3Q1NCL2 | tr\|A0A3Q1NCL2\|A0A3Q1NCL2_BOVIN Uncharacterized protein OS=Bos taurus OX=9913 PE=4 SV=1 | #VALUE! | 0.04109 | -28.5679 |
| 112 | Q2KJA9 | tr\|Q2KJA9\|Q2KJA9_BOVIN Endothelial cell adhesion molecule OS=Bos taurus OX=9913 GN=ESAM PE=2 SV=1 | ESAM | 0.041097 | 68.77591 |
| 113 | E1BC91 | tr\|A0A3Q1MKV4\|A0A3Q1MKV4_BOVIN LIM and calponin homology domains 1 OS=Bos taurus OX=9913 GN=LIMCH1 PE=4 SV=1;tr\|A0A3Q1M0L9\|A0A3Q1M0L9_BOVIN LIM and calponin homology domains 1 OS=Bos taurus OX=9913 GN=LIMCH1 PE=4 SV=1;tr\|A0A3Q1MRR5\|A0A3Q1MRR5_BOVIN LIM and | LIMCH1 | 0.041113 | -35.2586 |
| 114 | F1MTG3 | tr\|F1MTG3\|F1MTG3_BOVIN CAP-ZIP_m domain-containing protein OS=Bos taurus OX=9913 GN=FAM21A PE=4 SV=2 | FAM21A | 0.04115 | 69.99856 |
| 115 | Q08DI3 | tr\|Q08DI3\|Q08DI3_BOVIN Sperm associated antigen 9 OS=Bos taurus OX=9913 GN=SPAG9 PE=2 SV=1;tr\|F1MZ69\|F1MZ69_BOVIN Sperm associated antigen 9 OS=Bos taurus OX=9913 GN=SPAG9 PE=4 SV=2;tr\|A0A3Q1LP94\|A0A3Q1LP94_BOVIN Sperm associated antigen 9 OS=Bos taurus OX | SPAG9 | 0.041252 | -64.0857 |
| 116 | A0A3Q1N4I6 | tr\|A0A3Q1N4I6\|A0A3Q1N4I6_BOVIN Teneurin transmembrane protein 4 OS=Bos taurus OX=9913 GN=TENM4 PE=3 SV=1;tr\|A0A3Q1N0V2\|A0A3Q1N0V2_BOVIN Teneurin transmembrane protein 4 OS=Bos taurus OX=9913 GN=TENM4 PE=3 SV=1;tr\|A0A3Q1M3M4\|A0A3Q1M3M4_BOVIN Teneurin transm | TENM4 | 0.041253 | -51.4388 |
| 117 | F1MCK2 | tr\|F1MCK2\|F1MCK2_BOVIN AHNAK nucleoprotein OS=Bos taurus OX=9913 GN=AHNAK PE=1 SV=3;tr\|A0A3Q1NE82\|A0A3Q1NE82_BOVIN AHNAK nucleoprotein OS=Bos taurus OX=9913 GN=AHNAK PE=1 SV=1;tr\|A0A3Q1MHT0\|A0A3Q1MHT0_BOVIN AHNAK nucleoprotein OS=Bos taurus OX=9913 GN=AHNA | AHNAK | 0.041291 | -43.1488 |
| 118 | A0A3Q1MT17 | tr\|A0A3Q1MT17\|A0A3Q1MT17_BOVIN Plakophilin 4 OS=Bos taurus OX=9913 GN=PKP4 PE=3 SV=1;tr\|F1MJ11\|F1MJ11_BOVIN Plakophilin 4 OS=Bos taurus OX=9913 GN=PKP4 PE=3 SV=3 | PKP4 | 0.041316 | 56.79273 |
| 119 | E1B9L5 | tr\|E1B9L5\|E1B9L5_BOVIN FMR1 autosomal homolog 2 OS=Bos taurus OX=9913 GN=FXR2 PE=3 SV=3;tr\|A0A3Q1MDB8\|A0A3Q1MDB8_BOVIN FMR1 autosomal homolog 2 OS=Bos taurus OX=9913 GN=FXR2 PE=3 SV=1 | FXR2 | 0.041324 | 73.8033 |
| 120 | F1MJB0 | tr\|F1MJB0\|F1MJB0_BOVIN Filamin A interacting protein 1 like OS=Bos taurus OX=9913 GN=FILIP1L PE=4 SV=3 | FILIP1 | 0.041379 | -41.1496 |
| 121 | A3KMX4 | tr\|A3KMX4\|A3KMX4_BOVIN REPS1 protein (Fragment) OS=Bos taurus OX=9913 GN=REPS1 PE=2 SV=1;tr\|A0A3Q1LZ35\|A0A3Q1LZ35_BOVIN RALBP1 associated Eps domain containing 1 OS=Bos taurus OX=9913 GN=REPS1 PE=4 SV=1;tr\|F6Q4T4\|F6Q4T4_BOVIN RALBP1 associated Eps domain c | REPS1 | 0.041389 | 82.24987 |
| 122 | F6PXE3 | tr\|F6PXE3\|F6PXE3_BOVIN Caveolae associated protein 2 OS=Bos taurus OX=9913 GN=CAVIN2 PE=1 SV=1;tr\|Q17QZ6\|Q17QZ6_BOVIN SDPR protein (Fragment) OS=Bos taurus OX=9913 GN=SDPR PE=2 SV=1 | CAVIN2 | 0.041431 | -83.8107 |
| 123 | A0A3Q1LPC2 | tr\|A0A3Q1LPC2\|A0A3Q1LPC2_BOVIN Vestigial like family member 4 OS=Bos taurus OX=9913 GN=VGLL4 PE=4 SV=1;tr\|A6H7A5\|A6H7A5_BOVIN VGLL4 protein OS=Bos taurus OX=9913 GN=VGLL4 PE=2 SV=1;tr\|A0A3Q1MTH3\|A0A3Q1MTH3_BOVIN Vestigial like family member 4 OS=Bos taurus | VGLL4 | 0.041465 | -36.0165 |
| 124 | A4IF71 | tr\|A4IF71\|A4IF71_BOVIN PKP2 protein OS=Bos taurus OX=9913 GN=PKP2 PE=2 SV=1;tr\|A0A3Q1M2G3\|A0A3Q1M2G3_BOVIN Plakophilin 2 OS=Bos taurus OX=9913 GN=PKP2 PE=3 SV=1 | PKP2 | 0.04158 | 40.94693 |
| 125 | Q3T186 | sp\|Q3T186\|RPIA_BOVIN Ribose-5-phosphate isomerase (Fragment) OS=Bos taurus OX=9913 GN=RPIA PE=2 SV=2;tr\|G5E534\|G5E534_BOVIN Ribose-5-phosphate isomerase OS=Bos taurus OX=9913 GN=RPIA PE=3 SV=1 | RPIA | 0.041754 | 58.80691 |
| 126 | Q5EA61 | sp\|Q5EA61\|KCRB_BOVIN Creatine kinase B-type OS=Bos taurus OX=9913 GN=CKB PE=1 SV=1 | CKB | 0.041783 | -34.4547 |
| 127 | F1MZY0 | tr\|F1MZY0\|F1MZY0_BOVIN Cdk5 and Abl enzyme substrate 1 OS=Bos taurus OX=9913 GN=CABLES1 PE=4 SV=3;tr\|A0A3Q1MA63\|A0A3Q1MA63_BOVIN Cdk5 and Abl enzyme substrate 1 OS=Bos taurus OX=9913 GN=CABLES1 PE=4 SV=1;tr\|A0A3Q1N5R6\|A0A3Q1N5R6_BOVIN Cdk5 and Abl enzyme s | CABLES | 0.041835 | 42.58646 |
| 128 | A0A3Q1M2A8 | tr\|A0A3Q1M2A8\|A0A3Q1M2A8_BOVIN Complement factor I OS=Bos taurus OX=9913 GN=CFI PE=1 SV=1;tr\|A0A3Q1LGM4\|A0A3Q1LGM4_BOVIN Complement factor I OS=Bos taurus OX=9913 GN=CFI PE=1 SV=1;tr\|F1N4M7\|F1N4M7_BOVIN Complement factor I OS=Bos taurus OX=9913 GN=CFI PE=1 | CFI | 0.041843 | 101.1978 |
| 129 | E1BIJ3 | tr\|E1BIJ3\|E1BIJ3_BOVIN CASK interacting protein 2 OS=Bos taurus OX=9913 GN=CASKIN2 PE=4 SV=3 | CASKIN | 0.041871 | 138.9959 |
| 130 | A0A3Q1MSB2 | tr\|A0A3Q1MSB2\|A0A3Q1MSB2_BOVIN A-kinase anchoring protein 12 OS=Bos taurus OX=9913 GN=AKAP12 PE=1 SV=1;tr\|G3MWT9\|G3MWT9_BOVIN A-kinase anchoring protein 12 OS=Bos taurus OX=9913 GN=AKAP12 PE=1 SV=2 | AKAP12 | 0.041909 | 65.41134 |
| 131 | A0A3Q1M0V1 | tr\|A0A3Q1M0V1\|A0A3Q1M0V1_BOVIN Catenin alpha-1 OS=Bos taurus OX=9913 GN=CTNNA1 PE=3 SV=1;sp\|Q3MHM6\|CTNA1_BOVIN Catenin alpha-1 OS=Bos taurus OX=9913 GN=CTNNA1 PE=2 SV=1;tr\|F1MM34\|F1MM34_BOVIN Catenin alpha-1 OS=Bos taurus OX=9913 GN=CTNNA1 PE=3 SV=2;tr\|A0A | CTNNA1 | 0.041916 | 71.34289 |
| 132 | Q1RMS3 | tr\|Q1RMS3\|Q1RMS3_BOVIN Daxx OS=Bos taurus OX=9913 GN=DAXX PE=2 SV=1;tr\|F1MGU0\|F1MGU0_BOVIN Daxx OS=Bos taurus OX=9913 GN=DAXX PE=3 SV=1 | DAXX | 0.041946 | 71.17913 |
| 133 | A0A3Q1MVZ0 | tr\|A0A3Q1MVZ0\|A0A3Q1MVZ0_BOVIN ANTXR cell adhesion molecule 1 OS=Bos taurus OX=9913 GN=ANTXR1 PE=3 SV=1;tr\|E1BC74\|E1BC74_BOVIN ANTXR cell adhesion molecule 1 OS=Bos taurus OX=9913 GN=ANTXR1 PE=3 SV=3 | ANTXR1 | 0.041983 | 69.03197 |
| 134 | F1N4E5 | sp\|F1N4E5\|TOIP1_BOVIN Torsin-1A-interacting protein 1 OS=Bos taurus OX=9913 GN=TOR1AIP1 PE=3 SV=2;tr\|A0A3Q1LTR8\|A0A3Q1LTR8_BOVIN Torsin 1A interacting protein 1 OS=Bos taurus OX=9913 GN=TOR1AIP1 PE=3 SV=1;tr\|A0A3Q1MJM3\|A0A3Q1MJM3_BOVIN Torsin 1A interactin | TOR1AI | 0.042033 | 112.6774 |
| 135 | A0A3Q1M6X7 | tr\|A0A3Q1M6X7\|A0A3Q1M6X7_BOVIN A-kinase anchoring protein 13 OS=Bos taurus OX=9913 GN=AKAP13 PE=4 SV=1;tr\|A0A3Q1MRE3\|A0A3Q1MRE3_BOVIN A-kinase anchoring protein 13 OS=Bos taurus OX=9913 GN=AKAP13 PE=4 SV=1;tr\|F1MY16\|F1MY16_BOVIN A-kinase anchoring protein | AKAP13 | 0.042057 | 49.68511 |
| 136 | P48616 | sp\|P48616\|VIME_BOVIN Vimentin OS=Bos taurus OX=9913 GN=VIM PE=1 SV=3 | VIM | 0.042239 | -41.2164 |
| 137 | A0A3Q1MFI2 | tr\|A0A3Q1MFI2\|A0A3Q1MFI2_BOVIN Constitutive coactivator of PPAR-gamma-like protein 1 OS=Bos taurus OX=9913 GN=FAM120A PE=3 SV=1;sp\|A6H7H1\|F120A_BOVIN Constitutive coactivator of PPAR-gamma-like protein 1 OS=Bos taurus OX=9913 GN=FAM120A PE=2 SV=1;tr\|F1MUK8 | FAM120 | 0.042485 | 68.54029 |
| 138 | A0A3Q1M528 | tr\|A0A3Q1M528\|A0A3Q1M528_BOVIN RRM domain-containing protein OS=Bos taurus OX=9913 PE=4 SV=1;tr\|A2VE34\|A2VE34_BOVIN PABPN1 protein OS=Bos taurus OX=9913 GN=PABPN1 PE=2 SV=1;tr\|A3KN10\|A3KN10_BOVIN PABPN1 protein OS=Bos taurus OX=9913 GN=PABPN1 PE=2 SV=1;sp\| | PABPN1 | 0.042509 | -54.529 |
| 139 | E1BG86 | tr\|E1BG86\|E1BG86_BOVIN Dishevelled binding antagonist of beta catenin 3 OS=Bos taurus OX=9913 GN=DACT3 PE=3 SV=3 | DACT3 | 0.042572 | 47.52686 |
| 140 | A0A3Q1MBB8 | tr\|A0A3Q1MBB8\|A0A3Q1MBB8_BOVIN Sphingomyelin phosphodiesterase OS=Bos taurus OX=9913 GN=SMPD3 PE=4 SV=1;tr\|E1BFH4\|E1BFH4_BOVIN Sphingomyelin phosphodiesterase OS=Bos taurus OX=9913 GN=SMPD3 PE=4 SV=1 | SMPD3 | 0.042711 | 33.00752 |
| 141 | Q08DI3 | tr\|Q08DI3\|Q08DI3_BOVIN Sperm associated antigen 9 OS=Bos taurus OX=9913 GN=SPAG9 PE=2 SV=1;tr\|F1MZ69\|F1MZ69_BOVIN Sperm associated antigen 9 OS=Bos taurus OX=9913 GN=SPAG9 PE=4 SV=2;tr\|A0A3Q1LP94\|A0A3Q1LP94_BOVIN Sperm associated antigen 9 OS=Bos taurus OX | SPAG9 | 0.042744 | 86.08619 |
| 142 | G3MYP5 | tr\|G3MYP5\|G3MYP5_BOVIN Microtubule associated protein 1A OS=Bos taurus OX=9913 GN=MAP1A PE=4 SV=2;tr\|A0A3Q1M9Z4\|A0A3Q1M9Z4_BOVIN Microtubule associated protein 1A OS=Bos taurus OX=9913 GN=MAP1A PE=4 SV=1 | MAP1A | 0.042787 | -52.7813 |
| 143 | A0A3Q1N8M0 | tr\|A0A3Q1N8M0\|A0A3Q1N8M0_BOVIN Phosphatidylinositol transfer protein membrane associated 2 OS=Bos taurus OX=9913 GN=PITPNM2 PE=3 SV=1;tr\|A0A3Q1MUM3\|A0A3Q1MUM3_BOVIN Phosphatidylinositol transfer protein membrane associated 2 OS=Bos taurus OX=9913 GN=PITPNM | PITPNM | 0.042805 | 40.30275 |
| 144 | A0A140T863 | tr\|A0A140T863\|A0A140T863_BOVIN E3 ubiquitin-protein ligase RNF113A OS=Bos taurus OX=9913 GN=RNF113A PE=4 SV=1;sp\|Q67ER4\|R113A_BOVIN E3 ubiquitin-protein ligase RNF113A OS=Bos taurus OX=9913 GN=RNF113A PE=2 SV=1 | RNF113 | 0.0431 | 98.95277 |
| 145 | G5E6K6 | tr\|G5E6K6\|G5E6K6_BOVIN Formin binding protein 1 OS=Bos taurus OX=9913 GN=FNBP1 PE=4 SV=2;tr\|F1MQ90\|F1MQ90_BOVIN Formin binding protein 1 OS=Bos taurus OX=9913 GN=FNBP1 PE=4 SV=3;tr\|A0A3Q1LPM3\|A0A3Q1LPM3_BOVIN Formin binding protein 1 OS=Bos taurus OX=9913 | FNBP1 | 0.043284 | 43.28078 |
| 146 | Q2HJ49 | sp\|Q2HJ49\|MOES_BOVIN Moesin OS=Bos taurus OX=9913 GN=MSN PE=2 SV=3 | MSN | 0.043343 | 38.49097 |
| 147 | Q3SZ63 | sp\|Q3SZ63\|NOP56_BOVIN Nucleolar protein 56 OS=Bos taurus OX=9913 GN=NOP56 PE=2 SV=1;tr\|F1MW06\|F1MW06_BOVIN Nucleolar protein 56 OS=Bos taurus OX=9913 GN=NOP56 PE=3 SV=2 | NOP56 | 0.043359 | 68.40611 |
| 148 | P02316 | sp\|P02316\|HMGN1_BOVIN Non-histone chromosomal protein HMG-14 OS=Bos taurus OX=9913 GN=HMGN1 PE=1 SV=2 | HMGN1 | 0.043604 | -311.909 |
| 149 | Q3MHR5 | sp\|Q3MHR5\|SRSF2_BOVIN Serine/arginine-rich splicing factor 2 OS=Bos taurus OX=9913 GN=SRSF2 PE=2 SV=3 | SRSF2 | 0.043606 | 24.56525 |
| 150 | A0A3Q1M6F6 | tr\|A0A3Q1M6F6\|A0A3Q1M6F6_BOVIN Rho GTPase-activating protein 10 OS=Bos taurus OX=9913 GN=ARHGAP10 PE=4 SV=1;tr\|A0A3Q1LTD9\|A0A3Q1LTD9_BOVIN Rho GTPase-activating protein 10 OS=Bos taurus OX=9913 GN=ARHGAP10 PE=4 SV=1;tr\|A0A3Q1MRK0\|A0A3Q1MRK0_BOVIN Rho GTPas | ARHGAP | 0.043682 | -71.3296 |
| 151 | E1BHK2 | tr\|E1BHK2\|E1BHK2_BOVIN Adducin 1 OS=Bos taurus OX=9913 GN=ADD1 PE=3 SV=2 | ADD1 | 0.04379 | 50.06577 |
| 152 | F1N269 | tr\|F1N269\|F1N269_BOVIN Myosin light chain kinase, smooth muscle OS=Bos taurus OX=9913 GN=MYLK PE=1 SV=3;sp\|Q28824\|MYLK_BOVIN Myosin light chain kinase, smooth muscle OS=Bos taurus OX=9913 GN=MYLK PE=1 SV=1 | MYLK | 0.044017 | 100.6933 |
| 153 | P12763 | sp\|P12763\|FETUA_BOVIN Alpha-2-HS-glycoprotein OS=Bos taurus OX=9913 GN=AHSG PE=1 SV=2;tr\|B0JYN6\|B0JYN6_BOVIN Alpha-2-HS-glycoprotein OS=Bos taurus OX=9913 GN=AHSG PE=2 SV=1 | AHSG | 0.044306 | 53.69323 |
| 154 | G3X778 | tr\|G3X778\|G3X778_BOVIN Phosphorylase b kinase regulatory subunit OS=Bos taurus OX=9913 GN=PHKA1 PE=3 SV=2 | PHKA1 | 0.044393 | 75.61502 |
| 155 | F1N0J2 | tr\|F1N0J2\|F1N0J2_BOVIN Microtubule-associated protein OS=Bos taurus OX=9913 GN=MAP4 PE=4 SV=3;sp\|P36225\|MAP4_BOVIN Microtubule-associated protein 4 OS=Bos taurus OX=9913 GN=MAP4 PE=1 SV=1;tr\|F1MAZ3\|F1MAZ3_BOVIN Microtubule-associated protein OS=Bos taurus | MAP4 | 0.044426 | -133.126 |
| 156 | A0A140T8A5 | tr\|A0A140T8A5\|A0A140T8A5_BOVIN Isocitrate dehydrogenase [NADP] OS=Bos taurus OX=9913 GN=IDH1 PE=1 SV=1;sp\|Q9XSG3\|IDHC_BOVIN Isocitrate dehydrogenase [NADP] cytoplasmic OS=Bos taurus OX=9913 GN=IDH1 PE=2 SV=1;tr\|Q0QEQ4\|Q0QEQ4_BOVIN IDP (Fragment) OS=Bos tau | IDH1 | 0.044557 | -33.6562 |
| 157 | A0A3Q1N8T2 | tr\|A0A3Q1N8T2\|A0A3Q1N8T2_BOVIN Proline rich 12 OS=Bos taurus OX=9913 GN=PRR12 PE=4 SV=1;tr\|A0A3Q1ME27\|A0A3Q1ME27_BOVIN Proline rich 12 OS=Bos taurus OX=9913 GN=PRR12 PE=4 SV=1;tr\|F1MSK1\|F1MSK1_BOVIN Proline rich 12 OS=Bos taurus OX=9913 GN=PRR12 PE=4 SV=3 | PRR12 | 0.045505 | -47.0726 |
| 158 | F1CYZ1 | tr\|F1CYZ1\|F1CYZ1_BOVIN Myocyte enhancer factor 2D OS=Bos taurus OX=9913 GN=MEF2D PE=2 SV=1;tr\|A0A3Q1NNL3\|A0A3Q1NNL3_BOVIN Myocyte enhancer factor 2D OS=Bos taurus OX=9913 GN=MEF2D PE=4 SV=1 | MEF2D | 0.046261 | 55.20991 |
| 159 | Q3ZBT5 | sp\|Q3ZBT5\|STX7_BOVIN Syntaxin-7 OS=Bos taurus OX=9913 GN=STX7 PE=2 SV=1;tr\|A0A3Q1NGJ6\|A0A3Q1NGJ6_BOVIN Syntaxin-7 OS=Bos taurus OX=9913 GN=STX7 PE=3 SV=1 | STX7 | 0.046545 | -44.9868 |
| 160 | E1BMU6 | tr\|E1BMU6\|E1BMU6_BOVIN A-kinase anchoring protein 1 OS=Bos taurus OX=9913 GN=AKAP1 PE=4 SV=1 | AKAP1 | 0.046682 | -98.9285 |
| 161 | Q9N180 | tr\|Q9N180\|Q9N180_BOVIN Ankyrin 1 (Fragment) OS=Bos taurus OX=9913 PE=2 SV=1 | #VALUE! | 0.047489 | -87.9452 |
| 162 | A0A3Q1MHH3 | tr\|A0A3Q1MHH3\|A0A3Q1MHH3_BOVIN Sorbin and SH3 domain containing 1 OS=Bos taurus OX=9913 GN=SORBS1 PE=4 SV=1;tr\|A0A3Q1LLW5\|A0A3Q1LLW5_BOVIN Sorbin and SH3 domain containing 1 OS=Bos taurus OX=9913 GN=SORBS1 PE=4 SV=1;tr\|A0A3Q1MMC9\|A0A3Q1MMC9_BOVIN Sorbin an | SORBS1 | 0.047572 | 68.22271 |
| 163 | G5E5C0 | tr\|G5E5C0\|G5E5C0_BOVIN Uncharacterized protein OS=Bos taurus OX=9913 GN=INF2 PE=4 SV=2 | INF2 | 0.047738 | -2.2989 |
| 164 | F6PXE3 | tr\|F6PXE3\|F6PXE3_BOVIN Caveolae associated protein 2 OS=Bos taurus OX=9913 GN=CAVIN2 PE=1 SV=1;tr\|Q17QZ6\|Q17QZ6_BOVIN SDPR protein (Fragment) OS=Bos taurus OX=9913 GN=SDPR PE=2 SV=1 | CAVIN2 | 0.047773 | 124.2 |
| 165 | A0A3Q1N7L7 | tr\|A0A3Q1N7L7\|A0A3Q1N7L7_BOVIN Adducin 1 OS=Bos taurus OX=9913 GN=ADD1 PE=3 SV=1;tr\|A0A3Q1LZD7\|A0A3Q1LZD7_BOVIN Adducin 1 OS=Bos taurus OX=9913 GN=ADD1 PE=3 SV=1 | ADD1 | 0.04778 | 53.26311 |
| 166 | P98201 | sp\|P98201\|NFAC1_BOVIN Nuclear factor of activated T-cells, cytoplasmic 1 OS=Bos taurus OX=9913 GN=NFATC1 PE=1 SV=2;tr\|A0A3Q1M5J8\|A0A3Q1M5J8_BOVIN Nuclear factor of-activated T-cells, cytoplasmic 1 OS=Bos taurus OX=9913 GN=NFATC1 PE=4 SV=1;tr\|A0A3Q1LGN8\|A0A | NFATC1 | 0.048274 | 60.16778 |
| 167 | P28800 | sp\|P28800\|A2AP_BOVIN Alpha-2-antiplasmin OS=Bos taurus OX=9913 GN=SERPINF2 PE=1 SV=2 | SERPIN | 0.048288 | -126.706 |
| 168 | A0A3Q1ML67 | tr\|A0A3Q1ML67\|A0A3Q1ML67_BOVIN WAPL cohesin release factor OS=Bos taurus OX=9913 GN=WAPL PE=3 SV=1;tr\|E1BGC3\|E1BGC3_BOVIN WAPL cohesin release factor OS=Bos taurus OX=9913 GN=WAPL PE=3 SV=1 | WAPL | 0.048889 | 80.57842 |
| 169 | A6QP60 | tr\|A6QP60\|A6QP60_BOVIN Uncharacterized protein (Fragment) OS=Bos taurus OX=9913 PE=2 SV=1;tr\|A0A3Q1M8Q7\|A0A3Q1M8Q7_BOVIN Extended synaptotagmin 2 OS=Bos taurus OX=9913 GN=ESYT2 PE=1 SV=1;tr\|F1MN29\|F1MN29_BOVIN Extended synaptotagmin 2 OS=Bos taurus OX=9913 | ESYT2 | 0.048965 | 52.2989 |

**Supplementary Table 7**: Significant proteins in ALA vs. CTL adipose tissue

| S.no | Protein ID | Fasta headers | Gene | p-value | Fold Change |
| --- | --- | --- | --- | --- | --- |
| 1 | A7E337 | tr\|A7E337\|A7E337_BOVIN OCRL protein OS=Bos taurus OX=9913 GN=OCRL PE=2 SV=1;tr\|A0A3Q1N3L2\|A0A3Q1N3L2_BOVIN Phosphoinositide 5-phosphatase OS=Bos taurus OX=9913 GN=OCRL PE=3 SV=1;tr\|F1MBY5\|F1MBY5_BOVIN Phosphoinositide 5-phosphatase OS=Bos taurus OX=9913 GN | OCRL | 0.023415 | -48.9061 |
| 2 | P02638 | sp\|P02638\|S100B_BOVIN Protein S100-B OS=Bos taurus OX=9913 GN=S100B PE=1 SV=2 | S100B | 0.001592 | -15.3462 |
| 3 | P55918 | sp\|P55918\|MFAP4_BOVIN Microfibril-associated glycoprotein 4 OS=Bos taurus OX=9913 GN=MFAP4 PE=1 SV=2 | MFAP4 | 0.000647 | -13.8346 |
| 4 | P19483 | sp\|P19483\|ATPA_BOVIN ATP synthase subunit alpha, mitochondrial OS=Bos taurus OX=9913 GN=ATP5F1A PE=1 SV=1 | ATP5F1 | 0.002276 | -13.8191 |
| 5 | P01030 | sp\|P01030\|CO4_BOVIN Complement C4 (Fragments) OS=Bos taurus OX=9913 GN=C4 PE=1 SV=2 | C4 | 0.0002 | -13.3456 |
| 6 | A5PK45 | sp\|A5PK45\|GT251_BOVIN Procollagen galactosyltransferase 1 OS=Bos taurus OX=9913 GN=COLGALT1 PE=2 SV=1;tr\|A0A3Q1N2V9\|A0A3Q1N2V9_BOVIN Procollagen galactosyltransferase 1 OS=Bos taurus OX=9913 GN=COLGALT1 PE=3 SV=1 | COLGAL | 0.00274 | -9.19629 |
| 7 | Q0VCY8 | tr\|Q0VCY8\|Q0VCY8_BOVIN 15 kDa phosphoprotein enriched in astrocytes OS=Bos taurus OX=9913 GN=PEA15 PE=2 SV=1 | PEA15 | 0.004142 | -8.84655 |
| 8 | A0A3Q1M3L6 | tr\|A0A3Q1M3L6\|A0A3Q1M3L6_BOVIN Uncharacterized protein OS=Bos taurus OX=9913 PE=1 SV=1 |  | 0.014754 | -7.55004 |
| 9 | Q9TVC5 | tr\|Q9TVC5\|Q9TVC5_BOVIN Leukocyte antigen OS=Bos taurus OX=9913 GN=BoLA-DRB3 PE=2 SV=1;tr\|Q30253\|Q30253_BOVIN DR beta-chain antigen binding domain (Fragment) OS=Bos taurus OX=9913 GN=Bota-DRB22 PE=4 SV=2;tr\|E7FLN2\|E7FLN2_BOVIN Major histocompatibility compl | BoLA-D | 0.031928 | -7.11605 |
| 10 | Q1RMR8 | tr\|Q1RMR8\|Q1RMR8_BOVIN Reticulon OS=Bos taurus OX=9913 GN=RTN4 PE=1 SV=1 | RTN4 | 0.012199 | -7.07125 |
| 11 | A0A3Q1MHC2 | tr\|A0A3Q1MHC2\|A0A3Q1MHC2_BOVIN Ubiquitin specific peptidase 40 OS=Bos taurus OX=9913 GN=USP40 PE=4 SV=1;tr\|E1BKD2\|E1BKD2_BOVIN Ubiquitin specific peptidase 40 OS=Bos taurus OX=9913 GN=USP40 PE=4 SV=2;tr\|A0A3Q1N8D6\|A0A3Q1N8D6_BOVIN Ubiquitin specific peptid | USP40 | 0.016363 | -6.60549 |
| 12 | F1N610 | tr\|F1N610\|F1N610_BOVIN Ig-like domain-containing protein OS=Bos taurus OX=9913 GN=BLA-DQB PE=4 SV=1;tr\|Q5XWC9\|Q5XWC9_BOVIN MHC class II antigen OS=Bos taurus OX=9913 GN=BoLA-DQB PE=2 SV=1;tr\|Q3ZC29\|Q3ZC29_BOVIN MHC class II antigen OS=Bos taurus OX=9913 GN | BLA-DQ | 0.002944 | -6.23868 |
| 13 | Q0VCM0 | sp\|Q0VCM0\|S10AG_BOVIN Protein S100-A16 OS=Bos taurus OX=9913 GN=S100A16 PE=3 SV=1 | S100A1 | 0.040044 | -6.20401 |
| 14 | P79105 | sp\|P79105\|S10AC_BOVIN Protein S100-A12 OS=Bos taurus OX=9913 GN=S100A12 PE=1 SV=3;tr\|A0A452DJ46\|A0A452DJ46_BOVIN Protein S100 OS=Bos taurus OX=9913 GN=S100A12 PE=1 SV=1;tr\|B6VAP8\|B6VAP8_BOVIN Protein S100 OS=Bos taurus OX=9913 GN=S100A12 PE=2 SV=1;tr\|A0A3S | S100A1 | 0.012119 | -6.19141 |
| 15 | Q3SZI2 | tr\|Q3SZI2\|Q3SZI2_BOVIN Lamin A/C OS=Bos taurus OX=9913 GN=LMNA PE=2 SV=1 | LMNA | 0.04246 | -5.54997 |
| 16 | Q7YRW9 | tr\|Q7YRW9\|Q7YRW9_BOVIN Reticulon OS=Bos taurus OX=9913 GN=RTN4 PE=1 SV=2 | RTN4 | 0.000744 | -5.27473 |
| 17 | G9HQQ8 | tr\|G9HQQ8\|G9HQQ8_BOVIN MHC class II antigen (Fragment) OS=Bos taurus OX=9913 GN=BoLA-DQA PE=4 SV=1;tr\|G9HQQ6\|G9HQQ6_BOVIN MHC class II antigen (Fragment) OS=Bos taurus OX=9913 GN=BoLA-DQA PE=4 SV=1;tr\|Q0WXH9\|Q0WXH9_BOVIN MHC class II antigen (Fragment) OS= | BoLA-D | 0.004666 | -5.23666 |
| 18 | E1BN85 | tr\|E1BN85\|E1BN85_BOVIN GTF2I repeat domain containing 1 OS=Bos taurus OX=9913 GN=GTF2IRD1 PE=4 SV=3;tr\|A0A3Q1MKQ2\|A0A3Q1MKQ2_BOVIN GTF2I repeat domain containing 1 OS=Bos taurus OX=9913 GN=GTF2IRD1 PE=4 SV=1;tr\|A0A3Q1MG11\|A0A3Q1MG11_BOVIN GTF2I repeat doma | GTF2IR | 0.000737 | -5.08234 |
| 19 | A0A3Q1N2F2 | tr\|A0A3Q1N2F2\|A0A3Q1N2F2_BOVIN Uncharacterized protein OS=Bos taurus OX=9913 GN=LOC100848700 PE=4 SV=1;tr\|A0A3Q1LJM0\|A0A3Q1LJM0_BOVIN Uncharacterized protein OS=Bos taurus OX=9913 GN=LOC100848700 PE=4 SV=1;tr\|A0A3Q1LPI1\|A0A3Q1LPI1_BOVIN Uncharacterized pro | LOC100 | 0.013694 | -5.03561 |
| 20 | CON__P02662 | ;tr\|A0A3Q1NG86\|A0A3Q1NG86_BOVIN Alpha-S1-casein OS=Bos taurus OX=9913 GN=CSN1S1 PE=1 SV=1;sp\|P02662\|CASA1_BOVIN Alpha-S1-casein OS=Bos taurus OX=9913 GN=CSN1S1 PE=1 SV=2;tr\|B5B3R8\|B5B3R8_BOVIN Alpha-S1-casein OS=Bos taurus OX=9913 GN=CSN1S1 PE=2 SV=1;tr\|A0 | CSN1S1 | 0.02294 | -4.86842 |
| 21 | A2I7M9 | sp\|A2I7M9\|SPA32_BOVIN Serpin A3-2 OS=Bos taurus OX=9913 GN=SERPINA3-2 PE=3 SV=1 | SERPIN | 0.00179 | -4.81221 |
| 22 | P35720 | sp\|P35720\|C560_BOVIN Succinate dehydrogenase cytochrome b560 subunit, mitochondrial OS=Bos taurus OX=9913 GN=SDHC PE=1 SV=2;tr\|A0A3Q1MMN4\|A0A3Q1MMN4_BOVIN Succinate dehydrogenase cytochrome b560 subunit, mitochondrial OS=Bos taurus OX=9913 GN=SDHC PE=3 SV= | SDHC | 6.70E-05 | -4.7068 |
| 23 | F1MMD7 | tr\|F1MMD7\|F1MMD7_BOVIN Inter-alpha-trypsin inhibitor heavy chain H4 OS=Bos taurus OX=9913 GN=ITIH4 PE=3 SV=3;tr\|A0A3Q1LZ09\|A0A3Q1LZ09_BOVIN Inter-alpha-trypsin inhibitor heavy chain H4 OS=Bos taurus OX=9913 GN=ITIH4 PE=3 SV=1;sp\|Q3T052\|ITIH4_BOVIN Inter-al | ITIH4 | 0.012954 | -4.62852 |
| 24 | A0A452DJ87 | tr\|A0A452DJ87\|A0A452DJ87_BOVIN Carboxypeptidase OS=Bos taurus OX=9913 GN=CTSA PE=3 SV=1;sp\|Q3MI05\|PPGB_BOVIN Lysosomal protective protein OS=Bos taurus OX=9913 GN=CTSA PE=2 SV=1;tr\|A6BML7\|A6BML7_BOVIN Carboxypeptidase OS=Bos taurus OX=9913 GN=PPGB PE=3 SV= | CTSA | 0.003055 | -4.48452 |
| 25 | Q28851 | sp\|Q28851\|ATPK_BOVIN ATP synthase subunit f, mitochondrial OS=Bos taurus OX=9913 GN=ATP5MF PE=1 SV=3;tr\|A0A3Q1N4G2\|A0A3Q1N4G2_BOVIN ATP synthase membrane subunit f OS=Bos taurus OX=9913 PE=3 SV=1;tr\|A0A3Q1ML66\|A0A3Q1ML66_BOVIN ATP synthase membrane subunit | ATP5MF | 0.042525 | -4.36912 |
| 26 | F1MYX2 | tr\|F1MYX2\|F1MYX2_BOVIN Apolipoprotein M OS=Bos taurus OX=9913 GN=APOM PE=3 SV=1;tr\|Q3ZBQ9\|Q3ZBQ9_BOVIN Apolipoprotein M OS=Bos taurus OX=9913 GN=APOM PE=2 SV=1;tr\|A0A3Q1MB70\|A0A3Q1MB70_BOVIN Apolipoprotein M OS=Bos taurus OX=9913 GN=APOM PE=3 SV=1 | APOM | 0.004093 | -4.26773 |
| 27 | A0A3Q1ME09 | tr\|A0A3Q1ME09\|A0A3Q1ME09_BOVIN Microtubule actin crosslinking factor 1 OS=Bos taurus OX=9913 GN=MACF1 PE=1 SV=1;tr\|F1N6H4\|F1N6H4_BOVIN Microtubule actin crosslinking factor 1 OS=Bos taurus OX=9913 GN=MACF1 PE=1 SV=3 | MACF1 | 0.027168 | -4.01177 |
| 28 | CON__Q32MB2 |  |  | 0.009364 | -3.95897 |
| 29 | Q28194 | tr\|Q28194\|Q28194_BOVIN Thrombospondin-1 (Fragment) OS=Bos taurus OX=9913 PE=2 SV=1 |  | 0.019273 | -3.76721 |
| 30 | A0A3Q1MP66 | tr\|A0A3Q1MP66\|A0A3Q1MP66_BOVIN ATP synthase subunit b OS=Bos taurus OX=9913 PE=3 SV=1 |  | 0.014413 | -3.75445 |
| 31 | F1MRZ8 | tr\|F1MRZ8\|F1MRZ8_BOVIN Pleckstrin OS=Bos taurus OX=9913 GN=PLEK PE=4 SV=2 | PLEK | 0.00932 | -3.72977 |
| 32 | Q5GN72 | tr\|Q5GN72\|Q5GN72_BOVIN Alpha-1-acid glycoprotein OS=Bos taurus OX=9913 GN=agp PE=2 SV=2;sp\|Q3SZR3\|A1AG_BOVIN Alpha-1-acid glycoprotein OS=Bos taurus OX=9913 GN=ORM1 PE=2 SV=1;;tr\|A0A3Q1LS55\|A0A3Q1LS55_BOVIN Alpha-1-acid glycoprotein OS=Bos taurus OX=9913 G | agp | 0.026523 | -3.72725 |
| 33 | A5D7G6 | tr\|A5D7G6\|A5D7G6_BOVIN Dolichyl-diphosphooligosaccharide--protein glycotransferase OS=Bos taurus OX=9913 GN=STT3B PE=2 SV=1 | STT3B | 0.001585 | -3.63145 |
| 34 | A0A0N9SE96 | tr\|A0A0N9SE96\|A0A0N9SE96_BOVIN ATP synthase F0 subunit 6 (Fragment) OS=Bos taurus OX=9913 GN=ATP6 PE=4 SV=1;tr\|A0A481U5Q0\|A0A481U5Q0_BOVIN ATP synthase subunit a (Fragment) OS=Bos taurus OX=9913 GN=ATP6 PE=3 SV=1;tr\|A0A481U7V0\|A0A481U7V0_BOVIN ATP synthase | ATP6 | 0.039686 | -3.59386 |
| 35 | Q5E9F8 | sp\|Q5E9F8\|H33_BOVIN Histone H3.3 OS=Bos taurus OX=9913 GN=H3-3A PE=1 SV=3;tr\|A0A3Q1M588\|A0A3Q1M588_BOVIN Histone domain-containing protein OS=Bos taurus OX=9913 PE=3 SV=1;tr\|G3MYD7\|G3MYD7_BOVIN Histone H3 OS=Bos taurus OX=9913 GN=LOC100297725 PE=3 SV=1;sp\| | H3-3A | 0.050874 | -3.49639 |
| 36 | P13213 | sp\|P13213\|SPRC_BOVIN SPARC OS=Bos taurus OX=9913 GN=SPARC PE=1 SV=2;tr\|A0A3Q1N541\|A0A3Q1N541_BOVIN Osteonectin OS=Bos taurus OX=9913 GN=SPARC PE=3 SV=1 | SPARC | 0.014704 | -3.4516 |
| 37 | A0A452DIG5 | tr\|A0A452DIG5\|A0A452DIG5_BOVIN Microsomal glutathione S-transferase 3 OS=Bos taurus OX=9913 GN=MGST3 PE=4 SV=1;sp\|Q3T100\|MGST3_BOVIN Microsomal glutathione S-transferase 3 OS=Bos taurus OX=9913 GN=MGST3 PE=2 SV=1;tr\|A0A4Y5RXV0\|A0A4Y5RXV0_BOVIN Microsomal g | MGST3 | 0.000983 | -3.41199 |
| 38 | Q09TE3 | tr\|Q09TE3\|Q09TE3_BOVIN Insulin-like growth factor binding protein acid labile subunit OS=Bos taurus OX=9913 GN=IGFALS PE=2 SV=1;tr\|M5FKF4\|M5FKF4_BOVIN Insulin-like growth factor binding protein, acid labile subunit OS=Bos taurus OX=9913 GN=IGFALS PE=4 SV=1 | IGFALS | 0.037256 | -3.39458 |
| 39 | A5D7D9 | tr\|A5D7D9\|A5D7D9_BOVIN RNA helicase OS=Bos taurus OX=9913 GN=DHX15 PE=1 SV=1 | DHX15 | 0.041692 | -3.36341 |
| 40 | P01966 | sp\|P01966\|HBA_BOVIN Hemoglobin subunit alpha OS=Bos taurus OX=9913 GN=HBA PE=1 SV=2;;tr\|A0A1K0FUD3\|A0A1K0FUD3_BOVIN Globin C1 OS=Bos taurus OX=9913 GN=GLNC1 PE=3 SV=1;tr\|A0A452DIQ5\|A0A452DIQ5_BOVIN GLOBIN domain-containing protein OS=Bos taurus OX=9913 GN= | HBA | 0.032218 | -3.31776 |
| 41 | P60902 | sp\|P60902\|S10AA_BOVIN Protein S100-A10 OS=Bos taurus OX=9913 GN=S100A10 PE=1 SV=2 | S100A1 | 0.00212 | -3.10173 |
| 42 | P68401 | sp\|P68401\|PA1B2_BOVIN Platelet-activating factor acetylhydrolase IB subunit alpha2 OS=Bos taurus OX=9913 GN=PAFAH1B2P68402 PE=1 SV=1;tr\|V6F7P3\|V6F7P3_BOVIN Platelet-activating factor acetylhydrolase IB subunit beta OS=Bos taurus OX=9913 GN=PAFAH1B2 PE=4 SV | PAFAH1 | 7.67E-05 | -3.02581 |
| 43 | P18902 | sp\|P18902\|RET4_BOVIN Retinol-binding protein 4 OS=Bos taurus OX=9913 GN=RBP4 PE=1 SV=1;tr\|G1K122\|G1K122_BOVIN Retinol-binding protein OS=Bos taurus OX=9913 GN=RBP4 PE=3 SV=1;tr\|A0A3Q1MSW9\|A0A3Q1MSW9_BOVIN Plasma retinol-binding protein OS=Bos taurus OX=991 | RBP4 | 0.020493 | -2.98314 |
| 44 | P41976 | sp\|P41976\|SODM_BOVIN Superoxide dismutase [Mn], mitochondrial OS=Bos taurus OX=9913 GN=SOD2 PE=2 SV=1;tr\|E1BHL1\|E1BHL1_BOVIN Superoxide dismutase [Mn], mitochondrial OS=Bos taurus OX=9913 GN=SOD2 PE=3 SV=3 | SOD2 | 0.001195 | -2.96561 |
| 45 | A1L543 | tr\|A1L543\|A1L543_BOVIN Carboxypeptidase OS=Bos taurus OX=9913 GN=SCPEP1 PE=2 SV=1;tr\|Q2NKZ9\|Q2NKZ9_BOVIN Carboxypeptidase OS=Bos taurus OX=9913 GN=SCPEP1 PE=2 SV=1;tr\|A0A3Q1LQC4\|A0A3Q1LQC4_BOVIN Carboxypeptidase OS=Bos taurus OX=9913 GN=SCPEP1 PE=3 SV=1 | SCPEP1 | 0.001778 | -2.7937 |
| 46 | F1MNN7 | tr\|F1MNN7\|F1MNN7_BOVIN Lipopolysaccharide-binding protein OS=Bos taurus OX=9913 GN=LBP PE=3 SV=2;sp\|Q2TBI0\|LBP_BOVIN Lipopolysaccharide-binding protein OS=Bos taurus OX=9913 GN=LBP PE=2 SV=1;tr\|A0A3Q1LKB0\|A0A3Q1LKB0_BOVIN Lipopolysaccharide-binding protein | LBP | 0.027923 | -2.70947 |
| 47 | Q3SZP5 | sp\|Q3SZP5\|ACOX1_BOVIN Peroxisomal acyl-coenzyme A oxidase 1 OS=Bos taurus OX=9913 GN=ACOX1 PE=2 SV=1;tr\|A0A3Q1MI26\|A0A3Q1MI26_BOVIN Acyl-coenzyme A oxidase OS=Bos taurus OX=9913 GN=ACOX1 PE=1 SV=1 | ACOX1 | 0.025191 | -2.69136 |
| 48 | Q5E963 | sp\|Q5E963\|ARP5L_BOVIN Actin-related protein 2/3 complex subunit 5-like protein OS=Bos taurus OX=9913 GN=ARPC5L PE=2 SV=1 | ARPC5L | 0.010321 | -2.64735 |
| 49 | Q29423 | sp\|Q29423\|CD44_BOVIN CD44 antigen OS=Bos taurus OX=9913 GN=CD44 PE=2 SV=1;tr\|Q9TQU9\|Q9TQU9_BOVIN CD44 protein (Fragment) OS=Bos taurus OX=9913 GN=cd44 PE=2 SV=1 | CD44 | 0.003009 | -2.63492 |
| 50 | Q2YDM1 | sp\|Q2YDM1\|ARL1_BOVIN ADP-ribosylation factor-like protein 1 OS=Bos taurus OX=9913 GN=ARL1 PE=2 SV=1 | ARL1 | 0.042415 | -2.60596 |
| 51 | A1L539 | tr\|A1L539\|A1L539_BOVIN Glypican 6 OS=Bos taurus OX=9913 GN=GPC6 PE=2 SV=1 | GPC6 | 0.001695 | -2.5565 |
| 52 | Q32PB9 | sp\|Q32PB9\|RL38_BOVIN 60S ribosomal protein L38 OS=Bos taurus OX=9913 GN=RPL38 PE=3 SV=4 | RPL38 | 0.036084 | -2.49343 |
| 53 | P02070 | sp\|P02070\|HBB_BOVIN Hemoglobin subunit beta OS=Bos taurus OX=9913 GN=HBB PE=1 SV=1;tr\|D4QBB4\|D4QBB4_BOVIN Globin A1 OS=Bos taurus OX=9913 GN=HBB PE=3 SV=1;;tr\|D4QBB3\|D4QBB3_BOVIN Hemoglobin beta OS=Bos taurus OX=9913 GN=HBB PE=3 SV=1 | HBB | 0.039586 | -2.42125 |
| 54 | Q4U5R4 | sp\|Q4U5R4\|RN114_BOVIN E3 ubiquitin-protein ligase RNF114 OS=Bos taurus OX=9913 GN=RNF114 PE=2 SV=1 | RNF114 | 0.004116 | -2.37277 |
| 55 | Q76I82 | sp\|Q76I82\|RS15A_BOVIN 40S ribosomal protein S15a OS=Bos taurus OX=9913 GN=RPS15A PE=2 SV=1;tr\|G3N3S3\|G3N3S3_BOVIN 40S ribosomal protein S15a OS=Bos taurus OX=9913 PE=3 SV=1;tr\|E1BLY1\|E1BLY1_BOVIN 40S ribosomal protein S15a OS=Bos taurus OX=9913 PE=3 SV=1;t | RPS15A | 0.041843 | -2.2148 |
| 56 | A7Z055 | tr\|A7Z055\|A7Z055_BOVIN PLAA protein OS=Bos taurus OX=9913 GN=PLAA PE=2 SV=1 | PLAA | 0.016628 | -2.20461 |
| 57 | A0A3Q1LPF4 | tr\|A0A3Q1LPF4\|A0A3Q1LPF4_BOVIN Uroporphyrinogen decarboxylase OS=Bos taurus OX=9913 GN=UROD PE=3 SV=1;tr\|E1BEX4\|E1BEX4_BOVIN Uroporphyrinogen decarboxylase OS=Bos taurus OX=9913 GN=UROD PE=3 SV=1 | UROD | 0.004584 | -2.19391 |
| 58 | A8YXY3 | sp\|A8YXY3\|SEP15_BOVIN Selenoprotein F OS=Bos taurus OX=9913 GN=SELENOF PE=2 SV=2 | SELENO | 0.022477 | -2.17528 |
| 59 | Q8MJ81 | tr\|Q8MJ81\|Q8MJ81_BOVIN Apoptosis regulator Bcl-2 (Fragment) OS=Bos taurus OX=9913 GN=bcl2 PE=3 SV=1;sp\|O02718\|BCL2_BOVIN Apoptosis regulator Bcl-2 OS=Bos taurus OX=9913 GN=BCL2 PE=2 SV=1;tr\|F6R2C4\|F6R2C4_BOVIN Apoptosis regulator Bcl-2 OS=Bos taurus OX=991 | bcl2 | 0.00994 | -2.13784 |
| 60 | Q2HJ23 | sp\|Q2HJ23\|MLP3A_BOVIN Microtubule-associated proteins 1A/1B light chain 3A OS=Bos taurus OX=9913 GN=MAP1LC3A PE=2 SV=1;tr\|A0A3Q1MTK8\|A0A3Q1MTK8_BOVIN Microtubule-associated proteins 1A/1B light chain 3A OS=Bos taurus OX=9913 GN=MAP1LC3A PE=3 SV=1 | MAP1LC | 0.006367 | -2.03723 |
| 61 | P12234 | sp\|P12234\|MPCP_BOVIN Phosphate carrier protein, mitochondrial OS=Bos taurus OX=9913 GN=SLC25A3 PE=1 SV=1 | SLC25A | 0.000191 | -1.9803 |
| 62 | F1MUL0 | tr\|F1MUL0\|F1MUL0_BOVIN Integrin-associated protein OS=Bos taurus OX=9913 GN=CD47 PE=4 SV=3;sp\|Q9N0K1\|CD47_BOVIN Leukocyte surface antigen CD47 OS=Bos taurus OX=9913 GN=CD47 PE=2 SV=1;tr\|Q08DW0\|Q08DW0_BOVIN Integrin-associated protein OS=Bos taurus OX=9913 | CD47 | 0.010898 | -1.9643 |
| 63 | G8JKW7 | tr\|G8JKW7\|G8JKW7_BOVIN Serpin A3-7 OS=Bos taurus OX=9913 GN=SERPINA3-7 PE=3 SV=2 | SERPIN | 0.014329 | -1.95604 |
| 64 | F1MGJ4 | tr\|F1MGJ4\|F1MGJ4_BOVIN Signal transducer and activator of transcription OS=Bos taurus OX=9913 GN=STAT6 PE=3 SV=1;tr\|Q9N0E5\|Q9N0E5_BOVIN Signal transducer and activator of transcription 6 (Fragment) OS=Bos taurus OX=9913 GN=STAT6 PE=2 SV=1 | STAT6 | 0.045299 | -1.93763 |
| 65 | Q2HJ58 | sp\|Q2HJ58\|PRPS1_BOVIN Ribose-phosphate pyrophosphokinase 1 OS=Bos taurus OX=9913 GN=PRPS1 PE=2 SV=3;tr\|G3MY14\|G3MY14_BOVIN Ribose-phosphate diphosphokinase OS=Bos taurus OX=9913 GN=PRPS2 PE=3 SV=2;tr\|A0A3Q1MHS1\|A0A3Q1MHS1_BOVIN Ribose-phosphate diphosphoki | PRPS1 | 0.035134 | -1.92112 |
| 66 | G1K152 | tr\|G1K152\|G1K152_BOVIN Thioredoxin-related transmembrane protein 2 OS=Bos taurus OX=9913 GN=TMX2 PE=4 SV=2;sp\|Q2TBU2\|TMX2_BOVIN Thioredoxin-related transmembrane protein 2 OS=Bos taurus OX=9913 GN=TMX2 PE=2 SV=1 | TMX2 | 0.036819 | -1.85769 |
| 67 | A6H769 | sp\|A6H769\|RS7_BOVIN 40S ribosomal protein S7 OS=Bos taurus OX=9913 GN=RPS7 PE=2 SV=1;tr\|A0A3Q1M657\|A0A3Q1M657_BOVIN 40S ribosomal protein S7 OS=Bos taurus OX=9913 GN=RPS7 PE=1 SV=1 | RPS7 | 0.015092 | -1.81898 |
| 68 | Q5BIN5 | sp\|Q5BIN5\|PIN1_BOVIN Peptidyl-prolyl cis-trans isomerase NIMA-interacting 1 OS=Bos taurus OX=9913 GN=PIN1 PE=2 SV=1 | PIN1 | 0.02624 | -1.81302 |
| 69 | Q2KIT8 | tr\|Q2KIT8\|Q2KIT8_BOVIN GDP-4-keto-6-deoxy-D-mannose-3,5-epimerase-4-reductase OS=Bos taurus OX=9913 GN=TSTA3 PE=2 SV=1;tr\|A0A3S5ZPJ7\|A0A3S5ZPJ7_BOVIN GDP-4-keto-6-deoxy-D-mannose-3,5-epimerase-4-reductase OS=Bos taurus OX=9913 GN=GFUS PE=1 SV=1;tr\|A0A3Q1M2 | TSTA3 | 0.038898 | -1.79168 |
| 70 | A4FUZ1 | tr\|A4FUZ1\|A4FUZ1_BOVIN Lactoylglutathione lyase OS=Bos taurus OX=9913 GN=GLO1 PE=2 SV=1;tr\|A0A3Q1LS06\|A0A3Q1LS06_BOVIN Lactoylglutathione lyase OS=Bos taurus OX=9913 PE=3 SV=1 | GLO1 | 3.37E-07 | -1.76232 |
| 71 | A0A3Q1LM63 | tr\|A0A3Q1LM63\|A0A3Q1LM63_BOVIN NIF3-like protein 1 OS=Bos taurus OX=9913 GN=NIF3L1 PE=3 SV=1;sp\|Q05B89\|NIF3L_BOVIN NIF3-like protein 1 OS=Bos taurus OX=9913 GN=NIF3L1 PE=2 SV=1;tr\|A0A3Q1LG04\|A0A3Q1LG04_BOVIN NIF3-like protein 1 OS=Bos taurus OX=9913 GN=NIF | NIF3L1 | 8.65E-05 | -1.73182 |
| 72 | Q2TBN5 | sp\|Q2TBN5\|COMD9_BOVIN COMM domain-containing protein 9 OS=Bos taurus OX=9913 GN=COMMD9 PE=2 SV=1;tr\|F1MIC5\|F1MIC5_BOVIN COMM domain-containing protein 9 OS=Bos taurus OX=9913 GN=COMMD9 PE=4 SV=1 | COMMD9 | 0.021256 | -1.68185 |
| 73 | P00171 | sp\|P00171\|CYB5_BOVIN Cytochrome b5 OS=Bos taurus OX=9913 GN=CYB5A PE=1 SV=3;tr\|A0A452DIG0\|A0A452DIG0_BOVIN Cytochrome b5 OS=Bos taurus OX=9913 GN=CYB5A PE=1 SV=1 | CYB5A | 0.008585 | -1.59407 |
| 74 | Q3ZBT6 | sp\|Q3ZBT6\|TRA2B_BOVIN Transformer-2 protein homolog beta OS=Bos taurus OX=9913 GN=TRA2B PE=2 SV=1 | TRA2B | 0.031257 | -1.59366 |
| 75 | Q3SZJ7 | tr\|Q3SZJ7\|Q3SZJ7_BOVIN Lysosomal-associated membrane protein 2 OS=Bos taurus OX=9913 GN=LAMP2 PE=1 SV=1;tr\|A0A3Q1MTN6\|A0A3Q1MTN6_BOVIN Lysosomal associated membrane protein 2 OS=Bos taurus OX=9913 GN=LAMP2 PE=1 SV=1;tr\|G3MXJ5\|G3MXJ5_BOVIN Lysosomal associa | LAMP2 | 0.040715 | -1.59355 |
| 76 | Q3ZCH9 | sp\|Q3ZCH9\|HDHD2_BOVIN Haloacid dehalogenase-like hydrolase domain-containing protein 2 OS=Bos taurus OX=9913 GN=HDHD2 PE=2 SV=1 | HDHD2 | 0.013309 | -1.56268 |
| 77 | P62958 | sp\|P62958\|HINT1_BOVIN Histidine triad nucleotide-binding protein 1 OS=Bos taurus OX=9913 GN=HINT1 PE=1 SV=2;tr\|A0A3Q1MQJ9\|A0A3Q1MQJ9_BOVIN HIT domain-containing protein OS=Bos taurus OX=9913 PE=4 SV=1;tr\|A0A452DIE9\|A0A452DIE9_BOVIN Histidine triad nucleoti | HINT1 | 0.025191 | -1.55185 |
| 78 | A5PK65 | sp\|A5PK65\|DOPD_BOVIN D-dopachrome decarboxylase OS=Bos taurus OX=9913 GN=DDT PE=3 SV=1;tr\|A0A0F7RQ40\|A0A0F7RQ40_BOVIN D-dopachrome tautomerase OS=Bos taurus OX=9913 GN=DDT PE=3 SV=1;tr\|A0A3Q1MAM8\|A0A3Q1MAM8_BOVIN D-dopachrome decarboxylase OS=Bos taurus OX | DDT | 0.01771 | 2.019958 |
| 79 | Q58D70 | tr\|Q58D70\|Q58D70_BOVIN Serine/threonine-protein phosphatase OS=Bos taurus OX=9913 GN=PPP2CA PE=2 SV=1;sp\|Q0P594\|PP2AB_BOVIN Serine/threonine-protein phosphatase 2A catalytic subunit beta isoform OS=Bos taurus OX=9913 GN=PPP2CB PE=1 SV=1;sp\|P67774\|PP2AA_BOV | PPP2CA | 0.008526 | 2.031305 |
| 80 | E1BAK6 | tr\|E1BAK6\|E1BAK6_BOVIN DAZ associated protein 1 OS=Bos taurus OX=9913 GN=DAZAP1 PE=4 SV=3;tr\|A0A3Q1M8V6\|A0A3Q1M8V6_BOVIN DAZ associated protein 1 OS=Bos taurus OX=9913 GN=DAZAP1 PE=4 SV=1 | DAZAP1 | 0.00077 | 2.051456 |
| 81 | Q3SZA0 | sp\|Q3SZA0\|CSN4_BOVIN COP9 signalosome complex subunit 4 OS=Bos taurus OX=9913 GN=COPS4 PE=2 SV=1 | COPS4 | 0.022232 | 2.080509 |
| 82 | A6QNJ7 | tr\|A6QNJ7\|A6QNJ7_BOVIN PGM5 protein OS=Bos taurus OX=9913 GN=PGM5 PE=2 SV=1;tr\|A1L598\|A1L598_BOVIN Phosphoglucomutase 5 (Fragment) OS=Bos taurus OX=9913 GN=PGM5 PE=2 SV=1 | PGM5 | 0.0095 | 2.112223 |
| 83 | P00428 | sp\|P00428\|COX5B_BOVIN Cytochrome c oxidase subunit 5B, mitochondrial OS=Bos taurus OX=9913 GN=COX5B PE=1 SV=2;tr\|A0A3Q1MBD7\|A0A3Q1MBD7_BOVIN Cytochrome c oxidase polypeptide Vb OS=Bos taurus OX=9913 GN=COX5B PE=3 SV=1 | COX5B | 0.001964 | 2.15212 |
| 84 | F2Z4H3 | tr\|F2Z4H3\|F2Z4H3_BOVIN DNA-directed RNA polymerases I, II, and III subunit RPABC3 OS=Bos taurus OX=9913 GN=POLR2H PE=1 SV=1;tr\|A8QQK8\|A8QQK8_BOVIN POLR2H protein OS=Bos taurus OX=9913 GN=POLR2H PE=2 SV=1 | POLR2H | 0.028508 | 2.159271 |
| 85 | Q9TTJ5 | sp\|Q9TTJ5\|RGN_BOVIN Regucalcin OS=Bos taurus OX=9913 GN=RGN PE=2 SV=1;tr\|A0A3Q1MLX2\|A0A3Q1MLX2_BOVIN Gluconolactonase OS=Bos taurus OX=9913 GN=RGN PE=3 SV=1 | RGN | 0.032577 | 2.193811 |
| 86 | Q7YR75 | sp\|Q7YR75\|RM12_BOVIN 39S ribosomal protein L12, mitochondrial OS=Bos taurus OX=9913 GN=MRPL12 PE=1 SV=1;tr\|A5PJ86\|A5PJ86_BOVIN MRPL12 protein OS=Bos taurus OX=9913 GN=MRPL12 PE=2 SV=1 | MRPL12 | 0.030115 | 2.211881 |
| 87 | A9ZTJ9 | tr\|A9ZTJ9\|A9ZTJ9_BOVIN Calcineurin B (Fragment) OS=Bos taurus OX=9913 PE=2 SV=1;tr\|A0A452DIC7\|A0A452DIC7_BOVIN Calcineurin subunit B type 1 OS=Bos taurus OX=9913 GN=PPP3R1 PE=3 SV=1;sp\|P63099\|CANB1_BOVIN Calcineurin subunit B type 1 OS=Bos taurus OX=9913 G | PPP3R1 | 0.047213 | 2.262295 |
| 88 | Q08DW1 | tr\|Q08DW1\|Q08DW1_BOVIN RAB9A, member RAS oncogene family OS=Bos taurus OX=9913 GN=RAB9A PE=2 SV=1;tr\|Q0VCB4\|Q0VCB4_BOVIN RAB9B, member RAS oncogene family OS=Bos taurus OX=9913 GN=RAB9B PE=2 SV=1 | RAB9A | 0.030091 | 2.357863 |
| 89 | F1MZD8 | tr\|F1MZD8\|F1MZD8_BOVIN Palmdelphin OS=Bos taurus OX=9913 GN=PALMD PE=3 SV=2;tr\|A0A3Q1MIU2\|A0A3Q1MIU2_BOVIN Palmdelphin OS=Bos taurus OX=9913 GN=PALMD PE=3 SV=1;sp\|Q3MHH7\|PALMD_BOVIN Palmdelphin OS=Bos taurus OX=9913 GN=PALMD PE=2 SV=1 | PALMD | 0.022984 | 2.365596 |
| 90 | Q08DM3 | tr\|Q08DM3\|Q08DM3_BOVIN Malic enzyme OS=Bos taurus OX=9913 GN=ME2 PE=2 SV=1;tr\|A0A3Q1MWG7\|A0A3Q1MWG7_BOVIN Malic enzyme OS=Bos taurus OX=9913 GN=ME2 PE=3 SV=1 | ME2 | 0.026967 | 2.384248 |
| 91 | Q3SYR7 | sp\|Q3SYR7\|RL9_BOVIN 60S ribosomal protein L9 OS=Bos taurus OX=9913 GN=RPL9 PE=2 SV=1;tr\|A0A3Q1ME53\|A0A3Q1ME53_BOVIN 60S ribosomal protein L9 OS=Bos taurus OX=9913 PE=3 SV=1;tr\|F6PWD5\|F6PWD5_BOVIN 60S ribosomal protein L9 OS=Bos taurus OX=9913 PE=3 SV=2;tr\| | RPL9 | 0.031901 | 2.387436 |
| 92 | A0A3Q1N4H7 | tr\|A0A3Q1N4H7\|A0A3Q1N4H7_BOVIN Tankyrase 1 binding protein 1 OS=Bos taurus OX=9913 GN=TNKS1BP1 PE=1 SV=1;tr\|E1BP50\|E1BP50_BOVIN Tankyrase 1 binding protein 1 OS=Bos taurus OX=9913 GN=TNKS1BP1 PE=1 SV=2 | TNKS1B | 0.011704 | 2.428398 |
| 93 | A0A3Q1MPD9 | tr\|A0A3Q1MPD9\|A0A3Q1MPD9_BOVIN Syntaxin 2 OS=Bos taurus OX=9913 GN=STX2 PE=3 SV=1;tr\|A0A3Q1NEX5\|A0A3Q1NEX5_BOVIN Syntaxin 2 OS=Bos taurus OX=9913 GN=STX2 PE=3 SV=1;tr\|F1MNQ8\|F1MNQ8_BOVIN Syntaxin 2 OS=Bos taurus OX=9913 GN=STX2 PE=3 SV=3 | STX2 | 0.030512 | 2.429952 |
| 94 | A6H732 | tr\|A6H732\|A6H732_BOVIN TMX4 protein OS=Bos taurus OX=9913 GN=TMX4 PE=2 SV=1 | TMX4 | 0.001719 | 2.438966 |
| 95 | E1BMK7 | tr\|E1BMK7\|E1BMK7_BOVIN GB1/RHD3-type G domain-containing protein OS=Bos taurus OX=9913 GN=LOC107131333 PE=3 SV=3 | LOC107 | 0.019134 | 2.447588 |
| 96 | Q32LL2 | sp\|Q32LL2\|STML2_BOVIN Stomatin-like protein 2, mitochondrial OS=Bos taurus OX=9913 GN=STOML2 PE=2 SV=1;tr\|A5PJA6\|A5PJA6_BOVIN Stomatin (EPB72)-like 2 OS=Bos taurus OX=9913 GN=STOML2 PE=2 SV=1 | STOML2 | 0.044915 | 2.462199 |
| 97 | CON__P00761 |  |  | 0.001614 | 2.468564 |
| 98 | A6QNM9 | tr\|A6QNM9\|A6QNM9_BOVIN SLC25A12 protein OS=Bos taurus OX=9913 GN=SLC25A12 PE=2 SV=1;tr\|A0A3Q1MLV2\|A0A3Q1MLV2_BOVIN Solute carrier family 25 member 12 OS=Bos taurus OX=9913 GN=SLC25A12 PE=3 SV=1;tr\|A0A3Q1M9V5\|A0A3Q1M9V5_BOVIN Solute carrier family 25 member | SLC25A | 0.024444 | 2.479146 |
| 99 | Q56JY0 | sp\|Q56JY0\|HIKES_BOVIN Protein Hikeshi OS=Bos taurus OX=9913 GN=HIKESHI PE=2 SV=1 | HIKESH | 0.03383 | 2.491557 |
| 100 | Q2KIA2 | sp\|Q2KIA2\|TR112_BOVIN Multifunctional methyltransferase subunit TRM112-like protein OS=Bos taurus OX=9913 GN=TRMT112 PE=2 SV=1;tr\|G3MYM3\|G3MYM3_BOVIN Multifunctional methyltransferase subunit TRM112-like protein OS=Bos taurus OX=9913 PE=3 SV=2 | TRMT11 | 0.016383 | 2.516618 |
| 101 | A0A3Q1LRU3 | tr\|A0A3Q1LRU3\|A0A3Q1LRU3_BOVIN 2,4-dienoyl-CoA reductase 2 OS=Bos taurus OX=9913 GN=DECR2 PE=1 SV=1;tr\|M5FHY5\|M5FHY5_BOVIN 2,4-dienoyl CoA reductase 2 OS=Bos taurus OX=9913 GN=DECR2 PE=3 SV=1;tr\|Q3ZBW6\|Q3ZBW6_BOVIN 2,4-dienoyl CoA reductase 2, peroxisomal | DECR2 | 0.024034 | 2.549137 |
| 102 | A0A3Q1MLE7 | tr\|A0A3Q1MLE7\|A0A3Q1MLE7_BOVIN Syntaxin binding protein 3 OS=Bos taurus OX=9913 GN=STXBP3 PE=3 SV=1;tr\|A0A3Q1MWU8\|A0A3Q1MWU8_BOVIN Syntaxin binding protein 3 OS=Bos taurus OX=9913 GN=STXBP3 PE=3 SV=1;tr\|F1MXB4\|F1MXB4_BOVIN Syntaxin binding protein 3 OS=Bos | STXBP3 | 0.035505 | 2.598331 |
| 103 | A0A3Q1LPE3 | tr\|A0A3Q1LPE3\|A0A3Q1LPE3_BOVIN Propionyl-CoA carboxylase beta chain, mitochondrial OS=Bos taurus OX=9913 GN=PCCB PE=4 SV=1;sp\|Q2TBR0\|PCCB_BOVIN Propionyl-CoA carboxylase beta chain, mitochondrial OS=Bos taurus OX=9913 GN=PCCB PE=2 SV=1;tr\|A0A452DIR0\|A0A452 | PCCB | 0.039139 | 2.704277 |
| 104 | A0A3Q1LUD3 | tr\|A0A3Q1LUD3\|A0A3Q1LUD3_BOVIN Dihydropyrimidine dehydrogenase [NADP(+)] OS=Bos taurus OX=9913 GN=DPYD PE=3 SV=1;tr\|A0A3Q1MC58\|A0A3Q1MC58_BOVIN Dihydropyrimidine dehydrogenase [NADP(+)] OS=Bos taurus OX=9913 GN=DPYD PE=3 SV=1;sp\|Q28007\|DPYD_BOVIN Dihydropy | DPYD | 0.041458 | 2.704587 |
| 105 | Q32PI9 | sp\|Q32PI9\|MPZL1_BOVIN Myelin protein zero-like protein 1 OS=Bos taurus OX=9913 GN=MPZL1 PE=2 SV=1 | MPZL1 | 0.048585 | 2.71714 |
| 106 | E1BJG5 | tr\|E1BJG5\|E1BJG5_BOVIN Tripartite motif containing 28 OS=Bos taurus OX=9913 GN=TRIM28 PE=4 SV=3 | TRIM28 | 0.016277 | 2.84944 |
| 107 | Q862D4 | tr\|Q862D4\|Q862D4_BOVIN L-lactate dehydrogenase (Fragment) OS=Bos taurus OX=9913 PE=2 SV=1 |  | 0.0315 | 2.855353 |
| 108 | Q29460 | sp\|Q29460\|PA1B3_BOVIN Platelet-activating factor acetylhydrolase IB subunit alpha1 OS=Bos taurus OX=9913 GN=PAFAH1B3 PE=1 SV=1 | PAFAH1 | 0.032243 | 2.899754 |
| 109 | A4IFE3 | sp\|A4IFE3\|ACTY_BOVIN Beta-centractin OS=Bos taurus OX=9913 GN=ACTR1B PE=2 SV=1;tr\|G3N132\|G3N132_BOVIN Beta-centractin OS=Bos taurus OX=9913 GN=ACTR1B PE=3 SV=2 | ACTR1B | 0.030932 | 2.929956 |
| 110 | A0A3Q1LSR3 | tr\|A0A3Q1LSR3\|A0A3Q1LSR3_BOVIN Uncharacterized protein OS=Bos taurus OX=9913 PE=4 SV=1;tr\|A0A3Q1LUV3\|A0A3Q1LUV3_BOVIN GTP-binding protein Rheb OS=Bos taurus OX=9913 GN=RHEB PE=4 SV=1;tr\|A0A3Q1N4Q1\|A0A3Q1N4Q1_BOVIN GTP-binding protein Rheb OS=Bos taurus OX= | RHEB | 0.018424 | 2.97137 |
| 111 | Q32S33 | tr\|Q32S33\|Q32S33_BOVIN Transporter 2 ATP-binding cassette sub-family B OS=Bos taurus OX=9913 GN=TAP2 PE=3 SV=1;tr\|Q8SQ31\|Q8SQ31_BOVIN ABC transporter TAP2 OS=Bos taurus OX=9913 PE=2 SV=1;tr\|F1MCG5\|F1MCG5_BOVIN Uncharacterized protein OS=Bos taurus OX=9913 | TAP2 | 0.046817 | 2.994656 |
| 112 | Q3MHH4 | sp\|Q3MHH4\|SYQ_BOVIN Glutamine--tRNA ligase OS=Bos taurus OX=9913 GN=QARS1 PE=2 SV=1 | QARS1 | 0.03574 | 3.033575 |
| 113 | Q3MHE6 | tr\|Q3MHE6\|Q3MHE6_BOVIN SF3B4 protein (Fragment) OS=Bos taurus OX=9913 GN=SF3B4 PE=2 SV=1;tr\|F1MWR2\|F1MWR2_BOVIN Splicing factor 3b subunit 4 OS=Bos taurus OX=9913 GN=SF3B4 PE=3 SV=1 | SF3B4 | 0.038551 | 3.091624 |
| 114 | CON__P48668 | ;;;tr\|M0QVY0\|M0QVY0_BOVIN IF rod domain-containing protein OS=Bos taurus OX=9913 GN=KRT6A PE=3 SV=1;tr\|A4FV94\|A4FV94_BOVIN KRT6A protein OS=Bos taurus OX=9913 GN=KRT6A PE=2 SV=1;tr\|F1MUY2\|F1MUY2_BOVIN IF rod domain-containing protein OS=Bos taurus OX=9913 | KRT6A | 0.043963 | 3.129315 |
| 115 | Q0VCA3 | sp\|Q0VCA3\|LETM1_BOVIN Mitochondrial proton/calcium exchanger protein OS=Bos taurus OX=9913 GN=LETM1 PE=2 SV=1;tr\|A0A3Q1MMS8\|A0A3Q1MMS8_BOVIN Leucine zipper-EF-hand-containing transmembrane protein 1 OS=Bos taurus OX=9913 GN=LETM1 PE=3 SV=1;tr\|A0A3Q1MIY6\|A0 | LETM1 | 0.016564 | 3.140636 |
| 116 | A0A452DJS8 | tr\|A0A452DJS8\|A0A452DJS8_BOVIN Tubulin polymerization-promoting protein family member 3 OS=Bos taurus OX=9913 GN=TPPP3 PE=3 SV=1;sp\|Q3ZCC8\|TPPP3_BOVIN Tubulin polymerization-promoting protein family member 3 OS=Bos taurus OX=9913 GN=TPPP3 PE=1 SV=1 | TPPP3 | 0.019361 | 3.166892 |
| 117 | Q2NL38 | tr\|Q2NL38\|Q2NL38_BOVIN Dodecenoyl-Coenzyme A delta isomerase (3,2 trans-enoyl-Coenzyme A isomerase) OS=Bos taurus OX=9913 GN=DCI PE=2 SV=1;tr\|M5FJY5\|M5FJY5_BOVIN 3,2-trans-enoyl-CoA isomerase, mitochondrial OS=Bos taurus OX=9913 GN=DCI PE=4 SV=1;tr\|F1MTT7\| | DCI | 0.049746 | 3.184538 |
| 118 | P01252 | sp\|P01252\|PTMA_BOVIN Prothymosin alpha OS=Bos taurus OX=9913 GN=PTMA PE=1 SV=2 | PTMA | 0.005944 | 3.250901 |
| 119 | Q2HJI9 | tr\|Q2HJI9\|Q2HJI9_BOVIN Ring-box 1 OS=Bos taurus OX=9913 GN=RBX1 PE=2 SV=1;tr\|A0A3Q1LNF8\|A0A3Q1LNF8_BOVIN RING-type domain-containing protein OS=Bos taurus OX=9913 GN=LOC780968 PE=3 SV=1;tr\|A0A3Q1MWJ8\|A0A3Q1MWJ8_BOVIN Uncharacterized protein OS=Bos taurus O | RBX1 | 0.036516 | 3.254314 |
| 120 | A0A3Q1LXG9 | tr\|A0A3Q1LXG9\|A0A3Q1LXG9_BOVIN von Willebrand factor A domain-containing protein 1 OS=Bos taurus OX=9913 GN=VWA1 PE=4 SV=1;sp\|A6QLN9\|VWA1_BOVIN von Willebrand factor A domain-containing protein 1 OS=Bos taurus OX=9913 GN=VWA1 PE=2 SV=1;tr\|A0A452DJ03\|A0A452 | VWA1 | 0.007482 | 3.258727 |
| 121 | F1N430 | tr\|F1N430\|F1N430_BOVIN Metalloproteinase inhibitor 2 OS=Bos taurus OX=9913 GN=TIMP2 PE=3 SV=2;sp\|P16368\|TIMP2_BOVIN Metalloproteinase inhibitor 2 OS=Bos taurus OX=9913 GN=TIMP2 PE=1 SV=2 | TIMP2 | 0.012099 | 3.278643 |
| 122 | Q3T0V3 | sp\|Q3T0V3\|EIF3K_BOVIN Eukaryotic translation initiation factor 3 subunit K OS=Bos taurus OX=9913 GN=EIF3K PE=2 SV=1 | EIF3K | 0.02389 | 3.280717 |
| 123 | F1MD79 | tr\|F1MD79\|F1MD79_BOVIN Ankyrin repeat and FYVE domain containing 1 OS=Bos taurus OX=9913 GN=ANKFY1 PE=4 SV=3;tr\|A0A3Q1MD15\|A0A3Q1MD15_BOVIN Ankyrin repeat and FYVE domain containing 1 OS=Bos taurus OX=9913 GN=ANKFY1 PE=4 SV=1 | ANKFY1 | 0.002227 | 3.286582 |
| 124 | F1N3S4 | tr\|F1N3S4\|F1N3S4_BOVIN Cullin 4A OS=Bos taurus OX=9913 GN=CUL4A PE=3 SV=3;tr\|A0A3Q1MNL1\|A0A3Q1MNL1_BOVIN Cullin 4A OS=Bos taurus OX=9913 GN=CUL4A PE=3 SV=1 | CUL4A | 0.000181 | 3.405041 |
| 125 | A0A3Q1LTW5 | tr\|A0A3Q1LTW5\|A0A3Q1LTW5_BOVIN Dynamin GTPase OS=Bos taurus OX=9913 GN=DNM1 PE=3 SV=1;tr\|A0A3Q1N4X4\|A0A3Q1N4X4_BOVIN Dynamin GTPase OS=Bos taurus OX=9913 GN=DNM1 PE=3 SV=1;tr\|A0A3Q1LR08\|A0A3Q1LR08_BOVIN Dynamin GTPase OS=Bos taurus OX=9913 GN=DNM1 PE=3 SV= | DNM1 | 0.050053 | 3.415172 |
| 126 | A7MBC5 | tr\|A7MBC5\|A7MBC5_BOVIN Isoleucyl-tRNA synthetase OS=Bos taurus OX=9913 GN=IARS1 PE=2 SV=1 | IARS1 | 0.04872 | 3.428003 |
| 127 | A0A3Q1M010 | tr\|A0A3Q1M010\|A0A3Q1M010_BOVIN UTP--glucose-1-phosphate uridylyltransferase OS=Bos taurus OX=9913 GN=UGP2 PE=1 SV=1;tr\|A0A3Q1MUI0\|A0A3Q1MUI0_BOVIN UTP--glucose-1-phosphate uridylyltransferase OS=Bos taurus OX=9913 GN=UGP2 PE=1 SV=1 | UGP2 | 0.000129 | 3.431898 |
| 128 | Q08DH9 | tr\|Q08DH9\|Q08DH9_BOVIN CCCTC-binding factor OS=Bos taurus OX=9913 GN=CTCF PE=2 SV=1 | CTCF | 0.005708 | 3.431949 |
| 129 | A7MBI8 | tr\|A7MBI8\|A7MBI8_BOVIN NUDT9 protein OS=Bos taurus OX=9913 GN=NUDT9 PE=2 SV=1 | NUDT9 | 0.004277 | 3.458028 |
| 130 | F1MYV9 | tr\|F1MYV9\|F1MYV9_BOVIN Non-specific serine/threonine protein kinase OS=Bos taurus OX=9913 GN=OXSR1 PE=1 SV=1;tr\|Q1JQD5\|Q1JQD5_BOVIN Non-specific serine/threonine protein kinase OS=Bos taurus OX=9913 GN=OXSR1 PE=2 SV=1 | OXSR1 | 0.005367 | 3.648275 |
| 131 | A0A3Q1LZL7 | tr\|A0A3Q1LZL7\|A0A3Q1LZL7_BOVIN Uncharacterized protein OS=Bos taurus OX=9913 GN=IFI16 PE=4 SV=1;tr\|A0A3Q1MUY5\|A0A3Q1MUY5_BOVIN Uncharacterized protein OS=Bos taurus OX=9913 GN=IFI16 PE=4 SV=1;tr\|E1BNK6\|E1BNK6_BOVIN Uncharacterized protein OS=Bos taurus OX= | IFI16 | 0.007911 | 3.73266 |
| 132 | A0A3Q1LPL8 | tr\|A0A3Q1LPL8\|A0A3Q1LPL8_BOVIN Thioredoxin related transmembrane protein 3 OS=Bos taurus OX=9913 GN=TMX3 PE=4 SV=1;tr\|A6QL97\|A6QL97_BOVIN TMX3 protein OS=Bos taurus OX=9913 GN=TMX3 PE=2 SV=1 | TMX3 | 0.001119 | 3.853651 |
| 133 | Q0P559 | tr\|Q0P559\|Q0P559_BOVIN Interferon induced transmembrane protein 1 (9-27) OS=Bos taurus OX=9913 GN=IFITM1 PE=2 SV=1;tr\|G5E542\|G5E542_BOVIN Uncharacterized protein OS=Bos taurus OX=9913 GN=LOC112444847 PE=3 SV=1;tr\|Q95MQ3\|Q95MQ3_BOVIN Interferon-induced prot | IFITM1 | 0.008365 | 3.869162 |
| 134 | P12260 | sp\|P12260\|F13A_BOVIN Coagulation factor XIII A chain (Fragment) OS=Bos taurus OX=9913 GN=F13A1 PE=1 SV=2 | F13A1 | 0.020835 | 4.00905 |
| 135 | A0A6B9SE04 | tr\|A0A6B9SE04\|A0A6B9SE04_BOVIN Ig heavy chain variable region (Fragment) OS=Bos taurus OX=9913 PE=2 SV=1 |  | 0.001045 | 4.068892 |
| 136 | Q3ZBA6 | sp\|Q3ZBA6\|DJB11_BOVIN DnaJ homolog subfamily B member 11 OS=Bos taurus OX=9913 GN=DNAJB11 PE=2 SV=1 | DNAJB1 | 0.000536 | 4.140291 |
| 137 | Q05717 | sp\|Q05717\|IBP5_BOVIN Insulin-like growth factor-binding protein 5 OS=Bos taurus OX=9913 GN=IGFBP5 PE=2 SV=2 | IGFBP5 | 0.002951 | 4.154898 |
| 138 | Q17QE2 | sp\|Q17QE2\|LMCD1_BOVIN LIM and cysteine-rich domains protein 1 OS=Bos taurus OX=9913 GN=LMCD1 PE=2 SV=1;tr\|A0A3Q1NGA7\|A0A3Q1NGA7_BOVIN LIM and cysteine-rich domains protein 1 OS=Bos taurus OX=9913 GN=LMCD1 PE=4 SV=1 | LMCD1 | 0.027373 | 4.299891 |
| 139 | Q58D71 | tr\|Q58D71\|Q58D71_BOVIN Uncharacterized protein FLJ36812 OS=Bos taurus OX=9913 GN=FLJ36812 PE=2 SV=1;tr\|Q17QC1\|Q17QC1_BOVIN LRRC57 protein OS=Bos taurus OX=9913 GN=LRRC57 PE=2 SV=1 | FLJ368 | 0.002219 | 4.357765 |
| 140 | E1BG08 | tr\|E1BG08\|E1BG08_BOVIN Axin interactor, dorsalization associated OS=Bos taurus OX=9913 GN=AIDA PE=3 SV=1 | AIDA | 0.006748 | 4.497153 |
| 141 | A0A6B9SDM9 | tr\|A0A6B9SDM9\|A0A6B9SDM9_BOVIN Ig heavy chain variable region (Fragment) OS=Bos taurus OX=9913 PE=2 SV=1 |  | 0.0084 | 5.452337 |
| 142 | M5FMT1 | tr\|M5FMT1\|M5FMT1_BOVIN Tryptase alpha/beta 1 OS=Bos taurus OX=9913 GN=TPSB1 PE=4 SV=1;tr\|A6QQ05\|A6QQ05_BOVIN TPSB1 protein OS=Bos taurus OX=9913 GN=TPSB1 PE=2 SV=1;tr\|A0A3Q1LX36\|A0A3Q1LX36_BOVIN Peptidase S1 domain-containing protein OS=Bos taurus OX=9913 | TPSB1 | 0.037716 | 5.531464 |
| 143 | Q865A2 | tr\|Q865A2\|Q865A2_BOVIN 2-5 oligoadenylate synthase OS=Bos taurus OX=9913 GN=OAS1 PE=2 SV=1;tr\|F1N6E0\|F1N6E0_BOVIN 2-5 oligoadenylate synthase OS=Bos taurus OX=9913 GN=OAS1X PE=3 SV=3 | OAS1 | 0.042223 | 9.76764 |
| 144 | A0A452DJ66 | tr\|A0A452DJ66\|A0A452DJ66_BOVIN Tubulin alpha chain OS=Bos taurus OX=9913 GN=TUBA1D PE=3 SV=1;sp\|Q2HJ86\|TBA1D_BOVIN Tubulin alpha-1D chain OS=Bos taurus OX=9913 GN=TUBA1D PE=1 SV=1;tr\|F2Z4K0\|F2Z4K0_BOVIN Tubulin alpha chain OS=Bos taurus OX=9913 GN=TUBA3E P | TUBA1D | 0.022705 | 11.09711 |

**Supplementary table 8.** Top canonical pathway analysis of adipose tissue phosphoproteome supplemented with ALA and the molecules involved in the pathways with up/down regulation

| Pathways | -log(p-value) | Molecules | Up/down regulated |
| --- | --- | --- | --- |
| Protein Kinase A Signaling | 2.86 | Adducin 1 (ADD1) | up |
|  |  | A-kinase anchoring protein 1 (AKAP1) | down |
|  |  | A-kinase anchoring protein 12 (AKAP12) | up |
|  |  | Filamin A (FLNA) | down |
|  |  | MHC class I region proline-rich protein CAT53 (PPP1R10) | down |
|  |  | Paxillin (PXN) | down |
|  |  | Nuclear factor of activated T-cells, cytoplasmic 1 (NFATC1) | up |
|  |  | cAMP-dependent protein kinase type I-alpha regulatory subunit (PRKAR1A) | up |
| RHOA Signaling | 2.39 | ABL2 | up |
|  |  | MPRIP | down |
|  |  | MSN | up |
|  |  | CDC42EP4 | up |
| Signaling by Rho Family GTPases | 1.88 | ARHGEF17 | down |
|  |  | FNBP1 | down |
|  |  | MSN | up |
|  |  | CDC42EP4 | up |
|  |  | VIM | up |
| Xenobiotic Metabolism PXR Signaling Pathway | 1.73 | HSP90AB1 | down |
|  |  | PPP1R10 | down |
|  |  | PRKAR1A | up |
|  |  | NCOR2 | up |
| ILK Signaling | 1.68 | FLNA | down |
|  |  | PXN | down |
|  |  | VIM | up |
|  |  | FNBP1 | down |
| CDK5 Signaling | 1.66 | CABLES1 | up |
|  |  | PRKAR1A | up |
|  |  | PPP1R10 | down |
| Cholecystokinin/Gastrin-mediated Signaling | 1.61 | FNBP1 | down |
|  |  | PXN | down |
|  |  | MEF2D | up |
| p38 MAPK Signaling | 1.6 | DAXX | down |
|  |  | MEF2D | up |
|  |  | HMGN1 | down |
| LXR/RXR Activation | 1.58 | AHSG | up |
|  |  | SERPINF2 | down |
|  |  | NCOR2 | down |
| ERK/MAPK Signaling | 1.57 | NFATC1 | up |
|  |  | PRKAR1A | up |
|  |  | PXN | down |
|  |  | PPP1R10 | down |
| Calcium Signaling | 1.56 | HDAC4 | up |
|  |  | NFATC1 | up |
|  |  | PRKAR1A | up |
|  |  | MEF2D | down |
| Insulin Receptor Signaling | 1.43 | PPP1R10 | down |
|  |  | SYNJ1 | up |
|  |  | PRKAR1A | up |
| AMPK Signaling | 1.41 | PFKL | up |
|  |  | SMARCC2 | down |
|  |  | ULK1 | up |
|  |  | PRKAR1A | up |
| Corticotropin Releasing Hormone Signaling | 1.35 | MEF2D | up |
|  |  | SMARCC2 | up |
|  |  | PRKAR1A | up |

**Supplementary Table 9.** Top canonical pathway analysis of adipose tissue proteome supplemented with ALA and the molecules involved in the pathways with up/down regulation

| Pathways | -log (p-value) | Molecules | Up/down regulated |
| --- | --- | --- | --- |
| Oxidative Phosphorylation | 4.8 | ATP5F1A | down |
|  |  | ATP5MF | down |
|  |  | COX5B | up |
|  |  | CYB5A | down |
|  |  | MT-ATP6 | down |
|  |  | SDHC | down |
| Mitochondrial Dysfunction | 4.72 | ATP5F1A | down |
|  |  | ATP5MF | down |
|  |  | COX5B | up |
|  |  | SDHC | down |
|  |  | SOD2 | down |
|  |  | CYB5A | down |
|  |  | MT-ATP6 | down |
| Acute Phase Response Signaling | 3.57 | ITIH4 | down |
|  |  | LBP | down |
|  |  | ORM1 | up |
|  |  | SOD2 | down |
|  |  | RBP4 | down |
|  |  | SERPINA3 | down |
| LXR/RXR Activation | 3.5 | APOM | down |
|  |  | LBP | down |
|  |  | ORM1 | up |
|  |  | ITIH4 | down |
|  |  | RBP4 | down |
| NER (Nucleotide Excision Repair) Enhanced Pathway | 2.82 | COPS4 | up |
|  |  | POLR2H | up |
|  |  | RBX1 | up |
|  |  | CUL4A | up |
| Clathrin-mediated Endocytosis Signaling | 2.62 | APOM | down |
|  |  | DNM1 | up |
|  |  | ORM1 | up |
|  |  | RBP4 | down |
|  |  | ARPC5L | down |
| Sirtuin Signaling Pathway | 2.52 | ATP5F1A | down |
|  |  | MT-ATP6 | down |
|  |  | SDHC | down |
|  |  | SOD2 | down |
|  |  | TRIM28 | up |
|  |  | MAP1LC3A | down |
| FXR/RXR Activation | 2.5 | APOM | down |
|  |  | RBP4 | down |
|  |  | ITIH4, | down |
|  |  | ORM1 | up |
| IL-12 Signaling and Production in Macrophages | 2.38 | APOM | down |
|  |  | RBP4 | down |
|  |  | STAT6 | down |
|  |  | ORM1 | up |
| IL-4 Signaling | 1.99 | HLA-DQB1 | down |
|  |  | OCRL | down |
|  |  | STAT6 | down |
| Production of Nitric Oxide and Reactive Oxygen Species in Macrophages | 1.87 | APOM | down |
|  |  | PPP2CA | up |
|  |  | ORM1 | up |
|  |  | RBP4 | down |
| NRF2-mediated Oxidative Stress Response | 1.56 | DNAJB11 | up |
|  |  | RBX1 | up |
|  |  | SOD2 | down |
|  |  | MGST3 | down |
| Protein Ubiquitination Pathway | 1.36 | DNAJB11 | up |
|  |  | TAP2 | up |
|  |  | USP40 | down |
|  |  | RBX1 | up |


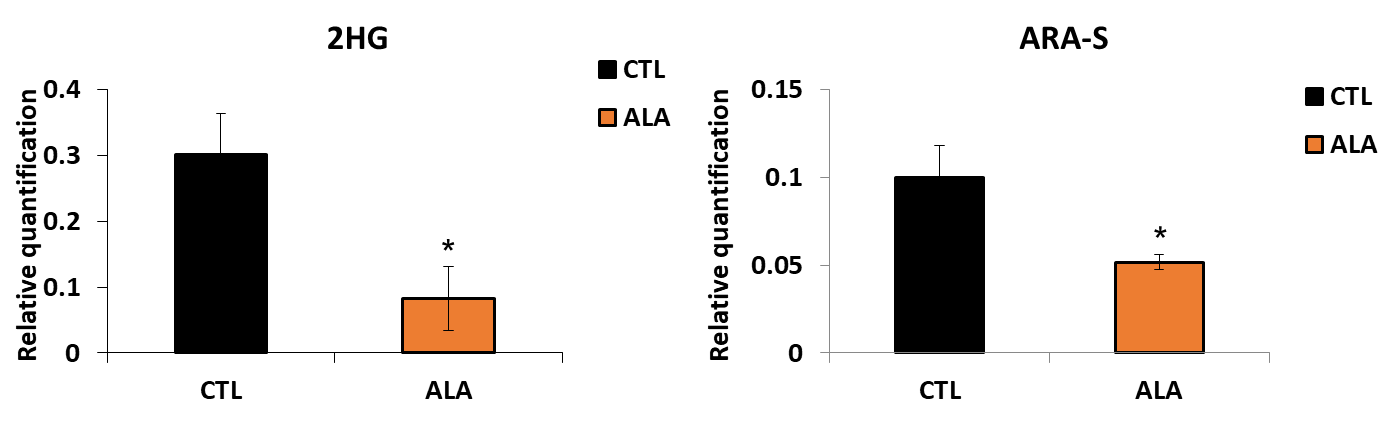


**Supplementary Figure 2**. **Relative endocannabinoid-like compound levels in plasma of postpartum (PP) dairy cows supplemented with ALA.** * *P* < 0.05; n=5. Blood samples were taken 20 min after glucose infusion at 5-8 d PP. Dairy cows were divided into two nutritional groups from -21- 60 days PP; (i) CTL – saturated fat, (ii) ALA –flaxseed supplement providing α-linolenic acid (ALA). 2HG: 2-Dihomo-gamma-Lineoyl Glycerol; ARA-S: N-Arachidonoyl Serine.

**Supplementary Table 10.** Protein abundance in insulin-stimulated adipose tissue of postpartum (PP) dairy cows supplemented with ALA.

|  | **Treatments^1^** | | |  | |  | | |  | | |  | |
| --- | --- | --- | --- | --- | --- | --- | --- | --- | --- | --- | --- | --- | --- |
|  | **CTL** | **ALA** | | | | | **SEM** | | | ***P*-value** | | |  |
| **Protein** |  | |  | |  | | |  | | |  | |  |
| **Endocannabinoid system** | | | | | | | | | | | | |  |
| *CB1^2^* | 3.3 | | 3.1 | | 0.50 | | | | | 0.73 | | |  |
| *CB2^3^* | 2.6 | | 1.3 | | 0.96 | | | | | 0.36 | | |  |
| *MGLL^4^* | 5.3 | | 2.3 | | 0.93 | | | | | 0.05 | | |  |
| *DAGLA^5^* | 7.2 | | 6.8 | | 2.52 | | | | | 0.91 | | |  |
| *FAAH^6^* | 6.6 | | 3.9 | | 1.09 | | | | | 0.11 | | |  |
| **Proteomics validation**   \| *ERK1/2^7^* \| 1.3 \| 0.6 \| 0.28 \| 0.12 \|  \| \| --- \| --- \| --- \| --- \| --- \| --- \| \| *pERK1/2^8^* \| 0.5 \| 0.7 \| 0.18 \| 0.45 \|  \| \| *pERK/ERK* \| 0.5 \| 0.3 \| 0.12 \| 0.33 \|  \| \| *AMPK^9^* \| 3.6 \| 1.3 \| 0.73 \| 0.06 \|  \| \| *pAMPK^10^* \| 4.0 \| 2.9 \| 0.92 \| 0.43 \|  \| \| *pAMPK/AMPK* \| 2.0 \| 2.7 \| 0.99 \| 0.65 \|  \| \| *AKT^11^* \| 4.6 \| 4.9 \| 1.05 \| 0.83 \|  \| \| *pAKT^12^* \| 6.6 \| 3.3 \| 1.28 \| 0.10 \|  \| \| *α-Tubulin* \| 1.0^a^ \| 1.6^b^ \| 0.13 \| 0.01 \|  \| \| **Adipose metabolism** \|  \|  \|  \|  \|  \| \| *FASN^13^* \| 4.0 \| 4.3 \| 0.93 \| 0.81 \|  \| \| *HSL^14^* \| 1.3 \| 0.6 \| 0.36 \| 0.23 \|  \| \| *pHSL^15^* \| 0.9 \| 0.7 \| 0.21 \| 0.52 \|  \| \| *pHSL/HSL* \| 0.8 \| 1.1 \| 0.26 \| 0.52 \|  \| \| **Inflammation** \|  \|  \|  \|  \|  \| \| *TNF-α^16^* \| 3.2^a^ \| 2.0^b^ \| 0.33 \| 0.03 \|  \| \| *RELA^17^* \| 5.5^a^ \| 1.7^b^ \| 0.65 \| <0.01 \|  \| | | | | | | | | | | | | |  |

^a-b^ Values with different superscript letters in a row are significantly different at *P* < 0.05.

^1^Dairy cows were divided into two nutritional groups from -21- 60 days PP; (i) CTL–saturated fat, (ii) ALA–flaxseed supplement providing α-linolenic acid (ALA). n = 7 in CTL and n=5 in ALA. ^2^Cannabinoid Receptor 1; ^3^Cannabinoid Receptor 2; ^4^Monoglyceride Lipase; ^5^N-Acyl Phosphatidylethanolamine Phospholipase D; ^6^Fatty Acid Amide Hydrolase; ^7^ Extracellular signal-regulated kinase; ^8^Phospho-extracellular signal regulated kinase; ^9^ Protein Kinase AMP-Activated Catalytic Subunit Alpha 1; ^10^ Phospho- Protein Kinase AMP-Activated Catalytic Subunit Alpha 1; ^11^ AKT Serine/Threonine Kinase 1; ^12^Phospho- AKT Serine (Ser473)/Threonine Kinase 1; ^13^Fatty acid synthase; ^14^ Hormone sensitive lipase; ^15^Phospho-hormone sensitive lipase (Ser660); ^16^Tumor necrosis factor alpha; ^17^Nuclear factor NF-kappa-B p65 subunit.
